# What Is Gender Dysphoria? A Critical Systematic Narrative Review

**DOI:** 10.1089/trgh.2018.0014

**Published:** 2018-11-01

**Authors:** Zowie Davy, Michael Toze

**Affiliations:** ^1^ Centre for LGBTQ Research, Health and Life Sciences, De Montfort University, Leicester, United Kingdom.; ^2^ School of Health and Social Care, University of Lincoln, Lincoln, United Kingdom.

**Keywords:** gender dysphoria, diagnostic controversies, trans people, intersex, DSM-5

## Abstract

In the fifth edition of the Diagnostic and Statistical Manual of Mental Disorders, the American Psychiatric Association has changed the diagnosis of gender identity disorder to gender dysphoria (GD). In this critical narrative review we ask: What is gender dysphoria? We report on some of the inconsistencies in the articles that foreground distress while obfuscating the fact that not all trans and intersex people suffer stress or impaired functioning, and the inappropriate referencing to intersex people in the diagnostic criterion, claims about the GD diagnosis contributing to the depathologization of and reducing stigma surrounding trans people, the conceptualizations of “gender dysphoric” research subjects, and finally we question the etiological approaches using GD as a conceptual framework. We further suggest that there are a number of methodological issues that need to be resolved to be able to claim that the GD diagnosis can be validated. To shed light on these paradoxes and methodological issues in the *DSM-5*, we report on the content validity of GD by reviewing research articles postdiagnostic inception. These findings will contribute to the debate about the validity of GD as a diagnosis for the 21st century for those people who need to live a different gender to that assigned at birth.

## Introduction

There is an existing controversy about how diagnostic texts address medical interventions related to gender transition.^1–3^ In the fifth edition of the *Diagnostic and Statistical Manual of Mental Disorders: DSM-5* (DSM-5), the American Psychiatric Association (APA) has changed the diagnosis of gender identity disorder (GID) to gender dysphoria (GD).^4^ The *DSM-IV'*s^5^ diagnosis of GID assumes that diverse gender identities are inherently disordered and consequently perceived by many to be stigmatizing. A review of GID was, therefore, a key focus in developing the *DSM-5*'s diagnosis of GD.

On the APA's webpage titled *DSM-5 Development*, it stated that from 2007 until the end of 2012 each work group met and reviewed *DSM-IV'*s strengths and problems, from which research questions and hypotheses were developed.^6^ Moreover, they stated that literature reviews, targeted research analyses, and a review of clinical expertise followed from which a draft *DSM-5* diagnostic criterion was developed and then a final approved manual was released in May 2013.

Significantly, however, in terming the new diagnosis “gender dysphoria,” the APA adopted a term that was already well used within popular and academic discourse to describe experiences of distress within gender diverse populations, but that had not previously been a diagnosis. Making use of existing and familiar terminology with a goal to reduce pathologization is potentially counter-productive if the result is lack of clarity over how terminology is being used. In particular, there is a danger that a term that is now the name of a specific diagnosable psychiatric disorder may simultaneously be applied to individuals, populations, or experiences that do not meet diagnostic criterion and that, according to the APA, should not be considered pathological.

In this article, we explore how the concept of “gender dysphoria” has been narrativized within the scientific literature since the introduction of the *DSM-5* in 2013, with a particular focus upon how far current academic usage reflects the diagnostic criterion.

The emphasis upon distress was one of the main reasons for changing GID to GD, as indicated in a memo from the members of the work group tasked to update the diagnosis. The memo states, “This proposed name change is also consistent with the general argument that the diagnostic term should, in a more transparent way, indicate that it pertains to distress”^7(p. 902)^ as the clinical problem and not the gender identity.

The full list of “new” diagnostic indicators are “a strong desire to be rid of one's primary and/or secondary sex characteristics because of a marked incongruence with one's experienced/expressed gender,” “a strong desire for the primary and/or secondary sex characteristics of the other gender,” “a strong desire to be of the other gender,” and “a strong conviction that one has the typical feelings and reactions of the other gender.” In addition, in the *DSM-5*, GD “is associated with clinically significant impairment in social, occupational, or other important areas of functioning.”^4(p. 452–3)^

An added advantage of the name change, it was also argued, was the elimination of the pathologizing effects of the GID diagnosis.^7^ However, there is a curious caveat in the manual that clearly states that “not all individuals will experience distress as a result of […] incongruence”^4 (p. 451)^ despite a strong desire for medical interventions, such as psychological therapy, hormone therapy, and/or surgeries to alleviate what the authors assert are patients' perceived incongruence between their gender identities and bodies.^8^ In addition, there is a post-transition specifier^4 (p. 453)^ within the manual, which enables clinicians to apply a code for health insurance reimbursement for those trans and intersex people who require ongoing treatments, but have no (more) signs of dysphoria.

The diagnosis of GD, therefore, encompasses some social and psychological factors and some forms of desire within the wide ranging “symptoms.” We suggest, however, that the diagnosis contains a few fundamental paradoxes.

First, it is far from transparent how the new diagnosis of GD is supposed to fully relate to distress and not identity when distress is not necessarily present. Second, earlier in the *DSM-III*,^9^ the authors excluded intersex conditions from the diagnosis of GID; however, they could be diagnosed in the *DSM-IV*^5^ under the diagnostic category “GID Not Otherwise Specified,” which distinguished those who were experiencing severe identity conflict.^10^ The diagnosis of GD now includes intersex people comparably with trans people if their clinically assigned gender at birth causes them “distress” for at least 6 months, or they are experiencing two or more symptomatic indicators. It is unclear which indicators can capture intersex people in relation to a marked incongruence with their experienced/expressed gender and identification with *the other gender*, or desire to be of *the other gender*, or a strong conviction that one has the typical feelings and reactions of *the other gender*. What is the *DSM-5* referring to by writing “the other gender”?^4 (p. 453)^ It seems to us that there is no inherent singular “other gender” in relation to intersex people's gender.

We noted previously a statement from the APA arguing the ability to translate the findings that had been “found” during the evidence review process into a workable diagnosis. A workable diagnosis' validation should be open to public scrutiny and, moreover, would be dependent on the design, sampling strategy, weight of evidence, and most importantly research question. Despite these stated principles, the necessary details do not appear to have been released for public scrutiny. This obfuscates the difficulty of translating the many potential studies' research findings that used sophisticated research methods across the different scientific communities.^11^ The diagnosis of GD, we will argue, cannot be validated according to what the scientific community claim to be standard methodological principles.

To shed light on these paradoxes and methodological issues, we report on a narrative review wherein we sought to explore the narrativization of GD post its inception as a diagnosis within the *DSM-5*. This study is a narrative review, asking simply, What is gender dysphoria? We answer this by investigating whether and to what extent the narratives in the published literature relate to the diagnostic criterion and narrative depiction in the *DSM-5*.

We also assess how the scientific community narrativizes the concept of GD and the extent to which this is influenced and shaped by the diagnostic criterion, symptomology, and description within the *DSM-5*. We did this by conducting a multidisciplinary systematic narrative review. The rationale for this is that the GD diagnosis is often used in multiple ways in the academic and pedagogical contexts, which we suggest is inevitable because of the confusion already noted. As such, the importance of garnering an understanding of the GD diagnosis and its usage in published literature across all disciplines will highlight its potential paradigmatic influence in social, psychological, and medicolegal contexts, alongside its actual validity as a diagnosis for the 21st century for those people who need to live a different gender to that assigned at birth.

## Controversial Psychiatric Diagnostics

GD syndrome was first used in an article by Fisk,^12 (p. 388)^ who claimed that the term developed out of clinical necessity and grew in an “organic and naturalistic manner” and furthermore attended to the rise in demand from people needing to transition from their assigned birth gender. The term “dysphoria” stems from the Greek meaning difficult to bear. In the area of gender and sexuality, Kutchins and Kirk^13(p. x)^ show that within the APA's organizational processes of developing diagnoses, a “strange mix of social values, political compromise, scientific evidence, and material for insurance claim forms” is introduced. At the helm of diagnostic reform of the GID diagnosis was the chair of the work group Kenneth J. Zucker, who claimed that the change from GID to GD better describes the distress that some trans and intersex people experience when their gender identity is “incongruently” experienced.^14^

In relation to trans diagnoses, contestations have been widely documented.^1,2,13–18^ The diagnosis was developed amid demonstrations against ongoing psychiatric pathologization, by trans and intersex activists who were arguing against the draft diagnosis,^1,19^ the “therapeutic” approaches toward gender diverse children,^2,20^ and how the psychiatric diagnosis plays a fundamental part in insurance-based health care provision and legal recognition requirements in many parts of the world.^1^

Another political controversy posits skepticism on the submitted scientific evidence gathered for/by the work group for the development and “improvement” of the diagnosis.^14,21^ Creators of the GD diagnosis claimed that the science used to underpin its development was rigorous. However, it is impossible to scrutinize such claims, since the discussions, methodological processes, and promised field trials of the diagnosis have not been published.

Even if the evidence were available for scrutiny, it is likely that the meta-reviews would have been based on a heterogeneous range of methods, design, and paradigmatic approaches and although in principle this could have been eventually standardized, there would be substantial difficulties in doing so. In particular, it would be necessary to synthesize studies using a range of different terms, such as transsexual, transgender, GID, and GD into an overarching GD diagnosis. Such terms represent at once identity positions and (different) symptomatic conditions. Nonetheless, the evidence that was used was judged by the APA committee members and board of trustees to be sufficient enough to formulate the GD diagnosis.

## Science and the *DSM*

At the *DSM'*s inception, science about gender and sexuality was of little importance and diagnoses were based on moralistic clinical perceptions.^13,16,22,23^ Apparently this changed in the lead up to publishing the *DSM-III*, which was advertised as using scientific standards, empirical investigation, and data to develop diagnoses that can endure empirical scrutiny.^13^ However, critics have suggested that in the following manifestation *DSM-IV-TR*,^24^ the diagnosis of GID lacked scientific rigor, resulting in stigmatizing effects, and that the criterion constructed trans people's identities as disordered because they expressed their genders differently to stereotypical masculinities and femininities associated with their assigned gender.^14,25^

Moreover, professionals working in the field of gender transitions and diagnostics critique the previous diagnosis of GID for its dependence on fixed categories of gender and gender role expressions and behaviors and, also, argued that there was a lack of reliable and valid diagnostic criterion.^26–29^

The APA's website^30^ sets out its approach to rating “scientific data,” with double-blind randomized control trials considered to be the best. In the event of a gap in high-quality research, the evidence work groups should review are (subjective) clinical observation data and come to a consensus. Clinical data and consensus are low on the APA's scientific scale, because they are highly unreliable and dependent on the views of clinician(s). Such data are inevitably influenced by clinicians' political leanings and paradigmatic approaches to psychiatry. Clearly, it is neither practically possible nor ethically appropriate to conduct double-blind randomized control trials upon interventions related to gender transition. As a consequence, the validity that was reported^31^ underpinning the diagnosis of GD was primarily reliant upon clinical consensus: evidence that is considered by the APA to be relatively low quality and reliant on a relatively limited amount of scientific evidence drawing on the research of a small number of psychiatrists/sexologists.

There is limited evidence within the public domain of systematically evaluating the reviews that took place during the *DSM-5* revision process. Nonetheless, the change from GID to the new diagnosis of GD was pitched as a product of a democratized process, considering broader knowledges and demonstrating the diverse phenomenology of trans and intersex people.^32^ The APA offered two periods for public feedback, inviting opinion and criticism about gender issues, resulting in the third largest category to receive input for the *DSM-5*'s revision process,^33^ it is unclear how these contributions were used or assessed leading Ansara and Hegarty^21^ to conclude that the “synthesis of evidence” was created by an invisible network of researchers who contribute to the ideological delegitimization of trans (and now intersex) people's own classifications of their genders and bodies.

## Validity and Reliability

A few studies have attempted a dimensional assessment of GD in different populations. For example, Cohen-Kettenis and van Goozen^34^ reported on the psychometric properties of the Utrecht gender dysphoria scale (UGDS) that has poles that range from not dysphoric to dysphoric, and that consists of 12 questions rated on a 5-point scale. They reported that the tool successfully discriminated “male” and “female” transsexuals from same-sex controls, resulting in discriminant validity. Smith et al.^35^ also used the scale to evaluate the outcomes of gender confirmation treatment outcomes reporting that GD was totally eradicated in their sample following interventions. The Gender Identity/Gender Dysphoria Questionnaire for Adolescents and Adults (GIDYQ-AA) developed by Deogracias et al.^36(p. 371)^ said that their GD scale worked with a “bipolar continuum with a male pole and a female pole and varying degrees of gender dysphoria, gender uncertainty, and gender identity transitions between the poles.”

In a recent study,^8^ it was suggested that the two scales, although both designed to measure the degree of GD, will do so in different ways because each instrument captures only some aspects of the construct. The report submitted to the APA^37^ does not set out the methodology used to evaluate research used to develop the GD diagnosis. As such, it is impossible to assess the reliability or validity of the methodology. This maybe, in part, be due to the links not being populated on the APA website, leading us to assume that the field trials were not undertaken. There was the possibility of analyzing 10 pieces of research/reviews for establishing the GD diagnosis.^30^ The studies consisted of literature reviews and secondary data analyses. However, the studies admitted to the review were not looking at distress but at gender nonconformity. Yet, according to the *DSM-5* working group, the key distinction between *DSM-IV* and *DSM-5* is the recognition that gender nonconformity and distress are not the same things, and that gender nonconformity is not pathological in its own right. Basing the diagnosis of GD on studies of gender nonconformity, while simultaneously claiming to be depathologizing gender nonconformity, is clearly neither logical nor methodologically robust.

Quantitative researchers would normally use statistical methods to associate a new instrument's utility with existing measures in other instruments.^38^ This was not possible in this instance, since the review was a secondary analysis of (qualitative) conclusions. Qualitative tools used in empirical literature reviews and in the evaluation of secondary data analyses cannot be validated using statistical criteria.^39,40^ The qualitative validity of the GD diagnosis could be assessed, in a commonsense way, through the credibility of an explanation, interpretation, and observation if its use is consistent among clinicians and researchers, and *in the way it was intended*. This can be described loosely as content validity.^41^

There is then a possibility that content validity of GD could be determined by its consistent use in the literature and what ought to have happened during the development process of constructing the GD diagnosis. Given that GD is a diagnosis primarily derived from an asserted scientific consensus, it seems appropriate to test it through exploring the degree of scientific consensus upon what GD is. We will attempt in the results of this study to go some way in unpacking the content validity of the GD diagnosis after its inception in 2013.

## Aim(s)

This study is a narrative review, asking simply, What is gender dysphoria? We answer this research question by examining the content validity and investigating whether and to what extent the narratives in the published literature relate to the diagnostic criterion and narrative depiction in the *DSM-5*. In this article, we assess how the scientific community narrativizes the concept of GD and the extent to which this is shaped by the diagnostic criterion, symptomology, and description within the *DSM-5*. We did this by conducting a multidisciplinary systematic narrative review.

Because primary research varies within different research paradigms ontologically, epistemologically, theoretically, and ideologically,^42^ a narrative review was chosen to understand the ways in which the diagnosis of GD is understood and used in contemporary peer reviewed literature.

We took the stance that the narratives that are told about GD within the literature can be adequately understood through describing the narrative patterns therein. We assumed that the narrative patterns and the concept of GD were intrinsically entwined in such a way that the narrative impacts the concept and the concept impacts the narrative. Thus, it is now sensible to start enquiring into the diagnostic validity of GD in light of the literature that followed its *DSM-5* inception.^43^ As such, this article determines what GD is (in the literature) and then makes some observations about the conceptual rigor in the scholarly area of trans and intersex people, who continue to be, from our analysis, somewhat problematically depicted in academic fora.

The importance of garnering an understanding of GD and its usage is to highlight its potential social and paradigmatic influence within academic contexts alongside the expediency of the diagnosis for the 21st century.

## Methods

A narrative approach to the research question, what is gender dysphoria, warranted very little exclusion criteria. We wanted to explore the ways that the concept was situated in the narrative structure of the articles. The concept of GD and its narrativization would be able to uncover the ways that it is utilized, explained, analyzed, and tested for in (participant) samples, artifacts, legal statues, and policies within the published work.

The term we used was GD inclusive of the dates April 2013 and June 2016. The rationale behind choosing this yearly range was that “gender dysphoria” became the official diagnosis in the *DSM-5*.^4^ Moreover, the diagnosis would provide the framework from which an evaluation and understanding of how researchers, scholars, and clinicians were narrating the diagnosis of GD in their work.

The initial results from 46 health, psychology, science, social, and humanities databases yielded 5765 articles (Table 1). The Elton B. Stephens Company (EBSCO) host was used that searches all available databases. EBSCO host reduced the number of retrieved literature to 2554 items in the printout, due to exact duplicates being automatically removed. After mining the database results manually we removed a further 1994 articles due to more duplication, and by removing all news items, we were left with560 articles. We mined further by removing another 173 articles because they were commentaries, letters to editors, not in English, or the term GD was only present in the correspondence address, keywords, or in the name of a gender identity clinic, and did not feature in the main body of the article. This left a total of 387 articles to analyze (Supplementary Table S1).

**Table 1.** **Initial Search Results**

Database	Results	Database	Results	Database	Results
NewsBank	2258	TDX	26	SwePub	3
ScienceDirect	941	Humanities International Index	24	Research Starters	3
PsycINFO	413	Japanese Periodical Index	10	MLA International Bibliography	3
CINAHL Complete	382	British Library EThOS	6	Airiti Library eBooks & Journals	2
MEDLINE	299	Credo Reference Collections	6	Informit Health Collection	2
Newswires	268	Harvard Library Bibliographic Dataset	6	RACO	2
PsycARTICLES	216	SciELO	6	ERIC	2
Social Sciences Citation Index	167	Oxford Reference	5	RECERCAT	1
Science Citation Index	144	Arts & Humanities Citation Index	5	British Education Index	1
Business Source Complete	135	Teacher Reference Center	5	Alexander Street Press	1
LexisNexis Academic: Law Reviews	108	Art Full Text (H.W. Wilson)	4	Bibliotheksverbund Bayern	1
Food Science Source	107	Informit Humanities & Social Sciences Collection	4	JSTOR Journals	1
SPORTDiscus	86	International Bibliography of Theatre & Dance with Full Text	4	Oxford Handbooks Online	1
PsycBOOKS	40	Library, Information Science & Technology Abstracts	4		
HeinOnline	31	PsycCRITIQUES	3		
OAIster	26	RCAAP	3		

We uploaded all the articles into Endnote and each author was randomly allocated articles who then searched for the term GD. We cut and pasted the paragraphs in which GD featured and placed it into a document, then uploaded it into NVivo to code inductively. We also read each of the articles to get a sense of the overall narrative and analytical framework. Each author produced codes separately.

Each month the authors met and started to discuss the codes. We refined the coding framework at each meeting and discussed both the obvious substantive codes that we were both developing independently and the “outliers.” The coding framework began to morph over a period of 8 months into agreed upon sets of hierarchical coding, which we present in this article. Some coded paragraphs fit into more than one code and theme, depending on the narrative it told. These are important findings as they emphasize a complex often conflated and repeatedly questionable use of the diagnostic concept.

## Results

We ideographically developed a number of metadata source types to enable us to see whether there were any patterns in the ways that the discipline, the geography of the article, and the sample type impacted on the narratives of GD:

- Article type: empirical, case study, reviews (meta-syntheses, etc.), discourse (legal and medical/health care frameworks).
- Country: not applicable (reviews), multiple research sites, comparative research, and 33 countries across the world.
- Discipline: cultural, psychology, biology, law, history, social science, philosophy, bioethics, medical, surgery, and misc. therapies, health services, and religion.
- Referred to *DSM-5* criteria: yes/no.
- Research samples: trans men, trans women, mixed trans adults, trans boys, trans girls, mixed trans children, mixed trans adult and children, transgender, genderqueer/nonbinary, “disorders of sex development” (“DSD”), cisgender, trans and “DSD,” “DSD” and cisgender, LGBT, artifacts, statutes, policies, fictitious (film and literature).

There were no discernable patterns garnered from the metadata. Nearly 45% of articles referred to GD as a diagnostic term for trans people and/or intersex people, and the remaining articles assumed GD in their work without citing the *DSM-5*. The three major narrative themes we will be drawing on in this article are: (1) Distress and Diagnoses, (2) Types of People with Distress, (3) Popular Discourses and Distress.

## Distress and Diagnoses

### Foregrounding “distress”

The number of articles that offered a description of GD from the *DSM-5* was 49% (Supplementary Table S2). This left 51% of the articles (see Supplementary Table S1: references with *) that conceptualized GD in ways other than as a diagnostic concept, which we comment on further hereunder. Just >46% of the articles that referenced the *DSM-5*, however, did not fully outline the diagnosis as it appears. These articles truncated the description, which resulted in an interpretation of the diagnosis that foregrounded arguments of “distress.” For instance, most of the articles suggested that the distress could be alleviated after the process of assessment and surgical interventions and that multidisciplinary teams were the teams to diagnose and support any medical interventions necessary. Only eight articles (just >2%, see Supplementary Table S2: references with *) included a full description, such as Barry et al.s’,^44^ who noted that the incongruence between gender identity and assigned gender does not interfere with all trans people's lives; they are completely contented living just the way they are or may desire some forms of medical intervention, and it is only for a subset of transgender people, however, the incongruence results in GD, that is, a feeling of stress and discomfort with one's assigned gender.

Six of the articles including a full depiction of the diagnosis were reviews in psychology, one article was from law, and one article was from a group of clinicians working in a U.K. gender clinic who stated, “We have previously questioned the value of distress as a core criterion for diagnosis in DSM classification as it is a very general use of language, not specifically pathognomonic for any mental or physical illness, disorder or condition, and rather open to a wide range of interpretation as to what constitutes marked or clinically significant distress.”^45(p. 165)^

### Depathologizes gender nonconformity

Quite a number of articles (14.5%: Supplementary Table S3) endorse the *DSM-5* work group's claims that the new diagnostic criterion serves to depathologize gender nonconformity. These ranged from ambitious claims, such as “DSM-5 also recognizes gender identity in individuals as an option and not a stigmatized mental illness,”^46(p. 422)^ to lesser but no less problematic assertions, such as “the term ‘gender dysphoria’ has replaced gender identity disorder and this change in terminology removes the ‘pathology’ from being transgender, which is not a mental health condition.”^47(p. 792)^ In addition, this and 5.7% (Supplementary Table S4) of other articles highlight that transgender identities are no longer considered a sign of pathology, but an aspect of human diversity. Semantically this may be true; however, they offer no indication of how or why the change in name removes pathology from being transgender.

The GD diagnosis within the *DSM-5* emphasizes “incongruence” between assigned gender and experienced gender, and sets out diagnostic criterion such as the desire to be treated as one's experienced gender within society, or a conviction that one's feelings or reactions are typical of people with the same gender identity. Wishing to be treated as a woman or man within society, or believing that one's feelings are typical of one's status as a woman or man, would plainly not be considered diagnostic indicators for cisgender people. As such, the diagnosis continues to assume that the gender identities of trans and intersex people should be regarded differently to those cisgender people. As Aiken^48^ argues, although removing the word “disorder” from the diagnosis, the new entry in the *DSM-5* still maintains the notion that transgender- or gender-nonconforming individuals are mentally ill.

### Diagnosis and stigma

Many of the articles (12%: Supplementary Table S5) articulated that trans people and to a much lesser extent intersex people were often discriminated against, suffered much abuse, and occupied a stigmatized position in society. Campbell et al.s’^43^ review highlighted that much of the distress associated with trans and intersex people appears to reflect social stigmatization from negative reactions from family, friends, and society. Although the majority of these articles acknowledged that psychiatric diagnoses can stigmatize populations, 3.1% (Supplementary Table S6) of the articles (including some that also suggested widespread discrimination and stigma) claimed that the change in nomenclature from GID to GD would reduce this stigma. Little evidence was provided for this claim, and it seems questionable to conclude that changing diagnostic terminology in a clinical manual would substantially address discrimination and stigma within wider society.

### Not all trans and intersex people suffer from GD

The claimed destigmatizing and depathologizing effects in the literature were derived from the assertion in the *DSM-5* that it is the distress and not the gender diversity that is diagnosable. Despite this, many articles omitted the fact that not all trans and intersex people experience GD. Arguments in the literature required the distress factor to conform to the distress merits medical intervention ideal. As such, articles only partially appropriate the *DSM-5*'s diagnosis to support claims that the diagnosis ensures clinical interventions by a multidisciplinary team and that health insurance payments would unlikely be forthcoming without a diagnosis (7.9%: Supplementary Table S7). A narrative about access to clinical interventions and health insurance payments on the basis of not suffering distress or impairment to social functioning would indeed seem odd.

To apply a diagnosis to all who require medical interventions and who do not suffer dysphoria or impairment to social functioning with a diagnosis of GD is also medically questionable. This is especially so for those who, before being offered medical interventions, must demonstrate GD. In these articles then, the psychiatric diagnosis functions as a gatekeeping check in socioeconomic climates, such as in the United States, that requires a diagnosis for medical interventions and/or the granting of legal gender recognition, leaving the question whether a psychiatric diagnosis' validity should be about pathology or should it be used to aid any secondary gains, such as citizenship rights.^49^

The post-transition specifier diagnosis was explained in just >2% (Supplementary Table S8) of the articles, as a stage after some treatment and when the trans person requires ongoing treatment, but is “no longer gender dysphoric”—no literature mentioned the post-transition specifier in relation to intersex people. The primary purpose of this post-transition specifier is to facilitate medical insurance payments for ongoing interventions that may continue to occur after distress has been resolved—for example, to allow for ongoing hormone therapy.^2^ Some of these articles acknowledged this.

The post-transition specifier was modeled on the concept of “remission”^50^; however, this does not make much sense for nondysphoric or nonimpaired individuals who may have transitioned. If someone is nondysphoric/nonimpaired originally, then logically no remission of dysphoria can take place. If the post-transition specifier of GD is applied to nondysphoric/nonimpaired people, this would result in a false positive diagnosis, which has further implications for our analysis about the prevalence of GD hereunder.

Because of the confusing diagnosis, understandably, there has been a host of conceptual issues conflated in the articles analyzed. Implicitly and explicitly presenting GD in this way results in a narrative of distress that may not always be warranted and thus the validity of the diagnosis of GD is brought into question on grounds of the illogicality of remission, when the *DSM* states that not all people suffer distress.

## Types of People with Distress

### GD prevalence

The rationale behind the GD diagnosis in the *DSM-5* was to offer clinicians a common language and orientate them toward accurate and dependable diagnoses. Epidemiologically, some (7.8%: Supplementary Table S9) commented on the prevalence of GD. However, prevalence statistics were typically derived from the number of people referred to gender clinics, implicitly suggesting that everyone who is referred meets the diagnostic criterion for GD, which may not be the case (and, conversely, also assuming that everyone experiencing GD accesses gender identity services).

There were numerous terminology conflations (transgender/transsexual/GID/GD), making it difficult to clearly understand the number of people actually suffering with GD because the classification systems used have varied over time. Many authors have used GD inconsistently within the articles on prevalence, for example, conflating GID, GD, and transsexualism (52%: Supplementary Table S9 references *). The diagnostic criteria for GID, GD, and transsexualism may overlap, but are not identical. It, therefore, cannot be assumed that prevalence figures for one set of diagnostic criteria can be simply applied to another and consequently cannot underpin any validity claims.

### Etiology

Similarly, a few articles (3.5%: Supplementary Table S10) discussed questions about what “caused” GD in biomedical terms. However, in almost all these cases, they appeared to be looking at possible causes of incongruence between assigned gender and experienced gender, and not seeking the causes of distress. For instance, in a meta-synthesis, Arcelus et al.^51(p. 809)^ searched for “Transsexualism, gender identity disorder or gender dysphoria [which] must be diagnosed according to DSM-III, DSM-IIIR, DSM-IV, DSM-IV-TR, DSM-V [*sic*], ICD-9, ICD-10, or Benjamin criteria” to be included in their study. Other articles (3.5%: Supplementary Table S11) argued that there were no discernable biological evidence of GD.

The *DSM-5* explicitly states that diverse gender identities are not in themselves disordered, and disorder solely relates to distress. Yet the articles discussing GD's “etiology” in terms of seeking biomedical causes for a particular “nonconforming gender identity” clearly presume that such identities themselves require medical explanation. Moreover, if the intention of research is to understand links between biology and gender identity, it is methodologically weak to attempt to do so by looking solely at clinically referred populations for whom gender diversity is causing distress or impairment. Moreover, the *DSM-5* clearly points to the existence of gender diverse populations who do not experience distress or impairment, prevalence studies then do not contribute to the validity of any tools determining GD.

### Presumption of “dysphoria” in a given population group

GD was used as a descriptor for a group of people, for example, those who had a history of GD, those undergoing transitioning medical procedure, or those who had been referred to a gender clinic, who may or may not have been diagnosed with GD. A large majority of articles did not make it clear whether “clinically significant distress” or social impairment had been established, or even assessed by a clinician or the researcher. Other international diagnostic criteria in common use in the articles, such as the *ICD-10*'s^52^ gender identity categories, do not include distress as a criterion. It, therefore, cannot be automatically assumed that patients accessing gender services or undergoing transition medical procedures necessarily experienced distress, especially in jurisdictions wherein the *ICD-10* is used in preference to the *DSM*.

In addition, the post-transition specifier within the *DSM-5* also allows for the possibility that some people may have a prior diagnosis of GD, but not currently be experiencing distress. Inexact use of the term GD, therefore, carries a danger of presuming ubiquitous distress in a population group wherein levels of distress are in fact not known as we demonstrate hereunder.

### Research samples and gender dysphoric patients

Within research populations (14% of articles, see Supplementary Table S12), the timing or location of the fieldwork, the age of the participants, and in the case of intersex people before 2013 were diagnosable under different criteria and made it highly unlikely that the individuals researched had been diagnosed with GD. The timeframe required to make it into print, for some of the articles, would account for some of this discrepancy, however, not for all of them.

As already noted, only 2.5% of articles used the postspecifier concept. Surprisingly, however, was that the post-transition specifier was not used in all the articles describing the long-term follow-up of research participants who had previously received medical interventions related to their gender identity. Such participants were described using terminology such as “gender dysphoric” without reference to the possibility that their dysphoria might have been alleviated through the interventions they had received. There is much evidence to suggest that various medical interventions are desired and required by trans people.^53,54^ In most cases, this is to alleviate the pressure of living a gender that they were not assigned at birth.^55,56^ The empirical basis for treatment options and long-term outcomes with regard to intersex patient preferences are insufficient.^57^ Nonetheless, continuing to describe all trans and intersex people as “gender dysphoric” long after treatment is inconsistent with the claims about the suitability and effectiveness of treatments received by trans (and intersex) people that were made within the very same articles.

## Popular Discourses Within the Literature

### Hierarchies of dysphoria

Several articles (4.6%: Supplementary Table S13) described transsexualism as an extreme form of GD, or stated that only people with extreme GD would seek medical transition. This characterization that transsexualism is the most extreme form of GD and associated with a desire to medically transition reifies and reintroduces hierarchies of need between trans, intersex, and gender diverse people. The language about “extreme” GD in the reviewed articles assumes that there is direct correlation between severity of distress and bodily intervention choices an individual will make. No evidence was provided in the articles to support this implied correlation nor does gradated levels of GD appear in current diagnostic texts.

Older texts (notably Benjamin^58^) do directly posit a hierarchy in which there are “true” transsexuals, who have the most extreme form of gender incongruence, and are presumed to most likely to be “successful” transitioners. Medical services have often been designed with mental health professionals serving mainly as “gatekeepers” to gender-affirming hormone therapy and surgery.^59(p. 441)^ The gatekeeping is predicated on the assumption that it is clinically important to distinguish between these “true” transsexuals and individuals with other forms of gender diversity, who are perceived to be more likely to regret interventions. However, the concept of “true transsexualism” is no longer present, and the current standards of care emphasize the role of professionals to be supportive rather than gatekeepers. Articles that imply a hierarchy of GD are, therefore, replicating outdated notions of the role of medical services.

The persistence of this idea of hierarchy potentially derives, in part, from the apparent crossover between three different texts used in a majority of the medical/clinical articles: the *ICD-10*, *DSM-IV*, and *DSM-5*. In the *ICD-10*, “transsexualism” is a subtype of GID (although it is not explicitly termed as the most extreme subtype), but GD is not a diagnosis in the manual. In the *DSM-IV'*s GID diagnosis, there is no subtype of transsexualism. The *DSM-5*'s definition of GD is not synonymous with *ICD-10* definitions. In, particular the *ICD-10* does not require that distress be present. Transsexualism is, therefore, neither a subtype of GD nor a direct analogue in any diagnostic manual even though a number of articles seen in Supplementary Table S12 deem it to be the same phenomena.

### GD and “disorders of sex development”

A somewhat separate narrative relates to people with intersex conditions (often termed DSD in the literature), although this is disputed term.^60^ Some articles explicitly stated that GD and intersex were distinct. However, the new diagnostic criterion allows psychiatrists to diagnose GD for people with intersex conditions comparably with trans people.

Much of the medical literature regarding GD and intersex conditions in this review focused upon decisions around birth assignation, and the possibility that “gender dysphoria” might occur later. The application of the term GD for those with variations of sex development^61^ serves a euphemizing function, disguising the responsibility of medical professionals and parents who take decisions to socially and surgically assign young children to a gender category and, in many cases, utilize medical interventions to “normalize” the morphology of the body. Most authors did not discuss ethical questions about assigning intersex people a gender or using clinical interventions, despite an acknowledgment that subsequent distress with the gender assignation was a possible outcome.

Diagnosing GD as a consequence of clinical childhood interventions with intersex people raises fundamental questions about the original assignment by doctors and/or parents. However, none of the articles referred to this. The fact that some intersex people who later experience clinically significant distress or impairment related to their gender in these cases renders assignment at birth practices problematic.

Furthermore, the popular narrative of GD is that it is distress arising from incongruence between people's experienced gender identity and their “biological” gender. However, intersex people, by definition, have biological characteristics that do not entirely align with classifications of a male or female gender. How then can congruence or incongruence between biology and identity be correctly defined within these (9.5%: Supplementary Table S14) articles? The lack of evidence underpinning the diagnosis of GD in the *DSM-5* in relation to intersex people is obvious here.

Few texts centered on intersex people's own feelings or experiences regarding their identity. Rather, their GD feelings or experiences were discussed within a biomedical paradigm and, thus, cannot contribute to the content validity of GD as distress or impairment in social functioning.

## Conclusion

We have drawn together some of the main narrative characterizations of GD from 387 articles and highlighted three key themes: distress and diagnosis, types of people, and popular discourse. Although the stated intention of the *DSM-5* was to focus diagnosis upon distress and impairment, we have demonstrated that this is inconsistently applied within the literature. Some articles ignore the reference to distress entirely, whereas others imply distress where it may never have been present, or may have subsided.

Frequent changes of terminology, and crossover between medicalized and identity terms, appear to have contributed to conflation and confusion to the extent that GD is sometimes referred to as a specific diagnosis; sometimes as a phenomenological experience of distress; and sometimes as a personal characteristic within individuals. This frequent diverse usage of “gender dysphoria,” and the application of the concept to populations who may neither meet diagnostic criteria nor experience distress may potentially undermine the stated intention of the APA that the new diagnosis of GD would reduce pathologization.

We have also pointed to the replication within the academic literature of popular discourses of GD, rooted in normative assumptions about which forms of medical intervention with regard to bodily sex development should be treated as uncontroversial and inherently justified, and which should be seen as requiring special justification. The *DSM-5*'s redefinition of GD does not appear to have resolved concerns with regard to the scientificity or content validity of the diagnosis related to medical transition or intersex experiences.

Appropriate terminology with regard to gender diversity has been a longstanding and frequently fraught debate. There has been broad support for the APA's explicit recognition that gender diversity should not be universally pathologized and that the focus of health and social care professionals should be upon reducing distress and impairment to facilitate gender diverse people's equitable participation within society. However, these positive aims are most likely to be fulfilled if concepts of “gender dysphoria” are clearly defined by clinicians, researchers, theorists, and publishers, underpinned by robust evidence, critical analysis, and peer review that is rooted within the lived experience of gender diverse people and communities.

## Supplementary Material

Supplemental data

Supplemental data

Supplemental data

Supplemental data

Supplemental data

Supplemental data

Supplemental data

Supplemental data

Supplemental data

Supplemental data

Supplemental data

Supplemental data

Supplemental data

Supplemental data

## Abbreviations Used

APA: American Psychiatric Association
DSM: *Diagnostic and Statistical Manual of Mental Disorders*
DSD: disorders of sex development
GD: gender dysphoria
GID: gender identity disorder
UGDS: Utrecht gender dysphoria scale
