# Supplementary material for: What Is Gender Dysphoria? A Critical Systematic Narrative Review
Source: Transgend Health. 2018 Nov 1;3(1):159–69. doi: 10.1089/trgh.2018.0014 (PMC6225591; doi:10.1089/trgh.2018.0014)
Supplement: Supplemental data [file Supp_Table1.docx]

Supplementary Table S1. Analyzed Articles

| - Abbott TB. The Trans/Romance Dilemma in Transamerica and Other Films. The Journal of American Culture 2013;36(1):32-41. - Abel BS. Hormone Treatment of Children and Adolescents with Gender Dysphoria: An Ethical Analysis. Hastings Center Report 2014;44(s4):S23-S7. - Acton LA. Overturning in re Gardiner: Ending transgender discrimination in Kansas. Family Law Quarterly, 2014; v. 48. - Ahlam A, Al Sinawi H, Al Alawi M. Gender dysphoria in an Omani female. International Journal of Nutrition, Pharmacology, Neurological Diseases 2016;6(2):97-9. - Ahmad S, Barrett J, Beaini AY, et al. Gender dysphoria services: a guide for general practitioners and other healthcare staff. Sexual and Relationship Therapy 2013;28(3):172-85. - Aiken J. Promoting an Integrated Approach to Ensuring Access to Gender Incongruent Health Care. Berkeley Journal of Gender, Law & Justice 2016;Winter. - Aitken M, Steensma TD, Blanchard R, et al. Evidence for an Altered Sex Ratio in Clinic-Referred Adolescents with Gender Dysphoria. The J Sex Med 2015;12(3):756-63. - Aitken M, VanderLaan DP, Wasserman L, et al. Self-Harm and Suicidality in Children Referred for Gender Dysphoria. J Am Acad Child Adolesc Psychiatry 2016;55(6):513-20. - Alegría CA. Gender nonconforming and transgender children/youth: Family, community, and implications for practice. Journal of the American Association of Nurse Practitioners 2016;28(10):521-7. - Alfaro-Martínez JJ. Historical figures at the office of Endocrinology. Endocrinología y Nutrición (English Edition) 2014;61(7):382-8. - Amaral RC, Inacio M, Brito VN, et al. Quality of life of patients with 46,XX and 46,XY disorders of sex development. Clin Endocrinol (Oxf) 2015;82(2):159-64. - Amend B, Seibold J, Toomey P, et al. Surgical Reconstruction for Male-to-Female Sex Reassignment. Eur Urol 2013;64(1):141-9. - Anderson JA. Pitch Elevation in Trangendered Patients: Anterior Glottic Web Formation Assisted by Temporary Injection Augmentation. J Voice 2014;28(6):816-21. - Anderson S. Disorders of Sexual Differentiation: Ethical Considerations Surrounding Early Cosmetic Genital Surgery. J Pediatr Nurs 2015;41(4):176-86. - Antoni C. Service Delivery and the Challenges of Providing Service to People Who Are Transgender. SIG 3 Perspectives on Voice and Voice Disorders 2015;25(2):59-65. - Arcelus J, Bouman WP, Van Den Noortgate W, et al. Systematic review and meta-analysis of prevalence studies in transsexualism. Eur Psychiatry 2015;30(6):807-15. - Arcelus J, Claes L, Witcomb GL, et al. Risk Factors for Non-Suicidal Self-Injury Among Trans Youth. J Sex Med 2016;13(3):402-12. - Armand H MA. Pubertal Suppression and Professional Obligations: May a Pediatric Endocrinologist Refuse to Treat an Adolescent With Gender Dysphoria? Am J Bioeth 2014;14(1):43-6. - Atienza-Macías E. Some Legal Thoughts on Transsexuality in the Healthcare System After the New Edition of the Diagnostic and Statistical Manual of Mental Disorders (DSM). Sexuality & Culture 2015;19(3):574-6. - Atkinson SR, Russell D. Gender dysphoria. Aust Fam Physician 2015;44(11):792-6. - Auer MK, Fuss J, Stalla GK, Athanasoulia AP. Twenty years of endocrinologic treatment in transsexualism: analyzing the role of chromosomal analysis and hormonal profiling in the diagnostic work-up. Fertil Steril 2013;100(4):1103-10. - Auer MK, Hellweg R, Briken P, et al. Serum brain-derived neurotrophic factor (BDNF) is not regulated by testosterone in transmen. Biology of Sex Differences 2016;7(1):1-6. - Austriaco NPG. The Specification of Sex/Gender in the Human Species: A Thomistic Analysis. New Blackfriars 2013;94(1054):701-15. - Aydin D, Buk LJ, Partoft S, et al. Transgender Surgery in Denmark From 1994 to 2015: 20-Year Follow-Up Study. J Sex Med 2016;13(4):720-5. - Azul D. On the Varied and Complex Factors Affecting Gender Diverse People's Vocal Situations: Implications for Clinical Practice. SIG 3 Perspectives on Voice and Voice Disorders 2015;25(2):75-86. - Azul D. Transmasculine people's vocal situations: a critical review of gender-related discourses and empirical data. Int J Lang Commun Disord 2015;50(1):31-47. - Bachmann GA, Mussman B. The aging population: Imperative to uncouple sex and gender to establish “gender equal” health care. Maturitas 2015;80(4):421-5. - Bailey L, Ellis SJ, McNeil J. Suicide risk in the UK trans population and the role of gender transition in decreasing suicidal ideation and suicide attempt. Mental Health Review Journal 2014;19(4):209-20. - Bailey M. Transgender Workplace Discrimination in the Age of Gender Dysphoria and EDNA. Law & Psychology Review 2014;38:193-210. - Bajpai M. "Bird-Wing" abdominal phalloplasty: A novel surgical technique for penile reconstruction. Journal of Indian Association of Pediatric Surgeons 2013;18(2):49-52. - Baral SD, Poteat T, Strömdahl S, et al. Worldwide burden of HIV in transgender women: a systematic review and meta-analysis. Lancet Infect Dis 2013;13(3):214-22. - Baril A. Needing to Acquire a Physical Impairment/Disability: (Re)Thinking the Connections between Trans and Disability Studies through Transability. Hypatia 2015;30(1):30-48. - Barišić J, Milosavljević M, Duišin D, et al. Assessment of Self-Perception of Transsexual Persons: Pilot Study of 15 Patients. The Scientific World Journal, 2014; v. 2014. - Barry KM, Farrell B, Levi JL, Vanguri N. A Bare Desire to Harm: Transgender People and the Equal Protection Clause. Boston College Law Review 2016;57(507):507-82. - Bartolucci C, Gómez-Gil E, Salamero M, et al. Sexual Quality of Life in Gender-Dysphoric Adults before Genital Sex Reassignment Surgery. J Sex Med 2015;12(1):180-8. - Bauer GR, Scheim AI, Pyne J, et al. Intervenable factors associated with suicide risk in transgender persons: a respondent driven sampling study in Ontario, Canada. BMC Public Health 2015;15(1):1-15. - Becerra-Fernández A, Pérez-López G, Menacho Román M, et al. Prevalence of hyperandrogenism and polycystic ovary syndrome in female to male transsexuals. Endocrinología y Nutrición (English Edition) 2014;61(7):351-8. - Becker I, Nieder TO, Cerwenka S, et al. Body Image in Young Gender Dysphoric Adults: A European Multi-Center Study. Arch Sex Behav 2016;45(3):559-74. - Beek TF, Cohen-Kettenis PT, Kreukels BPC. Gender incongruence/gender dysphoria and its classification history. Int Rev Psychiatry 2016;28(1):5-12. - Beek TF, Kreukels BPC, Cohen-Kettenis PT, Steensma TD. Partial Treatment Requests and Underlying Motives of Applicants for Gender Affirming Interventions. J Sex Med 2015;12(11):2201-5. - Bell F. Children with gender dysphoria and the jurisdiction of the Family Court. University of New South Wales Law Journal 2015;38(2):426-54. - Bendlin S. Gender Dysphoria in the Jailhouse: A Constitutional Right to Hormone Therapy? Cleveland State Law Review 2013;61(4):957-82. - Berenbaum SA, Meyer-Bahlburg HFL. Gender development and sexuality in disorders of sex development. Horm Metab Res 2015;47(5):361-6. - Bermúdez de la Vega JA, Fernández-Cancio M, Bernal S, Audí L. Complete Androgen Insensitivity Syndrome Associated with Male Gender Identity or Female Precocious Puberty in the Same Family. Sexual Development 2015;9(2):75-9. - Bockting W, Coleman E, Deutsch MB, et al. Adult development and quality of life of transgender and gender nonconforming people. Current Opinion in Endocrinology, Diabetes and Obesity 2016;23(2):188-97. - Bockting WO. Vulnerability and Resilience Among Gender-Nonconforming Children and Adolescents: Mental Health Professionals Have a Key Role to Play. J Am Acad Child Adolesc Psychiatry 2016;55(6):441-3. - Bockting WO, Miner MH, Swinburne Romine RE, et al. Stigma, Mental Health, and Resilience in an Online Sample of the US Transgender Population. Am J Public Health 2013;103(5):943-51. - Bodoin EM, Byrd CT, Adler RK. The Clinical Profile of the Male-to-Female Transgender Person of the 21st Century. Contemporary Issues in Communication Science & Disorders 2014;41:39-54. - Bonifacio HJ, Rosenthal SM. Gender Variance and Dysphoria in Children and Adolescents. Pediatr Clin North Am 2015;62(4):1001-16. - Boroughs MS, Bedoya CA, O'Cleirigh C, Safren SA. Toward Defining, Measuring, and Evaluating LGBT Cultural Competence for Psychologists. Clinical Psychology: Science and Practice 2015;22(2):151-71. - Bosse JD, Nesteby JA, Randall CE. Integrating Sexual Minority Health Issues into a Health Assessment Class. J Prof Nurs 2015;31(6):498-507. - Bouman WP. The Good Practice Guidelines for the Assessment and Treatment of Adults with Gender Dysphoria in the United Kingdom. Sexual and Relationship Therapy 2014;29(2):149-51. - Bouman WP, Davey A, Meyer C, et al. Predictors of psychological well-being among treatment seeking transgender individuals. Sexual and Relationship Therapy 2016:1-17. - Bouman WP, de Vries ALC, T’Sjoen G. Gender Dysphoria and Gender Incongruence: An evolving inter-disciplinary field. Int Rev Psychiatry 2016;28(1):1-4. - Bouman WP, Richards C. Diagnostic and Treatment Issues for People with Gender Dysphoria in the United Kingdom. Sexual and Relationship Therapy 2013;28(3):165-71. - Bouman WP, Richards C, Addinall RM, et al. Yes and yes again: are standards of care which require two referrals for genital reconstructive surgery ethical? Sexual and Relationship Therapy 2014;29(4):377-89. - Brown GR. Breast Cancer in Transgender Veterans: A Ten-Case Series. LGBT Health 2015;2(1):77-80. - Brown GR, Jones KT. Incidence of breast cancer in a cohort of 5,135 transgender veterans. Breast Cancer Res Treat 2015;149(1):191-8. - Brown T. Dangers of Overboard Transgender Legislation, Case Law, and Policy in Education: California's AB 1266 Dismisses Concerns about Student Safety and Privacy, The. Brigham Young University Education & Law Journal 2014;2014(2):287-319. - Bui HN, Schagen SEE, Klink DT, et al. Salivary testosterone in female-to-male transgender adolescents during treatment with intra-muscular injectable testosterone esters. Steroids 2013;78(1):91-5. - Buncamper ME, Honselaar JS, Bouman M-B, et al. Aesthetic and Functional Outcomes of Neovaginoplasty Using Penile Skin in Male-to-Female Transsexuals. J Sex Med 2015;12(7):1626-34. - Burke SM, Cohen-Kettenis PT, Veltman DJ, et al. Hypothalamic Response to the Chemo-Signal Androstadienone in Gender Dysphoric Children and Adolescents. Frontiers in Endocrinology: Frontiers Media S.A., 2014; v. 5. - Burke SM, Kreukels BPC, Cohen-Kettenis PT, et al. Male-typical visuospatial functioning in gynephilic girls with gender dysphoria - organizational and activational effects of testosterone. J Psychiatry Neurosci 2016;41(6):395-404. - Burke SM, Menks WM, Cohen-Kettenis PT, et al. Click-Evoked Otoacoustic Emissions in Children and Adolescents with Gender Identity Disorder. Arch Sex Behav 2014;43(8):1515-23. - Busari AO. Bolstering Self-Esteem as Intervention Technique in the Management of Symptoms of Gender Identity Disorder among Adolescents. Gender & Behaviour 2013;11(2):5535-45. - Callens N, De Cuypere G, Van Hoecke E, et al. Sexual Quality of Life after Hormonal and Surgical Treatment, Including Phalloplasty, in Men with Micropenis: A Review. J Sex Med 2013;10(12):2890-903. - Campbell MM, Artz L, Stein DJ. Sexual disorders in DSM-5 and ICD-11: a conceptual framework. Current Opinion in Psychiatry 2015;28(6):435-9. - Capetillo-Ventura NC, Jalil-Pérez SI, Motilla-Negrete K. Gender dysphoria: An overview. Medicina Universitaria 2015;17(66):53-8. - Capitán L, Simon D, Kaye K, Tenorio T. Facial Feminization Surgery: The Forehead. Surgical Techniques and Analysis of Results. Plast Reconstr Surg 2014;134(4):609-19. - Cardoso da Silva D, Schwarz K, Fontanari AMV, et al. WHOQOL-100 Before and After Sex Reassignment Surgery in Brazilian Male-to-Female Transsexual Individuals. J S Med 2016;13(6):988-93. - Carrera MV, Lameiras M, DePalma R, Casas RR. Pathologizing gender identity: An analysis of Spanish law and the regulation of gender recognition. Journal of Gender Studies 2013;22(2):206-20. - Carrera-Fernández MV, Lameiras-Fernández M, Rodríguez-Castro Y, Vallejo-Medina P. Spanish Adolescents’ Attitudes toward Transpeople: Proposal and Validation of a Short Form of the Genderism and Transphobia Scale. J Sex Res 2014;51(6):654-66. - Castañeda C. Developing gender: The medical treatment of transgender young people. Soc Sci Med 2015;143:262-70. - Castellini G. Language of self-definition in the disorders of identity. Organo Ufficiale della Società Italiana di Psicopatologia 2016;22(1):39-47. - Castellini G, Lelli L, Ricca V, Maggi M. Sexuality in eating disorders patients: etiological factors, sexual dysfunction and identity issues. A systematic review. Hormone Molecular Biology and Clinical Investigation 2016;25(2):71-90. - Cerwenka S, Nieder TO, Cohen-Kettenis P, et al. Sexual Behavior of Gender-Dysphoric Individuals Before Gender-Confirming Interventions: A European Multicenter Study. J Sex Marital Ther 2014;40(5):457-71. - Chen M, Fuqua J, Eugster EA. Characteristics of Referrals for Gender Dysphoria Over a 13-Year Period. J Adolesc Health 2016;58(3):369-71. - Chuang J, Vallerie A, Breech L, et al. Complexities of gender assignment in 17β-hydroxysteroid dehydrogenase type 3 deficiency: is there a role for early orchiectomy? International Journal of Pediatric Endocrinology 2013;2013(1):1-6. - Church HA, O’Shea D, Lucey JV. Parent–child relationships in gender identity disorder. Ir J Med Sc 2014;183(2):277-81. - Ciocca G, Limoncin E, Cellerino A, et al. Gender Identity Rather Than Sexual Orientation Impacts on Facial Preferences. J Sexl Med 2014;11(10):2500-7. - Claes L, Bouman WP, Witcomb G, et al. Non-Suicidal Self-Injury in Trans People: Associations with Psychological Symptoms, Victimization, Interpersonal Functioning, and Perceived Social Support. J Sexl Med 2015;12(1):168-79. - Cohen-Kettenis PT, Klink D. Adolescents with gender dysphoria. Best Pract Res Clin Endocrinol Metab 2015;29(3):485-95. - Colizzi M, Costa R, Scaramuzzi F, et al. Concomitant psychiatric problems and hormonal treatment induced metabolic syndrome in gender dysphoria individuals: A 2 year follow-up study. J Psychosom Res 2015;78(4):399-406. - Colizzi M, Costa R, Todarello O. Dissociative symptoms in individuals with gender dysphoria: Is the elevated prevalence real? Psychiatry Res 2015;226(1):173-80. - Collazo A, Austin A, Craig SL. Facilitating Transition Among Transgender Clients: Components of Effective Clinical Practice. Clinical Social Work Journal 2013;41(3):228-37. - Collin L, Reisner SL, Tangpricha V, Goodman M. Prevalence of Transgender Depends on the “Case” Definition: A Systematic Review. The J Sex Med 2016;13(4):613-26. - Corbett K, Dimen M, Goldner V, Harris A. Talking Sex, Talking Gender—A Roundtable. Studies in Gender and Sexuality 2014;15(4):295-317. - Costa MM, Lau E, Rodrigues P, et al. Gender Dysphoria in a Genetic Female with Non-Classical Congenital Adrenal Hyperplasia. Arch Sex Behav 2016:1-4. - Costa R, Dunsford M, Skagerberg E, et al. Psychological Support, Puberty Suppression, and Psychosocial Functioning in Adolescents with Gender Dysphoria. J Sexl Med 2015;12(11):2206-14. - Costantino A, Cerpolini S, Alvisi S, et al. A Prospective Study on Sexual Function and Mood in Female-to-Male Transsexuals During Testosterone Administration and After Sex Reassignment Surgery. J Sex Marital Ther 2013;39(4):321-36. - Couturier J, Pindiprolu B, Findlay S, Johnson N. Anorexia nervosa and gender dysphoria in two adolescents. International Journal of Eating Disorders 2015;48(1):151-5. - Daley A, Mulé NJ. LGBTQs and the DSM-5: A Critical Queer Response. J Homosex 2014;61(9):1288-312. - Daly TTW. Gender Dysphoria and the Ethics of Transsexual (i.e. Gender Reassignment) Surgery. Ethics & Medicine: An International Journal of Bioethics 2016;32(1):39-53. - Daniolos PT, Telingator CJ. Engendering Identity. J Am Acad Child Adolesc Psychiatry 2013;52(12):1245-7. - Dargie E, Blair KL, Pukall CF, Coyle SM. Somewhere under the rainbow: Exploring the identities and experiences of trans persons. The Canadian Journal of Human Sexuality 2014;23(2):60-74. - Davey A, Arcelus J, Meyer C, Bouman WP. Self-injury among trans individuals and matched controls: prevalence and associated factors. Health and Social Care in the Community 2016;24(4):485-94. - Davey A, Bouman WP, Meyer C, Arcelus J. Interpersonal Functioning Among Treatment-Seeking Trans Individuals. J Clin Psychol 2015;71(12):1173-85. - Davey A, Meyer C, Arcelus J, Bouman WP. Social Support and Psychological Well-Being in Gender Dysphoria: A Comparison of Patients With Matched Controls. J Sex Med 2014;11(12):2976-85. - Davies A, Bouman WP, Richards C, et al. Patient satisfaction with gender identity clinic services in the United Kingdom. Sexual and Relationship Therapy 2013;28(4):400-18. - Davy Z. The DSM-5 and the Politics of Diagnosing Transpeople. Arch Sex Behav 2015;44(5):1165-76. - de Vries ALC, McGuire JK, Steensma TD, et al. Young Adult Psychological Outcome After Puberty Suppression and Gender Reassignment. Pediatrics 2014;134(4):696-704. - de Vries ALC, Steensma TD, Cohen-Kettenis PT, et al. Poor peer relations predict parent- and self-reported behavioral and emotional problems of adolescents with gender dysphoria: a cross-national, cross-clinic comparative analysis. Eur Child Adolesc Psychiatry 2016;25(6):579-88. - DeFeo J. Understanding Sexual, Paraphilic, and Gender Dysphoria Disorders in DSM-5. J Child Sex Abus 2015;24(2):210-5. - Delahunt JW, Denison HJ, Kennedy J, et al. Specialist services for management of individuals identifying as transgender in New Zealand. N Z Med J 2016;129(1434). - Dèttore D, Ristori J, Antonelli P, et al. Gender dysphoria in adolescents: the need for a shared assessment protocol and proposal of the AGIR protocol. Journal of Psychopathology 2015;21(2):152-8. - Deuster D, Matulat P, Knief A, et al. Voice deepening under testosterone treatment in female-to-male gender dysphoric individuals. Eur Arch Otorhinolaryngol 2016;273(4):959-65. - Dhejne C, Van Vlerken R, Heylens G, Arcelus J. Mental health and gender dysphoria: A review of the literature. Int Rev Psychiatry 2016;28(1):44-57. - Di Ceglie D, Skagerberg E, Baron-Cohen S, Auyeung B, 16 (6). Empathising and systemising in adolescents with gender dysphoria. Opticon 1826 2014;16(6):1-8. - Diamond M, Garland J. Evidence regarding cosmetic and medically unnecessary surgery on infants. Journal of Pediatric Urology 2014;10(1):2-6. - Djordjevic ML, Salgado CJ, Bizic M, Kuehhas FE. Gender Dysphoria: The Role of Sex Reassignment Surgery. The Scientific World Journal 2014;2014:2. - Drescher J. Controversies in Gender Diagnoses. LGBT Health 2013;1(1):10-4. - Drescher J. Queer diagnoses revisited: The past and future of homosexuality and gender diagnoses in DSM and ICD. Int Rev Psychiatry 2015:1-10. - Drescher J. Gender Policing in the Clinical Setting: Discussion of Sandra Silverman’s “The Colonized Mind: Gender, Trauma, and Mentalization”. Psychoanalytic Dialogues 2015;25(1):67-76. - Drescher J, Pula J. Ethical Issues Raised by the Treatment of Gender-Variant Prepubescent Children. Hastings Cent Rep 2014;44(s4):S17-S22. - Duisin D, Barisic J, Batinic B. Request for sex-reconversion surgery - case report. Eur Psychiatry 2013;28, Supplement 1:1. - Eapen V, Črnčec R. DSM 5 and child psychiatric disorders: What is new? What has changed? Asian Journal of Psychiatry 2014;11:114-8. - Ehsanzadeh P, Raza S, Haq Z. A New Perspective on Gender Dysphoria and Repetitive Sex Reassignment Surgeries: A Case Report. The Primary Care Companion for CNS Disorders 2014;16(2):PCC.13l01608. - Ekenze SO, Adiri CO, Igwilo IO, Onumaegbu OO. Virilized External Genitalia in Young Girls: Clinical Characteristics and Management Challenges in a Low-Resource Setting. Journal of Pediatric and Adolescent Gynecology 2014;27(1):6-9. - Ellena W, Neel H. Gender disorders in learning disability – a systematic review. Tizard Learning Disability Review 2014;19(4):158-65. - Ellis SJ, Bailey L, McNeil J. Trans People's Experiences of Mental Health and Gender Identity Services: A UK Study. Journal of Gay & Lesbian Mental Health 2015;19(1):4-20. - Erasmus J, Bagga H, Harte F. Assessing patient satisfaction with a multidisciplinary gender dysphoria clinic in Melbourne. Australas Psychiatry 2015;23(2):158-62. - Eren E, Edgünlü T, Asut E, Karakaş Çelik S. Homozygous Ala65Pro Mutation with V89L Polymorphism in SRD5A2 Deficiency. Journal of Clinical Pediatric Endocrinology 2016;8(2):218-23. - Esteva de Antonio I, Gómez-Gil E, Group aG. Coordination of healthcare for transsexual persons: a multidisciplinary approach. Current Opinion in Endocrinology, Diabetes and Obesity 2013;20(6):585-91. - Ettner R, Wylie K. Psychological and social adjustment in older transsexual people. Maturitas 2013;74(3):226-9. - Eugster EA. The Use of Gonadotropin-Releasing Hormone Analogs beyond Precocious Puberty. J Pediatr 2015;167(2):481-5. - Ewan LA, Middleman AB, Feldmann J. Treatment of anorexia nervosa in the context of transsexuality: A case report. International Journal of Eating Disorders 2014;47(1):112-5. - Fabris B, Bernardi S, Trombetta C. Cross-sex hormone therapy for gender dysphoria. J Endocrinol Invest 2015;38(3):269-82. - Faccio E, Bordin E, Cipolletta S. Transsexual parenthood and new role assumptions. Cult Health Sex 2013;15(9):1055-70. - Feldman J, Spencer K. Gender dysphoria in a 39-year-old man. Can Med Assoc J 2014;186(1):49. - Fernández R, Esteva I, Gómez-Gil E, et al. Association Study of ERβ, AR, and CYP19A1 Genes and MtF Transsexualism. J Sex Med 2014;11(12):2986-94. - Feusner JD, Dervisic J, Kosidou K, et al. Female-to-Male Transsexual Individuals Demonstrate Different Own Body Identification. Arch Sex Behav 2016;45(3):525-36. - Firth MT. Childhood abuse, depressive vulnerability and gender dysphoria: Part 2. Counselling & Psychotherapy Research 2015;15(2):98-108 11p. - Fisher AD, Castellini G, Bandini E, et al. Cross-Sex Hormonal Treatment and Body Uneasiness in Individuals with Gender Dysphoria. J Sex Med 2014;11(3):709-19. - Fisher AD, Castellini G, Casale H, et al. Hypersexuality, Paraphilic Behaviors, and Gender Dysphoria in Individuals with Klinefelter's Syndrome. J Sex Med 2015;12(12):2413-24. - Fitzgibbons RP. Transsexual attractions and sexual reassignment surgery: Risks and potential risks. Linacre Q 2015;82(4):337-50. - Fontanari A-MV, Andreazza T, Costa ÂB, et al. Serum concentrations of brain-derived neurotrophic factor in patients with gender identity disorder. J Psychiatr Res 2013;47(10):1546-8. - Furnham A, Sen R. Lay Theories of Gender Identity Disorder. J Homosex 2013;60(10):1434-49. - Fuss J, Auer MK, Briken P. Gender dysphoria in children and adolescents: a review of recent research. Current Opinion in Psychiatry 2015;28(6):430-4. - Fuss J, Biedermann SV, Stalla GK, Auer MK. On the quest for a biomechanism of transsexualism: Is there a role for BDNF? J Psychiatr Res 2013;47(12):2015-7. - Fuss J, Hellweg R, Van Caenegem E, et al. Cross-sex hormone treatment in male-to-female transsexual persons reduces serum brain-derived neurotrophic factor (BDNF). Eur Neuropsychopharmacol 2015;25(1):95-9. - Garcia N. Starting with the Man in the Mirror: Transsexual Prisoners and Transitional Surgeries Following Kosilek v. Spencer. Am J Law Med 2014;40(4):442-63. - Gardiner JK. Masculinity's Interior: Men, Transmen, and Theories of Masculinity. The Journal of Men's Studies 2013;21(2):112-26. - Gava G, Cerpolini S, Martelli V, et al. Cyproterone acetate vs leuprolide acetate in combination with transdermal oestradiol in transwomen: a comparison of safety and effectiveness. Clin Endocrinol (Oxf) 2016;85(2):239-46. - Giami A, Beaubatie E. Gender Identification and Sex Reassignment Surgery in the Trans Population: A Survey Study in France. Arch Sex Behav 2014;43(8):1491-501. - Ginsberg BA, Calderon M, Seminara NM, Day D. A potential role for the dermatologist in the physical transformation of transgender people: A survey of attitudes and practices within the transgender community. Journal of the American Academy of Dermatology 2016;74(2):303-8. - Gómez-Gil E, Gutiérrez F, Cañizares S, et al. Temperament and character in transsexuals. Psychiatry Res 2013;210(3):969-74. - Gómez-Gil E, Zubiaurre-Elorza L, Esteva de Antonio I, et al. Determinants of quality of life in Spanish transsexuals attending a gender unit before genital sex reassignment surgery. Qual Life Res 2014;23(2):669-76. - González R, Ludwikowski BM. Gender Dysphoria in 46,XX Persons with Adrenogenital Syndrome Raised as Females: An Addendum. Frontiers in Pediatrics 2014;2:140. - Gonzalez-Salzberg DA. The Accepted Transsexual and the Absent Transgender: A Queer Reading of the Regulation of Sex/Gender by the European Court of Human Rights, The. American University International Law Review 2013;29(4):797-829. - Gooren LJ, Giltay EJ. Men and women, so different, so similar: observations from cross-sex hormone treatment of transsexual subjects. Andrologia 2014;46(5):570-5. - Gooren LJ, Kreukels B, Lapauw B, Giltay EJ. (Patho)physiology of cross-sex hormone administration to transsexual people: the potential impact of male-female genetic differences. Andrologia 2015;47(1):5-19. - Gordetsky J, Joseph DB. Cloacal Exstrophy: A History of Gender Reassignment. Urology 2015;86(6):1087-9. - Görtz DP, Commons ML. The stage-value model: Implications for the changing standards of care. Int J Law Psychiatry 2015;42–43:135-43. - Gray SAO, Sweeney KK, Randazzo R, Levitt HM. “Am I Doing the Right Thing?”: Pathways to Parenting a Gender Variant Child. Fam Process 2016;55(1):123-38. - Gregor C, Davidson S, Hingley-Jones H. The experience of gender dysphoria for pre-pubescent children and their families: a review of the literature. Child & Family Social Work 2014;21(3):339-46. - Gregor C, Hingley-Jones H, Davidson S. Understanding the Experience of Parents of Pre-pubescent Children with Gender Identity Issues. Child and Adolescent Social Work Journal 2015;32(3):237-46. - Gridley SJ, Crouch JM, Evans Y, et al. Youth and Caregiver Perspectives on Barriers to Gender-Affirming Health Care for Transgender Youth. J Adolesc Health 2016; 59(3):254-261. - Guillamon A, Junque C, Gómez-Gil E. A Review of the Status of Brain Structure Research in Transsexualism. Arch Sex Behav 2016;45(7):1-34. - Güldenring A. A critical view of transgender health care in Germany: Psychopathologizing gender identity – Symptom of ‘disordered’ psychiatric/psychological diagnostics? Int Rev Psychiatry 2015;27(5):427-34. - Guzman-Parra J, Paulino-Matos P, de Diego-Otero Y, et al. Substance Use and Social Anxiety in Transsexual Individuals. Journal of Dual Diagnosis 2014;10(3):162-7. - Handy AB, Wassersug RJ, Ketter JTJ, Johnson TW. The sexual side of castration narratives: Fiction written by and for eunuchs and eunuch ''wannabes''. Canadian Journal of Human Sexuality 2015;24(2):151-9. - Hardy TLD, Boliek CA, Wells K, Rieger JM. The ICF and Male-to-Female Transsexual Communication. International Journal of Transgenderism 2013;14(4):196-208. - Hatami M, Ayvazi S. Investigating of Personality Characteristics (Extroversion-introversion) and Early Maladaptive Schemas (EMS) in Males and Females with Gender Identity Disorder (GID). Procedia - Social and Behavioral Sciences 2013;84:1474-80. - Henry M. A One-Inch Mound of Flesh: Troubling Queer Identity in Hedwig and the Angry Inch. The Journal of American Culture 2016;39(1):64-77. - Hermann M, Thorstenson A. A Rare Case of Male‐to‐Eunuch Gender Dysphoria. Sex Med 2015;3(4):331-3. - Hewitt J, Zacharin M. Hormone replacement in disorders of sex development: Current thinking. Best Pract Res Clin Endocrinol - Metab 2015;29(3):437-47. - Heylens G, Elaut E, Verschelden G, Cuypere GD. Transgender Persons Applying for Euthanasia in Belgium: A Case Report and Implications for Assessment and Treatment. Journal of Psychiatry 2016;19(1):347. - Heylens G, Verroken C, De Cock S, et al. Effects of Different Steps in Gender Reassignment Therapy on Psychopathology: A Prospective Study of Persons with a Gender Identity Disorder. J Sex Med 2014;11(1):119-26. - Higuchi T, Holmdahl G, Kaefer M, et al. International Consultation on Urological Diseases: Congenital Anomalies of the Genitalia in Adolescence. Urology 2016;94:288-310. - Hill T. Transgender Military Inmates' Legal and Constitutional Rights to Medical Care in Prisons: Serious Medical Need versus Military Necessity. Vermont Law Review 2014;39(2):411-59. - Hoekzema E, Schagen SEE, Kreukels BPC, et al. Regional volumes and spatial volumetric distribution of gray matter in the gender dysphoric brain. Psychoneuroendocrinology 2015;55:59-71. - Hoffman B. An Overview of Depression among Transgender Women. Depression Research and Treatment 2014;2014:1-9. - Holt V, Skagerberg E, Dunsford M. Young people with features of gender dysphoria: Demographics and associated difficulties. Clinical Child Psychology and Psychiatry 2016;21(1):108-18. - Horbach SER, Bouman M-B, Smit JM, et al. Outcome of Vaginoplasty in Male-to-Female Transgenders: A Systematic Review of Surgical Techniques. J Sex Med 2015;12(6):1499-512. - Hsu KJ, Rosenthal AM, Bailey JM. The Psychometric Structure of Items Assessing Autogynephilia. Arch Sex Behav 2015;44(5):1301-12. - Ishak MSBH, Haneef SSS. Sex Reassignment Technology: The Dilemma of Transsexuals in Islam and Christianity. J Relig Health 2014;53(2):520-37. - Jacobs LA, Rachlin K, Erickson-Schroth L, Janssen A. Gender Dysphoria and Co-Occurring Autism Spectrum Disorders: Review, Case Examples, and Treatment Considerations. LGBT Health 2014;1(4):277-82. - Jafari F. Transsexuality under Surveillance in Iran: Clerical Control of Khomeini’s Fatwas. Journal of Middle East Women's Studies 2014;10(2):31-51. - Joel D, Tarrasch R, Berman Z, et al. Queering gender: studying gender identity in ‘normative’ individuals. Psychology & Sexuality 2014;5(4):291-321. - Johnson L, Shipherd J, Walton HM. The psychologist’s role in transgender-specific care with U.S. veterans. Psychological Services 2016;13(1):69-77. - Jones BA, Haycraft E, Murjan S, Arcelus J. Body dissatisfaction and disordered eating in trans people: A systematic review of the literature. Int Rev Psychiatry 2016;28(1):81-94. - Judge C, O'Donovan C, Callaghan G, et al. Gender dysphoria - prevalence and co-morbidities in an Irish adult population. Frontiers in Endocrinology, 2014;5(87)1-5. - Junger J, Habel U, Bröhr S, et al. More than Just Two Sexes: The Neural Correlates of Voice Gender Perception in Gender Dysphoria. PLoS ONE 2014;9(11):1-12. - Jürgensen M, Kleinemeier E, Lux A, et al. Psychosexual Development in Adolescents and Adults with Disorders of Sex Development—Results from the German Clinical Evaluation Study. J Sex Med 2013;10(11):2703-14. - Jürgensen M, Lux A, Wien SB, et al. Health-related quality of life in children with disorders of sex development (DSD). Eur J Pediatr 2014;173(7):893-903. - Kalra G, Shah N. The Cultural, Psychiatric, and Sexuality Aspects of Hijras in India. International Journal of Transgenderism 2013;14(4):171-81. - Kalra G, Tandon A, Sathyanarayana Rao TS. Sexual disorders in Asians: A review. Asian Journal of Psychiatry 2014;7:80-2. - Kaltiala-Heino R, Sumia M, Työläjärvi M, Lindberg N. Two years of gender identity service for minors: overrepresentation of natal girls with severe problems in adolescent development. Child and Adolescent Psychiatry and Mental Health 2015;1(9):1-9. - Kanhere M, Fuqua J, Rink R, et al. Psychosexual development and quality of life outcomes in females with congenital adrenal hyperplasia. International Journal of Pediatric Endocrinology 2015;2015(1):1-9. - Kauth MR, Shipherd JC, Lindsay J, et al. Access to Care for Transgender Veterans in the Veterans Health Administration: 2006–2013. Am J Public Health 2014;104(S4):S532-S4. - Kelly F. Australian children living with gender dysphoria: does the Family Court have a role to play? J Law Med 2014;22(1):105-20. - Kelso T. Still Trapped in the U.S. Media’s Closet: Representations of Gender-Variant, Pre-Adolescent Children. J Homosex 2015;62(8):1058-97. - Kern L, Edmonds P, Perrin EC, Stein MT. An 8-year-old Biological Female Who Identifies Herself as a Boy: Perspectives in Primary Care and from a Parent. Journal of Developmental & Behavioral Pediatrics 2014;35(4):301-3. - Keuroghlian AS, Reisner SL, White JM, Weiss RD. Substance use and treatment of substance use disorders in a community sample of transgender adults. Drug Alcohol Depend 2015;152:139-46. - Khattab A, Yau M, Qamar A, et al. Long term outcomes in 46, XX adult patients with congenital adrenal hyperplasia reared as males. J Steroid Biochem Mol Biol 2016 online first. - Kipnis K. Ethics, Morality, and Pediatric Gender Dysphoria. Am J Bioeth 2014;14(1):50-1. - Kirkovski M, Enticott PG, Fitzgerald PB. A Review of the Role of Female Gender in Autism Spectrum Disorders. Journal of Autism and Developmental Disorders 2013;43(11):2584-603. - Kon AA. Transgender Children and Adolescents. Am J Bioeth 2014;14(1):48-50. - Kranz GS, Hahn A, Baldinger P, et al. Cerebral serotonin transporter asymmetry in females, males and male-to-female transsexuals measured by PET in vivo. Brain Structure and Function 2014;219(1):171-83. - Kranz GS, Wadsak W, Kaufmann U, et al. High-Dose Testosterone Treatment Increases Serotonin Transporter Binding in Transgender People. Biol Psychiatry 2015;78(8):525-33. - Kraus C. Classifying Intersex in DSM-5: Critical Reflections on Gender Dysphoria. Arch Sex Behav 2015;44(5):1147-63. - Kreukels BPC, Guillamon A. Neuroimaging studies in people with gender incongruence. Int Rev Psychiatry 2016;28(1):120-8. - Kristensen ZE, Broome MR. Autistic Traits in an Internet Sample of Gender Variant UK Adults. International Journal of Transgenderism 2015;16(4):234-45. - Kukreti P, Kandpal M, Jiloha RC. Mistaken gender identity in non-classical congenital adrenal hyperplasia. Indian Journal of Psychiatry 2014;56(2):182-4. - Langer SJ. Our Body Project: From Mourning to Creating the Transgender Body. International Journal of Transgenderism 2014;15(2):66-75. - Lee RC. Forced Sterilization and Mandatory Divorce: How A Majority of Council of Europe Member States’ Laws Regarding Gender Identity Violate the Internationally and Regionally Established Human Rights of Trans* People. Berkeley Journal of International Law, 2015;33:114-152. - Leibowitz S, de Vries ALC. Gender dysphoria in adolescence. Int Rev Psychiatry 2016;28(1):21-35. - Lekarev O, Lin-Su K, Vogiatzi MG. Infertility and Reproductive Function in Patients with Congenital Adrenal Hyperplasia: Pathophysiology, Advances in Management, and Recent Outcomes. Endocrinology and Metabolism Clinics of North America 2015;44(4):705-22. - Lemaire M, Thomazeau B, Bonnet-Brilhault F. Gender Identity Disorder and Autism Spectrum Disorder in a 23-Year-Old Female. Arch Sex Behav 2014;43(2):395-8. - Lenning E, Buist CL. Social, psychological and economic challenges faced by transgender individuals and their significant others: gaining insight through personal narratives. Cult Health Sex 2013;15(1):44-57. - Lev AI. Gender Dysphoria: Two Steps Forward, One Step Back. Clinical Social Work Journal 2013;41(3):288-96. - Levin D. Does changing gender make children happier? Arch Dis Child 2016;101(5):460. - Levine DA, Braverman PK, Adelman WP, et al. Office-Based Care for Lesbian, Gay, Bisexual, Transgender, and Questioning Youth. Pediatrics 2013;132(1):198-203. - Levman J, Takahashi E. Multivariate analyses applied to fetal, neonatal and pediatric MRI of neurodevelopmental disorders. Neuroimage 2015;9:532-44. - Li F, Rendall D, Vasey PL, et al. The development of sex/gender-specific /s/ and its relationship to gender identity in children and adolescents. Journal of Phonetics 2016;57:59-70. - Lin C-S, Ku H-L, Chao H-T, et al. Neural Network of Body Representation Differs between Transsexuals and Cissexuals. PLoS ONE 2014;9(1):e85914. - Loverro G, Di Naro E, Caringella AM, et al. Prevalence of human papillomavirus infection in a clinic sample of transsexuals in Italy. Sex Transm Infect 2016;92(1):67-9. - Lynch MM, Khandheria MM, Meyer WJ. Retrospective Study of the Management of Childhood and Adolescent Gender Identity Disorder Using Medroxyprogesterone Acetate. International Journal of Transgenderism 2015;16(4):201-8. - Maccio EM, Ferguson KM. Services to LGBTQ runaway and homeless youth: Gaps and recommendations. Children and Youth Services Review 2016;63:47-57. - Mackenzie C. Embodied agents, narrative selves. Philosophical Explorations 2014;17(2):154-71. - Mackie V. Genders and Genetics: The Legal and Medical Regulation of Family Forms in Contemporary Japan. Australian Journal of Asian Law 2013;14(1):1-18. - Maddera JC. Batson in Transition: Prohibiting Peremptory Challenges on the Basis of Gender Identity or Expression. Columbia Law Review 2016;116(1):195-235. - Majumder A, Sanyal D. Outcome and preferences in female-to-male subjects with gender dysphoria: Experience from Eastern India. Indian Journal of Endocrinology & Metabolism 2016;20(3):308-11. - Mandal E, Jakubowski T. Masculinity, femininity, self-appeal, strategies of self-presentation and styles of interpersonal functioning in transsexual women. Archives of Psychiatry & Psychotherapy 2015;17(3):5-13. - Marshall E, Claes L, Bouman WP, et al. Non-suicidal self-injury and suicidality in trans people: A systematic review of the literature. Int Rev Psychiatry 2016;28(1):58-69. - Marshall J, Cooper M, Rudnick A. Gender Dysphoria and Dementia: A Case Report. Journal of Gay & Lesbian Mental Health 2015;19(1):112-Masroor AS. Gender Identity Disorder is Not Simply Two in One. The International Medical Journal of Malaysia 2013;12(2):83-5. - Matza AR, Sloan CA, Kauth MR. Quality LGBT Health Education: A Review of Key Reports and Webinars. Clinical Psychology: Science & Practice 2015;22(2):127-44. - Mazaheri Meybodi A, Hajebi A, Ghanbari Jolfaei A. Psychiatric Axis I Comorbidities among Patients with Gender Dysphoria. Psychiatry Journal 2014;2014:1-5. - McCann E, Sharek D. Mental Health Needs of People Who Identify as Transgender: A Review of the Literature. Arch Psychiatr Nurs 2016;30(2):280-5. - McCracken KA, Fallat ME. Transition from pediatric to adult surgery care for patients with disorders of sexual development. Semin Pediatr Surg 2015;24(2):88-92. - McDonald E. Intersex people in Aotearoa New Zealand: The challenges for law and social policy: Part I: Critiquing gender normalising surgery. Victoria University. Wellington Law Review 2015;46:705-24. - McKitrick J. A dispositional account of gender. Philosophical Studies 2015;172(10):2575-89. - Mepham N, Bouman WP, Arcelus J, et al. People with Gender Dysphoria Who Self-Prescribe Cross-Sex Hormones: Prevalence, Sources, and Side Effects Knowledge. J Sex Med 2014;11(12):2995-3001. - Meriggiola MC, Gava G. Endocrine care of transpeople part I. A review of cross-sex hormonal treatments, outcomes and adverse effects in transmen. Clin Endocrinol (Oxf) 2015;83(5):597-606. - Merryfeather L, Bruce A. The Invisibility of Gender Diversity: Understanding Transgender and Transsexuality in Nursing Literature. Nurs Forum 2014;49(2):110-23. - Meyer-Bahlburg HFL. Psychoendocrinology of Congenital Adrenal Hyperplasia. In: Hammer MI, New O, Alan L, et al., eds. Genetic Steroid Disorders. San Diego: Academic Press, 2014:pp. 285-300. - Milrod C. How Young Is Too Young: Ethical Concerns in Genital Surgery of the Transgender MTF Adolescent. J Sex Med 2014;11(2):338-46. - Mishali Y. Feminine trouble: The removal of femininity from feminist/lesbian/queer esthetics, imagery, and conceptualization. Women's Studies International Forum 2014;44:55-68. - Moleiro C, Pinto N. Sexual Orientation and Gender Identity: Review of concepts, controversies and their relation to psychopathology classification systems. Frontiers in Psychology 2015 Available <https://www.frontiersin.org/articles/10.3389/fpsyg.2015.01511/full> Accessed April 12, 2018. - Morrison SD, Perez MG, Carter CK, Crane CN. Pre- and Post-Operative Care With Associated Intra-Operative Techniques for Phalloplasty In Female-to-Male Patients. Urol Nurs 2015;35(3):134-8. - Motmans J, Ponnet K, De Cuypere G. Sociodemographic Characteristics of Trans Persons in Belgium: A Secondary Data Analysis of Medical, State, and Social Data. Arch Sex Behav 2015;44(5):1289-99. - Mouriquand PDE, Gorduza DB, Gay C-L, et al. Surgery in disorders of sex development (DSD) with a gender issue: If (why), when, and how? Journal of Pediatric Urology 2016;12(3):139-49. - Munson B, Crocker L, Pierrehumbert JB, et al. Gender typicality in children's speech: A comparison of boys with and without gender identity disorder. Journal of the Acoustical Society of America 2015;137(4):1995-2003. - Nakachi Y, Iseki M, Yokoo T, et al. Gene Expression Profile of the Neonatal Female Mouse Brain After Administration of Testosterone Propionate. J Sex Med 2015;12(4):887-96. - Nelson JL. Medicine and Making Sense of Queer Lives. Hastings Center Report 2014;44(s4):S12-S6. - Ni H-C, Gau SS-F. Co-occurrence of attention-deficit hyperactivity disorder symptoms with other psychopathology in young adults: parenting style as a moderator. Compr Psychiatry 2015;57:85-96. - Nieder TO, Elaut E, Richards C, Dekker A. Sexual orientation of trans adults is not linked to outcome of transition-related health care, but worth asking. Int Rev Psychiatry 2016;28(1):103-11. - Nygren U, Nordenskjöld A, Arver S, Södersten M. Effects on Voice Fundamental Frequency and Satisfaction with Voice in Trans Men during Testosterone Treatment—A Longitudinal Study. J Voice 2016;30(6):766 e23- e34. - Obedin-Maliver J, Makadon HJ. Transgender men and pregnancy. Obstetric Medicine: The Medicine of Pregnancy 2016;9(1):4-8. - Öcal G, Berberoğlu M, Sıklar Z, et al. Clinical Review of 95 Patients with 46,XX Disorders of Sex Development Based on the New Chicago Classification. Journal of Pediatric and Adolescent Gynecology 2015;28(1):6-11. - Olson J, Schrager SM, Belzer M, et al. Baseline Physiologic and Psychosocial Characteristics of Transgender Youth Seeking Care for Gender Dysphoria. J Adolesc Health 2015;57(4):374-80. - Olson KR, Durwood L, DeMeules M, McLaughlin KA. Mental Health of Transgender Children Who Are Supported in Their Identities. Pediatrics 2016;137(3):e 20153223. - Osborne CS, Lawrence AA. Male Prison Inmates With Gender Dysphoria: When Is Sex Reassignment Surgery Appropriate? Arch Sex Behav 2016:1-15. - Parco JE, Levy DA, Spears SR. Transgender Military Personnel in the Post-DADT Repeal Era: A Phenomenological Study. Armed Forces & Society 2014;41(2): 221–42. - Pariser JJ, Cohn JA, Gottlieb LJ, Bales GT. Buccal Mucosal Graft Urethroplasty for the Treatment of Urethral Stricture in the Neophallus. Urology 2015;85(4):927-31. - Parkinson J. Gender dysphoria in Asperger’s syndrome: a caution. Australas Psychiatry 2014;22(1):84-5. - Pasterski V, Gilligan L, Curtis R. Traits of Autism Spectrum Disorders in Adults with Gender Dysphoria. Arch Sex Behav 2014;43(2):387-93. - Pasterski V, Zucker KJ, Hindmarsh PC, et al. Increased Cross-Gender Identification Independent of Gender Role Behavior in Girls with Congenital Adrenal Hyperplasia: Results from a Standardized Assessment of 4- to 11-Year-Old Children. Arch Sex Behav 2015;44(5):1363-75. - Petricevic L, Kaufmann U, Domig KJ, et al. Rectal Lactobacillus Species and Their Influence on the Vaginal Microflora: A Model of Male-to-Female Transsexual Women. J Sex Med 2014;11(11):2738-43. - Prunas A, Vitelli R, Agnello F, et al. Defensive functioning in MtF and FtM transsexuals. Compr Psychiatry 2014;55(4):966-71. - Quam K. Unfinished Business of Repealing Don't Ask, Don't Tell: The Military's Unconstitutional Ban on Transgender Individuals. Utah Law Review 2015(3):721-41. - Rabito Alcón MF, Rodríguez Molina JM. Body image in persons with gender dysphoria. Medwave, 2015; Available <http://www.medwave.cl/link.cgi/English/Original/Research/6150?ver=sindiseno> Accessed April 12, 2018. - Rabito-Alcón MF, Rodríguez-Molina JM. Satisfaction with life and psychological well-being in people with gender dysphoria. Actas Esp Psiquiatr 2016;44(2):47-54. - Raffaini M, Magri AS, Agostini T. Full Facial Feminization Surgery: Patient Satisfaction Assessment Based on 180 Procedures Involving 33 Consecutive Patients. Plast Reconstr Surg 2016;137(2):438-48. - Raigosa M, Avvedimento S, Yoon TS, et al. Male-to-Female Genital Reassignment Surgery: A Retrospective Review of Surgical Technique and Complications in 60 Patients. J Sex Med 2015;12(8):1837-45. - Rajkumar RP. Gender Identity Disorder and Schizophrenia: Neurodevelopmental Disorders with Common Causal Mechanisms? Schizophrenia Research and Treatment 2014;2014:1-8. - Rathi A, Bhatia MS. Management challenges in a case of gender identity disorder. Industrial Psychiatry Journal 2014;23(2):157-9. - Reay B. The Transsexual Phenomenon: A Counter-History. Journal of Social History 2014;47(4):1042-71. - Reisner SL, Deutsch MB, Bhasin S, et al. Advancing methods for US transgender health research. Current Opinion in Endocrinology, Diabetes & Obesity 2016;23(2):198-207. - Reisner SL, Vetters R, Leclerc M, et al. Mental Health of Transgender Youth in Care at an Adolescent Urban Community Health Center: A Matched Retrospective Cohort Study. J Adolesc Health 2015;56(3):274-9. - Renukanthan A, Quinton R, Turner B, et al. Kallmann syndrome patient with gender dysphoria, multiple sclerosis, and thrombophilia. Endocrine 2015;50(2):496-503. - Rezwan N, Basit AA, Andrews H. Bilateral ureteric obstruction: an unusual complication of male-to-female gender reassignment surgery. BMJ Case Reports 2014. - Richards C, Bouman WP, Seal L, et al. Non-binary or genderqueer genders. International Review of Psychiatry 2016;28(1):95-102. - Riggs DW, Coleman K, Due C. Healthcare experiences of gender diverse Australians: a mixed-methods, self-report survey. BMC Public Health 2014;14(1):1-5. - Riggs DW, Due C. Support Experiences and Attitudes of Australian Parents of Gender Variant Children. Journal of Child and Family Studies 2015;24(7):1999-2007. - Rijn AB-v, Steensma TD, Kreukels BP, Cohen-Kettenis PT. Self-perception in a clinical sample of gender variant children. Clinical Child Psychology and Psychiatry 2013;18(3):464-74. - Riley EA, Sitharthan G, Clemson L, Diamond M. Recognising the needs of gender-variant children and their parents. Sex Education 2013;13(6):644-59. - Ristori J, Steensma TD. Gender dysphoria in childhood. Int Rev Psychiatry 2016;28(1):13-20. - Roberts AL, Rosario M, Slopen N, et al. Childhood Gender Nonconformity, Bullying Victimization, and Depressive Symptoms Across Adolescence and Early Adulthood: An 11-Year Longitudinal Study. Journal of the American Academy of Child & Adolescent Psychiatry 2013;52(2):143-52. - Roberts TK, Fantz CR. Barriers to quality health care for the transgender population. Clin Biochem 2014;47(10–11):983-7. - Roblin D, Barzilay J, Tolsma D, et al. A novel method for estimating transgender status using electronic medical records. Ann Epidemiol 2016;26(3):198-203. - Roen K, Pasterski V. Psychological research and intersex/DSD: recent developments and future directions. Psychology & Sexuality 2014;5(1):102-16. - Roerink S, Marsman D, van Bon A, Netea-Maier R. A Missed Diagnosis of Acromegaly During a Female-to-Male Gender Transition. Arch Sex Behav 2014;43(6):1199-201. - Romano D, Sedda A, Brugger P, Bottini G. Body ownership: When feeling and knowing diverge. Conscious Cogn 2015;34:140-8. - Rosenthal SM. Approach to the Patient: Transgender Youth: Endocrine Considerations. The Journal of Clinical Endocrinology & Metabolism 2014;99(12):4379-89. - Rosky CJ. No Promo Hetero: Children's Right to be Queer. Cardozo Law Review 2013;35(2):425-510. - Ross A. The Invisible Army: Why the Military Needs to Rescind Its Ban on Transgender Service Members. Southern California Interdisciplinary Law Journal 2014;23:185-216. - Rothkopf AC, John RM. Understanding Disorders of Sexual Development. J Pediatr Nurs 2014;29(5):e23-e34. - Rotondi NK, Bauer GR, Scanlon K, et al. Nonprescribed Hormone Use and Self-Performed Surgeries: “Do-It-Yourself” Transitions in Transgender Communities in Ontario, Canada. Am J Public Health 2013;103(10):1830-6. - Ruppin U, Pfäfflin F. Long-Term Follow-Up of Adults with Gender Identity Disorder. Arch Sex Behav 2015;44(5):1321-9. - Saketopoulou A. Mourning the Body as Bedrock: Developmental Considerations in Treating Transsexual Patients Analytically. Journal of the American Psychoanalytic Association 2014;62(5):773-806. - Salgado CJ, Nugent AG, Moody AM, et al. Immediate pedicled gracilis flap in radial forearm flap phalloplasty for transgender male patients to reduce urinary fistula. Journal of Plastic, Reconstructive & Aesthetic Surgery 2016;69(11):1551-7. - Sandor von Dresner K, Underwood LA, Suarez E, Franklin T. Providing Counseling for Transgendered Inmates: A Survey of Correctional Services. International Journal of Behavioral Consultation & Therapy 2013;7(4):38-44. - Sangganjanavanich VF, Headley JA. Facilitating Career Development Concerns of Gender Transitioning Individuals: Professional Standards and Competencies. The Career Development Quarterly 2013;61(4):354-66. - Sanyal D, Majumder A. Presentation of gender dysphoria: A perspective from Eastern India. Indian Journal of Endocrinology & Metabolism 2016;20(1):129-33. - Schmidt L, Levine R. Psychological Outcomes and Reproductive Issues Among Gender Dysphoric Individuals. Endocrinology and Metabolism Clinics of North America 2015;44(4):773-85. - Schneider C, Cerwenka S, Nieder TO, et al. Measuring Gender Dysphoria: A Multicenter Examination and Comparison of the Utrecht Gender Dysphoria Scale and the Gender Identity/Gender Dysphoria Questionnaire for Adolescents and Adults. Arch Sex Behav 2016;45(3):551-8. - Schneider F, Neuhaus N, Wistuba J, et al. Testicular Functions and Clinical Characterization of Patients with Gender Dysphoria (GD) Undergoing Sex Reassignment Surgery (SRS). J Sex Med 2015;12(11):2190-200. - Schneider F, Redmann K, Wistuba J, et al. Comparison of enzymatic digestion and mechanical dissociation of human testicular tissues. Fertil Steril 2015;104(2):302-11.e3. - Schueftan Gilban DL, Garcia Alves Junior PA, Ricarte Beserra IC. Health related quality of life of children and adolescents with congenital adrenal hyperplasia in Brazil. Health & Quality of Life Outcomes, 2014; Available [https://hqlo.biomedcentral.com/articles/10.1186/s12955-014-0107-2](https://hqlo.biomedcentral.com/articles/10.1186/s12955-014-0107-2%20%20) Accessed April 12, 2018. - Schwarz K, Fontanari AMV, Mueller A, et al. Transsexual Voice Questionnaire for Male-to-female Brazilian Transsexual People. J Voice 2017;31(1):e15-e20. - Schwarz K, Fontanari AMV, Mueller A, et al. Neural Correlates of Psychosis and Gender Dysphoria in an Adult Male. Arch Sex Behav 2016;45(3):761-5. - Schweizer K, Brunner F, Handford C, Richter-Appelt H. Gender experience and satisfaction with gender allocation in adults with diverse intersex conditions (divergences of sex development, DSD). Psychology & Sexuality 2014;5(1):56-82. - Seal LJ. Male hypogonadism and testosterone replacement therapy. Medicine 2013;41(10):557-61. - Seal LJ. A review of the physical and metabolic effects of cross-sex hormonal therapy in the treatment of gender dysphoria. Ann Clin Biochem 2016;53(1):10-20. - Selekman J, Diefenbeck C. The New DSM-5 and Its Impact on the Mental Health Care of Children. J Pediatr Nurs 2014;29(5):442-50. - Selvaggi G, Branemark R, Elander A, et al. Titanium-bone-anchored penile epithesis: Preoperative planning and immediate postoperative results. Journal of Plastic Surgery and Hand Surgery 2015;49(1):40-4. - Shabir I, Khurana ML, Joseph AA, et al. Phenotype, genotype and gender identity in a large cohort of patients from India with 5α-reductase 2 deficiency. Andrology 2015;3(6):1132-9. - Shimamura Y, Fujikawa A, Kubota K, et al. Perforation of the neovagina in a male-to-female transsexual: a case report. Journal of Medical Case Reports 2015;9(1):1-5. - Shires DA, Jaffee K. Factors Associated with Health Care Discrimination Experiences among a National Sample of Female-to-Male Transgender Individuals. Health Soc Work 2015;40(2):134-41. - Shumer DE, Nokoff NJ, Spack NP. Advances in the Care of Transgender Children and Adolescents. Adv Pediatr 2016;63(1):79-102. - Shumer DE, Reisner SL, Edwards-Leeper L, Tishelman A. Evaluation of Asperger Syndrome in Youth Presenting to a Gender Dysphoria Clinic. LGBT Health 2016;3(5):387-90. - Shumer DE, Roberts AL, Reisner SL, et al. Brief Report: Autistic Traits in Mothers and Children Associated with Child’s Gender Nonconformity. Journal of Autism and Developmental Disorders 2015;45(5):1489-94. - Shumer DE, Tishelman AC. The Role of Assent in the Treatment of Transgender Adolescents. International Journal of Transgenderism 2015;16(2):97-102. - Sigurjonsson H, Rinder J, Möllermark C, et al. Male to female gender reassignment surgery: Surgical outcomes of consecutive patients during 14 years. JPRAS Open 2015;6:69-73. - Simons L, Leibowitz S, Hidalgo M. Understanding Gender Variance in Children and Adolescents. Pediatr Ann 2014;43(6):e126-e31. - Skagerberg E, Davidson S, Carmichael P. Internalizing and Externalizing Behaviors in a Group of Young People with Gender Dysphoria. International Journal of Transgenderism 2013;14(3):105-12. - Skagerberg E, Di Ceglie D, Carmichael P. Brief Report: Autistic Features in Children and Adolescents with Gender Dysphoria. Journal Of Autism And Developmental Disorders 2015;45(8):2628-32. - Skagerberg E, Parkinson R, Carmichael P. Self-Harming Thoughts and Behaviors in a Group of Children and Adolescents with Gender Dysphoria. International Journal of Transgenderism 2013;14(2):86-92. - Smith A. Stories of 0s: Transgender Women, Monstrous Bodies, and the Canadian Prison System. Dalhousie Journal of Legal Studies 2014;23:149-71. - Smith ES, Junger J, Derntl B, Habel U. The transsexual brain – A review of findings on the neural basis of transsexualism. Neuroscience & Biobehavioral Reviews 2015;59:251-66. - Smith FD. Perioperative Care of the Transgender Patient. AORN J 2016;103(2):151-63. - Smith MK, Mathews B. Treatment for gender dysphoria in children: the new legal, ethical and clinical landscape. Med J Aust 2015;202(2):102-4. - Södersten M, Nygren U, Hertegård S, Dhejne C. Interdisciplinary Program in Sweden Related to Transgender Voice. SIG 3 Perspectives on Voice and Voice Disorders 2015;25(2):87-97. - Soleman RS, Schagen SEE, Veltman DJ, et al. Sex Differences in Verbal Fluency during Adolescence: A Functional Magnetic Resonance Imaging Study in Gender Dysphoric and Control Boys and Girls. J Sex Med 2013;10(8):1969-77 - Soleman RS, Staphorsius AS, Cohen-Kettenis PT, et al. Oestrogens are Not Related to Emotional Processing: a Study of Regional Brain Activity in Female-to-Male Transsexuals Under Gonadal Suppression. Cereb Cortex 2014;26(2):510–6. - Staphorsius AS, Kreukels BPC, Cohen-Kettenis PT, et al. Puberty suppression and executive functioning: An fMRI-study in adolescents with gender dysphoria. Psychoneuroendocrinology 2015;56:190-9. - Steensma TD, Kreukels BPC, de Vries ALC, Cohen-Kettenis PT. Gender identity development in adolescence. Horm Behav 2013;64(2):288-97. - Steensma TD, Zucker KJ, Kreukels BPC, et al. Behavioral and Emotional Problems on the Teacher’s Report Form: A Cross-National, Cross-Clinic Comparative Analysis of Gender Dysphoric Children and Adolescents. J Abnorm Child Psychol 2014;42(4):635-47. - Strandjord SE, Ng H, Rome ES. Effects of treating gender dysphoria and anorexia nervosa in a transgender adolescent: Lessons learned. International Journal of Eating Disorders 2015;48(7):942-5. - Strang JF, Kenworthy L, Dominska A, et al. Increased Gender Variance in Autism Spectrum Disorders and Attention Deficit Hyperactivity Disorder. Arch Sex Behav 2014;43(8):1525-33. - Stroumsa D. The State of Transgender Health Care: Policy, Law, and Medical Frameworks. Am J Public Health 2014;104(3):e31-e8. - Swann WB, Gómez Á, Vázquez A, et al. Fusion with the Cross-Gender Group Predicts Genital Sex Reassignment Surgery. Arch Sex Behav 2015;44(5):1313-8. - Tack LJW, Craen M, Dhondt K, et al. Consecutive lynestrenol and cross-sex hormone treatment in biological female adolescents with gender dysphoria: a retrospective analysis. Biology of Sex Differences 2016;7(1):1-11. - Tamar-Mattis A, Baratz A, Baratz Dalke K, Karkazis K. Emotionally and cognitively informed consent for clinical care for differences of sex development. Psychology & Sexuality 2014;5(1):44-55. - Taskinen S, Suominen JS, Mattila AK. Gender Identity and Sex Role in Patients Operated on for Bladder Exstrophy-Epispadias. The J Urol 2016;196(2):531-5. - Taylor LA. A Win for Transgender Employees: Chevron Deference for the EEOC'S Decision in Macy V. Holder. Journal of Law & Family Studies 2013;15(1):181-207. - Tell S. Intersex Management in the United States and Non-Western Cultures. Einstein Journal of Biology and Medicine 2016;30(1&2):6-15. - Terrier J-É, Courtois F, Ruffion A, Morel Journel N. Surgical Outcomes and Patients' Satisfaction with Suprapubic Phalloplasty. The J Sex Med 2014;11(1):288-98. - Thompson D. Commentary on “Gender disorders in learning disabilities – a systematic review”. Tizard Learning Disability Review 2014;19(4):166-9. - Thorn ED. Drop the Knife! Instituting policies of Nonsurgical Intervention for Intersex Infants. Family Court Review 2014;52(3):610-21. - Toffoletto S, Lanzenberger R, Gingnell M, et al. Emotional and cognitive functional imaging of estrogen and progesterone effects in the female human brain: A systematic review. Psychoneuroendocrinology 2014;50:28-52. - Toscano ME, Maynard E. Understanding the Link: “Homosexuality,” Gender Identity, and the DSM. Journal of LGBT Issues in Counseling 2014;8(3):248-63. - Tourchi A, Hoebeke P. Long-term outcome of male genital reconstruction in childhood. Journal of Pediatric Urology 2013;9(6, Part B):980-9. - Travis M. Accommodating Intersexuality in European Union Anti-Discrimination Law. European Law Journal 2015;21(2):180-99. - Trevor M, Boddy J. Transgenderism and Australian Social Work: A Literature Review. Australian Social Work 2013;66(4):555-70. - Turan Ş, Poyraz CA, Duran A. Prolonged anorexia nervosa associated with female-to-male gender dysphoria: A case report. Eat Behav 2015;18:54-6. - Turan Ş, Poyraz CA, Öcek Baş T, et al. Affective temperaments in subjects with female-to-male gender dysphoria. J Affect Disord 2015;176:61-4. - Van Caenegem E, Wierckx K, Elaut E, et al. Prevalence of Gender Nonconformity in Flanders, Belgium. Arch Sex Behav 2015;44(5):1281-7. - van de Grift TC, Cohen-Kettenis PT, Elaut E, et al. A network analysis of body satisfaction of people with gender dysphoria. Body Image 2016;17:184-90. - van de Grift TC, Cohen-Kettenis PT, Steensma TD, et al. Body Satisfaction and Physical Appearance in Gender Dysphoria. Arch Sex Behav 2016;45(3):575-85. - Van Der Miesen AIR, Hurley H, De Vries ALC. Gender dysphoria and autism spectrum disorder: A narrative review. Int Rev Psychiatry 2016;28(1):70-80. - van der Zwan YG, Callens N, van Kuppenveld J, et al. Long-Term Outcomes in Males with Disorders of Sex Development. J Urol 2013;190(3):1038-42. - van Schalkwyk GI, Klingensmith K, Volkmar FR. Gender identity and autism spectrum disorders. Yale J Biol Med 2015;88(1):81-3. - Vance SR, Ehrensaft D, Rosenthal SM. Psychological and Medical Care of Gender Nonconforming Youth. Pediatrics 2014;134(6):1184-92. - VanderLaan DP, Blanchard R, Wood H, et al. Birth weight and two possible types of maternal effects on male sexual orientation: A clinical study of children and adolescents referred to a Gender Identity Service. Dev Psychobiol 2015;57(1):25-34. - VanderLaan DP, Blanchard R, Wood H, Zucker KJ. Birth Order and Sibling Sex Ratio of Children and Adolescents Referred to a Gender Identity Service. PloS One 2014;9(3):e90257. - VanderLaan DP, Leef JH, Wood H, et al. Autism Spectrum Disorder Risk Factors and Autistic Traits in Gender Dysphoric Children. Journal of Autism and Developmental Disorders 2015;45(6):1742-50. - VanderLaan DP, Postema L, Wood H, et al. Do Children With Gender Dysphoria Have Intense/Obsessional Interests? J Sex Res 2015;52(2):213-9. - Vaughn M, Silver K, Murphy S, et al. Women with Disabilities Discuss Sexuality in San Francisco Focus Groups. Sexuality and Disability 2015;33(1):19-46. - Veale JF. Evidence Against a Typology: A Taxometric Analysis of the Sexuality of Male-to-Female Transsexuals. Arch Sex Behav 2014;43(6):1177-86. - Veale JF. Comments on Ethical Reporting and Interpretations of Findings in Hsu, Rosenthal, and Bailey’s (2014) “The Psychometric Structure of Items Assessing Autogynephilia”. Arch Sex Behav 2015;44(7):1743-6. - Veltman A, Chaimowitz G. Mental Health Care for People Who Identify as Lesbian, Gay, Bisexual, Transgender, and (or) Queer. Can J Psychiatry 2014;59(11):1-7. - Vitelli R. Adult Male-to-Female Transsexualism A Clinical Existential-Phenomenological Inquiry. Journal of Phenomenological Psychology 2015;46(1):33-68. - Vrouenraets LJJJ, Fredriks AM, Hannema SE, et al. Early Medical Treatment of Children and Adolescents With Gender Dysphoria: An Empirical Ethical Study. J Adolesc Health 2015;57(4):367-73. - Vrouenraets LJJJ, Fredriks AM, Hannema SE, et al. Perceptions of Sex, Gender, and Puberty Suppression: A Qualitative Analysis of Transgender Youth. Arch Sex Behav 2016:1-7. - Wallace R, Russell H. Attachment and Shame in Gender-Nonconforming Children and Their Families: Toward a Theoretical Framework for Evaluating Clinical Interventions. International Journal of Transgenderism 2013;14(3):113-26. - Wallace SA, Blough KL, Kondapalli LA. Fertility preservation in the transgender patient: expanding oncofertility care beyond cancer. Gynecol Endocrinol 2014;30(12):868-71. - Wang LC, Poppas DP. Surgical outcomes and complications of reconstructive surgery in the female congenital adrenal hyperplasia patient: What every endocrinologist should know. The Journal of Steroid Biochemistry and Molecular Biology 2017;165(Part A):137-44. - Wangjiraniran B, Selvaggi G, Chokrungvaranont P, et al. Male-to-female vaginoplasty: Preecha’s surgical technique. Journal of Plastic Surgery and Hand Surgery 2015;49(3):153-9. - Washburn M. Five Things Social Workers Should Know about the DSM-5. Social Work 2013;58(4):373-6. - White Hughto JM, Reisner SL, Pachankis JE. Transgender stigma and health: A critical review of stigma determinants, mechanisms, and interventions. Soc Sci Med 2015;147:222-31. - Whitehead JD, Whitehead EE. Transgender Lives: From Bewilderment to God's Extravagance. Pastoral Psychology 2014;63(2):171-85. - Wibowo E, Johnson TW, Wassersug RJ. Infertility, impotence, and emasculation - psychosocial contexts for abandoning reproduction. Asian J Androl 2016;18(3):403-8. - Wierckx K, Van de Peer F, Verhaeghe E, et al. Short- and Long-Term Clinical Skin Effects of Testosterone Treatment in Trans Men. The J Sex Med 2014;11(1):222-9. - Winograd W. The Wish to Be a Boy: Gender Dysphoria and Identity Confusion in a Self-Identified Transgender Adolescent. Psychoanalytic Social Work 2014;21(1-2):55-74. - Witcomb GL, Bouman WP, Brewin N, et al. Body Image Dissatisfaction and Eating-Related Psychopathology in Trans Individuals: A Matched Control Study. European Eating Disorders Review 2015;23(4):287-93. - Withers R. The seventh penis: towards effective psychoanalytic work with pre-surgical transsexuals. J Anal Psychol 2015;60(3):390-412. - Wood E, Halder N. Gender disorders in learning disability – a systematic review. Tizard Learning Disability Review 2014;19(4):158-65. - Wood H, Sasaki S, Bradley SJ, et al. Patterns of Referral to a Gender Identity Service for Children and Adolescents (1976–2011): Age, Sex Ratio, and Sexual Orientation. J Sex Marital Ther 2013;39(1):1-6. - Wylie K, Barrett J, Besser M, et al. Good Practice Guidelines for the Assessment and Treatment of Adults with Gender Dysphoria. Sexual and Relationship Therapy 2014;29(2):154-214. - Yang X, Wang L, Hao C, et al. Sex Partnership and Self-Efficacy Influence Depression in Chinese Transgender Women: A Cross-Sectional Study. PLoS ONE 2015;10(9):e0136975. - Zainuddin AA, Mahdy ZA. The Islamic Perspectives of Gender-Related Issues in the Management of Patients With Disorders of Sex Development. Arch Sex Behav 2016:online first:1-8. - Zucker KJ, Lawrence AA, Kreukels BPC. Gender Dysphoria in Adults. Annual Review of Clinical Psychology 2016;12(1):217-47. - Zucker KJ, Seto MC. Gender dysphoria and paraphilic sexual disorders. In: Thapar A, Pine DS, Leckman JF, et al., eds. Rutter's Child and Adolescent Psychiatry. Chichester: John Wiley & Sons, 2015:pp. 983-98. - Zuckerman JM, Smentkowski K, Gilbert D, et al. Penile Prosthesis Implantation in Patients with a History of Total Phallic Construction. J Sex Med 2015;12(12):2485-91. |
| --- |
