# Supplementary material for: What Is Gender Dysphoria? A Critical Systematic Narrative Review
Source: Transgend Health. 2018 Nov 1;3(1):159–69. doi: 10.1089/trgh.2018.0014 (PMC6225591; doi:10.1089/trgh.2018.0014)
Supplement: Supplemental data [file Supp_Table2.docx]

Supplementary Table S2. References citing DSM-5

| - Abel BS. Hormone Treatment of Children and Adolescents with Gender Dysphoria: An Ethical Analysis. Hastings Center Report 2014;44(s4):S23-S7. - Acton LA. Overturning in re gardiner: Ending transgender discrimination in kansas. Family Law Quarterly, 2014; v. 48. - Ahlam A, Al Sinawi H, Al Alawi M. Gender dysphoria in an Omani female. International Journal of Nutrition, Pharmacology, Neurological Diseases 2016;6(2):97-9. - *Ahmad S, Barrett J, Beaini AY, et al. Gender dysphoria services: a guide for general practitioners and other healthcare staff. Sexual and Relationship Therapy 2013;28(3):172-85. - Aiken J. Promoting an Integrated Approach to Ensuring Access to Gender Incongruent Health Care. Berkeley Journal of Gender, Law & Justice 2016;Winter. - Aitken M, VanderLaan DP, Wasserman L, et al. Self-Harm and Suicidality in Children Referred for Gender Dysphoria. Journal of the American Academy of Child & Adolescent Psychiatry 2016;55(6):513-20. - Arcelus J, Claes L, Witcomb GL, et al. Risk Factors for Non-Suicidal Self-Injury Among Trans Youth. The Journal of Sexual Medicine 2016;13(3):402-12. - Armand H MA. Pubertal Suppression and Professional Obligations: May a Pediatric Endocrinologist Refuse to Treat an Adolescent With Gender Dysphoria? The American Journal of Bioethics 2014;14(1):43-6. - Atienza-Macías E. Some Legal Thoughts on Transsexuality in the Healthcare System After the New Edition of the Diagnostic and Statistical Manual of Mental Disorders (DSM). Sexuality & Culture 2015;19(3):574-6. - Atkinson SR, Russell D. Gender dysphoria. Australian Family Physician 2015;44(11):792-6. - Auer MK, Hellweg R, Briken P, et al. Serum brain-derived neurotrophic factor (BDNF) is not regulated by testosterone in transmen. Biology of Sex Differences 2016;7(1):1-6. - Bachmann GA, Mussman B. The aging population: Imperative to uncouple sex and gender to establish “gender equal” health care. Maturitas 2015;80(4):421-5. - Bailey M. Transgender Workplace Discrimination in the Age of Gender Dysphoria and EDNA. Law & Psychology Review 2014;38:193-210. - *Barry KM, Farrell B, Levi JL, Vanguri N. A Bare Desire to Harm: Transgender People and the Equal Protection Clause. Boston College Law Review 2016;57(507):507-82. - Bartolucci C, Gómez-Gil E, Salamero M, et al. Sexual Quality of Life in Gender-Dysphoric Adults before Genital Sex Reassignment Surgery. The Journal of Sexual Medicine 2015;12(1):180-8. - Becker I, Nieder TO, Cerwenka S, et al. Body Image in Young Gender Dysphoric Adults: A European Multi-Center Study. Archives of Sexual Behavior 2016;45(3):559-74. - Beek TF, Cohen-Kettenis PT, Kreukels BPC. Gender incongruence/gender dysphoria and its classification history. International Review of Psychiatry 2016;28(1):5-12. - Bell F. Children with gender dysphoria and the jurisdiction of the Family Court. University of New South Wales Law Journal 2015;38(2):426-54. - Bendlin S. Gender Dysphoria in the Jailhouse: A Constitutional Right to Hormone Therapy? Cleveland State Law Review 2013;61(4):957-82. - Bockting WO. Vulnerability and Resilience Among Gender-Nonconforming Children and Adolescents: Mental Health Professionals Have a Key Role to Play. Journal of the American Academy of Child & Adolescent Psychiatry 2016;55(6):441-3. - Bockting W, Coleman E, Deutsch MB, et al. Adult development and quality of life of transgender and gender nonconforming people. Current Opinion in Endocrinology, Diabetes and Obesity 2016;23(2):188-97. - Boroughs MS, Bedoya CA, O'Cleirigh C, Safren SA. Toward Defining, Measuring, and Evaluating LGBT Cultural Competence for Psychologists. Clinical Psychology: Science and Practice 2015;22(2):151-71. - Bouman WP, de Vries ALC, T’Sjoen G. Gender Dysphoria and Gender Incongruence: An evolving inter-disciplinary field. International Review of Psychiatry 2016;28(1):1-4. - *Bouman WP, Richards C. Diagnostic and Treatment Issues for People with Gender Dysphoria in the United Kingdom. Sexual and Relationship Therapy 2013;28(3):165-71. - Brown T. Dangers of Overboard Transgender Legislation, Case Law, and Policy in Education: California's AB 1266 Dismisses Concerns about Student Safety and Privacy, The. Brigham Young University Education & Law Journal 2014;2014(2):287-319. - Burke SM, Kreukels BPC, Cohen-Kettenis PT, et al. Male-typical visuospatial functioning in gynephilic girls with gender dysphoria - organizational and activational effects of testosterone. Journal of psychiatry & neuroscience : JPN 2016;41(6):395-404. - Busari AO. Bolstering Self-Esteem as Intervention Technique in the Management of Symptoms of Gender Identity Disorder among Adolescents. Gender & Behaviour 2013;11(2):5535-45. - *Campbell MM, Artz L, Stein DJ. Sexual disorders in DSM-5 and ICD-11: a conceptual framework. Current Opinion in Psychiatry 2015;28(6):435-9. - Capetillo-Ventura NC, Jalil-Pérez SI, Motilla-Negrete K. Gender dysphoria: An overview. Medicina Universitaria 2015;17(66):53-8. - Castañeda C. Developing gender: The medical treatment of transgender young people. Social Science & Medicine 2015;143:262-70. - Castellini G. Language of self-definition in the disorders of identity. Official Journal of the Italian Society of Psychopathology Organo Ufficiale della Società Italiana di Psicopatologia 2016;22(1):39-47. - Chen M, Fuqua J, Eugster EA. Characteristics of Referrals for Gender Dysphoria Over a 13-Year Period. Journal of Adolescent Health 2016;58(3):369-71. - Ciocca G, Limoncin E, Cellerino A, et al. Gender Identity Rather Than Sexual Orientation Impacts on Facial Preferences. The Journal of Sexual Medicine 2014;11(10):2500-7. - Claes L, Bouman WP, Witcomb G, et al. Non-Suicidal Self-Injury in Trans People: Associations with Psychological Symptoms, Victimization, Interpersonal Functioning, and Perceived Social Support. The Journal of Sexual Medicine 2015;12(1):168-79. - Cohen-Kettenis PT, Klink D. Adolescents with gender dysphoria. Best Practice & Research Clinical Endocrinology & Metabolism 2015;29(3):485-95. - Colizzi M, Costa R, Todarello O. Dissociative symptoms in individuals with gender dysphoria: Is the elevated prevalence real? Psychiatry Research 2015;226(1):173-80. - Collazo A, Austin A, Craig SL. Facilitating Transition Among Transgender Clients: Components of Effective Clinical Practice. Clinical Social Work Journal 2013;41(3):228-37. - Collin L, Reisner SL, Tangpricha V, Goodman M. Prevalence of Transgender Depends on the “Case” Definition: A Systematic Review. The Journal of Sexual Medicine 2016;13(4):613-26. - Corbett K, Dimen M, Goldner V, Harris A. Talking Sex, Talking Gender—A Roundtable. Studies in Gender and Sexuality 2014;15(4):295-317. - Costa R, Dunsford M, Skagerberg E, et al. Psychological Support, Puberty Suppression, and Psychosocial Functioning in Adolescents with Gender Dysphoria. The Journal of Sexual Medicine 2015;12(11):2206-14. - Couturier J, Pindiprolu B, Findlay S, Johnson N. Anorexia nervosa and gender dysphoria in two adolescents. International Journal of Eating Disorders 2015;48(1):151-5. - Daley A, Mulé NJ. LGBTQs and the DSM-5: A Critical Queer Response. Journal of Homosexuality 2014;61(9):1288-312. - Daly TTW. Gender Dysphoria and the Ethics of Transsexual(i.e. Gender Reassignment) Surgery. Ethics & Medicine: An International Journal of Bioethics 2016;32(1):39-53. - Daniolos PT, Telingator CJ. Engendering Identity. Journal of the American Academy of Child & Adolescent Psychiatry 2013;52(12):1245-7. - Davey A, Bouman WP, Meyer C, Arcelus J. Interpersonal Functioning Among Treatment-Seeking Trans Individuals. Journal of Clinical Psychology 2015;71(12):1173-85. - Davey A, Meyer C, Arcelus J, Bouman WP. Social Support and Psychological Well-Being in Gender Dysphoria: A Comparison of Patients With Matched Controls. Journal of Sexual Medicine 2014;11(12):2976-85. - Davy Z. The DSM-5 and the Politics of Diagnosing Transpeople. Archives of Sexual Behavior 2015;44(5):1165-76. - de Vries ALC, McGuire JK, Steensma TD, et al. Young Adult Psychological Outcome After Puberty Suppression and Gender Reassignment. Pediatrics 2014;134(4):696-704. - de Vries ALC, Steensma TD, Cohen-Kettenis PT, et al. Poor peer relations predict parent- and self-reported behavioral and emotional problems of adolescents with gender dysphoria: a cross-national, cross-clinic comparative analysis. European Child & Adolescent Psychiatry 2016;25(6):579-88. - DeFeo J. Understanding Sexual, Paraphilic, and Gender Dysphoria Disorders in DSM-5. Journal of Child Sexual Abuse 2015;24(2):210-5. - Dèttore D, Ristori J, Antonelli P, et al. Gender dysphoria in adolescents: the need for a shared assessment protocol and proposal of the AGIR protocol. Journal of Psychopathology 2015;21(2):152-8. - Deuster D, Matulat P, Knief A, et al. Voice deepening under testosterone treatment in female-to-male gender dysphoric individuals. European Archives of Oto-Rhino-Laryngology 2016;273(4):959-65. - Dhejne C, Van Vlerken R, Heylens G, Arcelus J. Mental health and gender dysphoria: A review of the literature. International Review of Psychiatry 2016;28(1):44-57. - Di Ceglie D, Skagerberg E, Baron-Cohen S, Auyeung B, 16 (6). Empathising and systemising in adolescents with gender dysphoria. Opticon 1826 2014;16(6):1-8. - Drescher J. Controversies in Gender Diagnoses. LGBT Health 2013;1(1):10-4. - Drescher J, Pula J. Ethical Issues Raised by the Treatment of Gender-Variant Prepubescent Children. Hastings Center Report 2014;44(s4):S17-S22. - Drescher J. Queer diagnoses revisited: The past and future of homosexuality and gender diagnoses in DSM and ICD. International Review of Psychiatry 2015:1-10. - Drescher J. Gender Policing in the Clinical Setting: Discussion of Sandra Silverman’s “The Colonized Mind: Gender, Trauma, and Mentalization”. Psychoanalytic Dialogues 2015;25(1):67-76. - Eapen V, Črnčec R. DSM 5 and child psychiatric disorders: What is new? What has changed? Asian Journal of Psychiatry 2014;11:114-8. - Ellis SJ, Bailey L, McNeil J. Trans People's Experiences of Mental Health and Gender Identity Services: A UK Study. Journal of Gay & Lesbian Mental Health 2015;19(1):4-20. - Esteva de Antonio I, Gómez-Gil E, Group aG. Coordination of healthcare for transsexual persons: a multidisciplinary approach. Current Opinion in Endocrinology, Diabetes and Obesity 2013;20(6):585-91. - Fabris B, Bernardi S, Trombetta C. Cross-sex hormone therapy for gender dysphoria. Journal Of Endocrinological Investigation 2015;38(3):269-82. - Feusner JD, Dervisic J, Kosidou K, et al. Female-to-Male Transsexual Individuals Demonstrate Different Own Body Identification. Archives of Sexual Behavior 2016;45(3):525-36. - Firth MT. Childhood abuse, depressive vulnerability and gender dysphoria: Part 2. Counselling & Psychotherapy Research 2015;15(2):98-108 11p. - Fisher AD, Castellini G, Casale H, et al. Hypersexuality, Paraphilic Behaviors, and Gender Dysphoria in Individuals with Klinefelter's Syndrome. The Journal of Sexual Medicine 2015;12(12):2413-24. - Fitzgibbons RP. Transsexual attractions and sexual reassignment surgery: Risks and potential risks. The Linacre Quarterly 2015;82(4):337-50. - Fuss J, Auer MK, Briken P. Gender dysphoria in children and adolescents: a review of recent research. Current Opinion in Psychiatry 2015;28(6):430-4. - Gava G, Cerpolini S, Martelli V, et al. Cyproterone acetate vs leuprolide acetate in combination with transdermal oestradiol in transwomen: a comparison of safety and effectiveness. Clinical Endocrinology 2016;85(2):239-46. - Giami A, Beaubatie E. Gender Identification and Sex Reassignment Surgery in the Trans Population: A Survey Study in France. Archives of Sexual Behavior 2014;43(8):1491-501. - Görtz DP, Commons ML. The stage-value model: Implications for the changing standards of care. International Journal of Law and Psychiatry 2015;42–43:135-43. - Gray SAO, Sweeney KK, Randazzo R, Levitt HM. “Am I Doing the Right Thing?”: Pathways to Parenting a Gender Variant Child. Family Process 2016;55(1):123-38. - Gregor C, Davidson S, Hingley-Jones H. The experience of gender dysphoria for pre-pubescent children and their families: a review of the literature. Child & Family Social Work 2014;21(3):339-46. - Gregor C, Hingley-Jones H, Davidson S. Understanding the Experience of Parents of Pre-pubescent Children with Gender Identity Issues. Child and Adolescent Social Work Journal 2015;32(3):237-46. - Güldenring A. A critical view of transgender health care in Germany: Psychopathologizing gender identity – Symptom of ‘disordered’ psychiatric/psychological diagnostics? International Review of Psychiatry 2015;27(5):427-34. - Guzman-Parra J, Paulino-Matos P, de Diego-Otero Y, et al. Substance Use and Social Anxiety in Transsexual Individuals. Journal of Dual Diagnosis 2014;10(3):162-7. - Hardy TLD, Boliek CA, Wells K, Rieger JM. The ICF and Male-to-Female Transsexual Communication. International Journal of Transgenderism 2013;14(4):196-208. - Heylens G, Elaut E, Verschelden G, Cuypere GD. Transgender Persons Applying for Euthanasia in Belgium: A Case Report and Implications for Assessment and Treatment. Journal of Psychiatry 2016;19(1):347. - Hill T. Transgender Military Inmates' Legal and Constitutional Rights to Medical Care in Prisons: Serious Medical Need versus Military Necessity. Vermont Law Review 2014;39(2):411-59. - Holt V, Skagerberg E, Dunsford M. Young people with features of gender dysphoria: Demographics and associated difficulties. Clinical Child Psychology and Psychiatry 2016;21(1):108-18. - *Hoffman B. An Overview of Depression among Transgender Women. Depression Research and Treatment 2014;2014:1-9. - Jacobs LA, Rachlin K, Erickson-Schroth L, Janssen A. Gender Dysphoria and Co-Occurring Autism Spectrum Disorders: Review, Case Examples, and Treatment Considerations. LGBT Health 2014;1(4):277-82. - Joel D, Tarrasch R, Berman Z, et al. Queering gender: studying gender identity in ‘normative’ individuals. Psychology & Sexuality 2014;5(4):291-321. - *Johnson L, Shipherd J, Walton HM. The psychologist’s role in transgender-specific care with U.S. veterans. Psychological Services 2016;13(1):69-77. - Jones BA, Haycraft E, Murjan S, Arcelus J. Body dissatisfaction and disordered eating in trans people: A systematic review of the literature. International Review of Psychiatry 2016;28(1):81-94. - Judge C, O’Donovan C, Callaghan G, et al. Gender Dysphoria – Prevalence and Co-Morbidities in an Irish Adult Population. Frontiers in Endocrinology 2014;5(87). - Junger J, Habel U, Bröhr S, et al. More than Just Two Sexes: The Neural Correlates of Voice Gender Perception in Gender Dysphoria. PLoS ONE 2014;9(11):1-12. - Kalra G, Tandon A, Sathyanarayana Rao TS. Sexual disorders in Asians: A review. Asian Journal of Psychiatry 2014;7:80-2. - Kelly F. Australian children living with gender dysphoria: does the Family Court have a role to play? Journal of law and medicine 2014;22(1):105-20. - Kern L, Edmonds P, Perrin EC, Stein MT. An 8-year-old Biological Female Who Identifies Herself as a Boy: Perspectives in Primary Care and from a Parent. Journal of Developmental & Behavioral Pediatrics 2014;35(4):301-3. - Kon AA. Transgender Children and Adolescents. The American Journal of Bioethics 2014;14(1):48-50. - Kraus C. Classifying Intersex in DSM-5: Critical Reflections on Gender Dysphoria. Archives of Sexual Behavior 2015;44(5):1147-63. - *Kreukels BPC, Guillamon A. Neuroimaging studies in people with gender incongruence. International Review of Psychiatry 2016;28(1):120-8.Kristensen ZE, Broome MR. Autistic Traits in an Internet Sample of Gender Variant UK Adults. International Journal of Transgenderism 2015;16(4):234-45. - *Leibowitz S, de Vries ALC. Gender dysphoria in adolescence. International Review of Psychiatry 2016;28(1):21-35. - Lev AI. Gender Dysphoria: Two Steps Forward, One Step Back. Clinical Social Work Journal 2013;41(3):288-96. - Levine DA, Braverman PK, Adelman WP, et al. Office-Based Care for Lesbian, Gay, Bisexual, Transgender, and Questioning Youth. Pediatrics 2013;132(1):198-203. - Levman J, Takahashi E. Multivariate analyses applied to fetal, neonatal and pediatric MRI of neurodevelopmental disorders. NeuroImage: Clinical 2015;9:532-44. - Li F, Rendall D, Vasey PL, et al. The development of sex/gender-specific /s/ and its relationship to gender identity in children and adolescents. Journal of Phonetics 2016;57:59-70. - Majumder A, Sanyal D. Outcome and preferences in female-to-male subjects with gender dysphoria: Experience from Eastern India. Indian Journal of Endocrinology & Metabolism 2016;20(3):308-11. - Marshall J, Cooper M, Rudnick A. Gender Dysphoria and Dementia: A Case Report. Journal of Gay & Lesbian Mental Health 2015;19(1):112-7. - Marshall E, Claes L, Bouman WP, et al. Non-suicidal self-injury and suicidality in trans people: A systematic review of the literature. International Review of Psychiatry 2016;28(1):58-69. - Matza AR, Sloan CA, Kauth MR. Quality LGBT Health Education: A Review of Key Reports and Webinars. Clinical Psychology: Science & Practice 2015;22(2):127-44. - Meriggiola MC, Gava G. Endocrine care of transpeople part I. A review of cross-sex hormonal treatments, outcomes and adverse effects in transmen. Clinical Endocrinology 2015;83(5):597-606. - Moleiro C, Pinto N. Sexual Orientation and Gender Identity: Review of concepts, controversies and their relation to psychopathology classification systems. Frontiers in Psychology, 2015; v. 6. - Nelson JL. Medicine and Making Sense of Queer Lives. Hastings Center Report 2014;44(s4):S12-S6. - Nieder TO, Elaut E, Richards C, Dekker A. Sexual orientation of trans adults is not linked to outcome of transition-related health care, but worth asking. International Review of Psychiatry 2016;28(1):103-11. - Nygren U, Nordenskjöld A, Arver S, Södersten M. Effects on Voice Fundamental Frequency and Satisfaction with Voice in Trans Men during Testosterone Treatment—A Longitudinal Study. Journal of Voice 2016;30(6):766 e23- e34. - Olson KR, Durwood L, DeMeules M, McLaughlin KA. Mental Health of Transgender Children Who Are Supported in Their Identities. Pediatrics 2016;137(3):e 20153223. - Olson J, Schrager SM, Belzer M, et al. Baseline Physiologic and Psychosocial Characteristics of Transgender Youth Seeking Care for Gender Dysphoria. Journal of Adolescent Health 2015;57(4):374-80. - Osborne CS, Lawrence AA. Male Prison Inmates With Gender Dysphoria: When Is Sex Reassignment Surgery Appropriate? Archives of Sexual Behavior 2016:1-15. - Parco JE, Levy DA, Spears SR. Transgender Military Personnel in the Post-DADT Repeal Era: A Phenomenological Study. Armed Forces & Society 2014. - Pasterski V, Zucker KJ, Hindmarsh PC, et al. Increased Cross-Gender Identification Independent of Gender Role Behavior in Girls with Congenital Adrenal Hyperplasia: Results from a Standardized Assessment of 4- to 11-Year-Old Children. Archives of Sexual Behavior 2015;44(5):1363-75. - Prunas A, Vitelli R, Agnello F, et al. Defensive functioning in MtF and FtM transsexuals. Comprehensive Psychiatry 2014;55(4):966-71. - Quam K. Unfinished Business of Repealing Don't Ask, Don't Tell: The Military's Unconstitutional Ban on Transgender Individuals. Utah Law Review 2015(3):721-41. - Rabito-Alcón MF, Rodríguez-Molina JM. Satisfaction with life and psychological well-being in people with gender dysphoria. Actas espanolas de psiquiatria 2016;44(2):47-54. - Rajkumar RP. Gender Identity Disorder and Schizophrenia: Neurodevelopmental Disorders with Common Causal Mechanisms? Schizophrenia Research and Treatment 2014;2014:8. - Rathi A, Bhatia MS. Management challenges in a case of gender identity disorder. Industrial Psychiatry Journal 2014;23(2):157-9. - Reisner SL, Vetters R, Leclerc M, et al. Mental Health of Transgender Youth in Care at an Adolescent Urban Community Health Center: A Matched Retrospective Cohort Study. Journal of Adolescent Health 2015;56(3):274-9. - Richards C, Bouman WP, Seal L, et al. Non-binary or genderqueer genders. International Review of Psychiatry 2016;28(1):95-102. - Riggs DW, Coleman K, Due C. Healthcare experiences of gender diverse Australians: a mixed-methods, self-report survey. BMC Public Health 2014;14(1):1-5. - Riggs DW, Due C. Support Experiences and Attitudes of Australian Parents of Gender Variant Children. Journal of Child and Family Studies 2015;24(7):1999-2007. - Ristori J, Steensma TD. Gender dysphoria in childhood. International Review of Psychiatry 2016;28(1):13-20. - Roberts TK, Fantz CR. Barriers to quality health care for the transgender population. Clinical Biochemistry 2014;47(10–11):983-7. - Rosenthal SM. Approach to the Patient: Transgender Youth: Endocrine Considerations. The Journal of Clinical Endocrinology & Metabolism 2014;99(12):4379-89. - Rosky CJ. No Promo Hetero: Children's Right to be Queer. Cardozo Law Review 2013;35(2):425-510. - Ross A. The Invisible Army: Why the Military Needs to Rescind Its Ban on Transgender Service Members. Southern California Interdisciplinary Law Journal 2014;23:185-216. - Sanyal D, Majumder A. Presentation of gender dysphoria: A perspective from Eastern India. Indian Journal of Endocrinology & Metabolism 2016;20(1):129-33. - Schmidt L, Levine R. Psychological Outcomes and Reproductive Issues Among Gender Dysphoric Individuals. Endocrinology and Metabolism Clinics of North America 2015;44(4):773-85. - Schneider C, Cerwenka S, Nieder TO, et al. Measuring Gender Dysphoria: A Multicenter Examination and Comparison of the Utrecht Gender Dysphoria Scale and the Gender Identity/Gender Dysphoria Questionnaire for Adolescents and Adults. Archives of Sexual Behavior 2016;45(3):551-8. - Schwarz K, Fontanari AMV, Mueller A, et al. Transsexual Voice Questionnaire for Male-to-female Brazilian Transsexual People. Journal of Voice 2017;31(1):e15-e20. - Schwarz K, Fontanari AMV, Mueller A, et al. Neural Correlates of Psychosis and Gender Dysphoria in an Adult Male. Archives of Sexual Behavior 2016;45(3):761-5. - Seal LJ. A review of the physical and metabolic effects of cross-sex hormonal therapy in the treatment of gender dysphoria. Annals of Clinical Biochemistry: An international journal of biochemistry and laboratory medicine 2016;53(1):10-20. - Selekman J, Diefenbeck C. The New DSM-5 and Its Impact on the Mental Health Care of Children. Journal of Pediatric Nursing 2014;29(5):442-50. - Shumer DE, Nokoff NJ, Spack NP. Advances in the Care of Transgender Children and Adolescents. Advances in Pediatrics 2016;63(1):79-102. - Shumer DE, Reisner SL, Edwards-Leeper L, Tishelman A. Evaluation of Asperger Syndrome in Youth Presenting to a Gender Dysphoria Clinic. LGBT Health 2016;3(5):387-90. - Shumer DE, Tishelman AC. The Role of Assent in the Treatment of Transgender Adolescents. International Journal of Transgenderism 2015;16(2):97-102. - Simons L, Leibowitz S, Hidalgo M. Understanding Gender Variance in Children and Adolescents. Pediatric Annals 2014;43(6):e126-e31. - Skagerberg E, Davidson S, Carmichael P. Internalizing and Externalizing Behaviors in a Group of Young People with Gender Dysphoria. International Journal of Transgenderism 2013;14(3):105-12. - Skagerberg E, Di Ceglie D, Carmichael P. Brief Report: Autistic Features in Children and Adolescents with Gender Dysphoria. Journal Of Autism And Developmental Disorders 2015;45(8):2628-32. - Skagerberg E, Parkinson R, Carmichael P. Self-Harming Thoughts and Behaviors in a Group of Children and Adolescents with Gender Dysphoria. International Journal of Transgenderism 2013;14(2):86-92. - Smith ES, Junger J, Derntl B, Habel U. The transsexual brain – A review of findings on the neural basis of transsexualism. Neuroscience & Biobehavioral Reviews 2015;59:251-66. - Smith MK, Mathews B. Treatment for gender dysphoria in children: the new legal, ethical and clinical landscape. The Medical Journal of Australia 2015;202(2):102-4. - Smith FD. Perioperative Care of the Transgender Patient. AORN Journal 2016;103(2):151-63. - Södersten M, Nygren U, Hertegård S, Dhejne C. Interdisciplinary Program in Sweden Related to Transgender Voice. SIG 3 Perspectives on Voice and Voice Disorders 2015;25(2):87-97. - Staphorsius AS, Kreukels BPC, Cohen-Kettenis PT, et al. Puberty suppression and executive functioning: An fMRI-study in adolescents with gender dysphoria. Psychoneuroendocrinology 2015;56:190-9. - Strandjord SE, Ng H, Rome ES. Effects of treating gender dysphoria and anorexia nervosa in a transgender adolescent: Lessons learned. International Journal of Eating Disorders 2015;48(7):942-5. - Stroumsa D. The State of Transgender Health Care: Policy, Law, and Medical Frameworks. American Journal of Public Health 2014;104(3):e31-e8. - Taylor LA. A Win for Transgender Employees: Chevron Deference for the EEOC'S Decision in Macy V. Holder. Journal of Law & Family Studies 2013;15(1):181-207. - Thompson D. Commentary on “Gender disorders in learning disabilities – a systematic review”. Tizard Learning Disability Review 2014;19(4):166-9. - Toscano ME, Maynard E. Understanding the Link: “Homosexuality,” Gender Identity, and the DSM. Journal of LGBT Issues in Counseling 2014;8(3):248-63. - Travis M. Accommodating Intersexuality in European Union Anti-Discrimination Law. European Law Journal 2015;21(2):180-99. - Trevor M, Boddy J. Transgenderism and Australian Social Work: A Literature Review. Australian Social Work 2013;66(4):555-70. - Turan Ş, Poyraz CA, Öcek Baş T, et al. Affective temperaments in subjects with female-to-male gender dysphoria. Journal of Affective Disorders 2015;176:61-4. - van de Grift TC, Cohen-Kettenis PT, Elaut E, et al. A network analysis of body satisfaction of people with gender dysphoria. Body Image 2016;17:184-90. - Van Der Miesen AIR, Hurley H, De Vries ALC. Gender dysphoria and autism spectrum disorder: A narrative review. International Review of Psychiatry 2016;28(1):70-80. - van Schalkwyk GI, Klingensmith K, Volkmar FR. Gender identity and autism spectrum disorders. The Yale journal of biology and medicine 2015;88(1):81-3. - Vance SR, Ehrensaft D, Rosenthal SM. Psychological and Medical Care of Gender Nonconforming Youth. Pediatrics 2014;134(6):1184-92. - VanderLaan DP, Blanchard R, Wood H, Zucker KJ. Birth Order and Sibling Sex Ratio of Children and Adolescents Referred to a Gender Identity Service. PloS One 2014;9(3):e90257. - VanderLaan DP, Leef JH, Wood H, et al. Autism Spectrum Disorder Risk Factors and Autistic Traits in Gender Dysphoric Children. Journal of Autism and Developmental Disorders 2015;45(6):1742-50. - VanderLaan DP, Postema L, Wood H, et al. Do Children With Gender Dysphoria Have Intense/Obsessional Interests? The Journal of Sex Research 2015;52(2):213-9. - Veltman A, Chaimowitz G. Mental Health Care for People Who Identify as Lesbian, Gay, Bisexual, Transgender, and (or) Queer. Canadian Journal of Psychiatry. Revue Canadienne de Psychiatrie 2014;59(11):1-7. - Vitelli R. Adult Male-to-Female Transsexualism A Clinical Existential-Phenomenological Inquiry. Journal of Phenomenological Psychology 2015;46(1):33-68. - Vrouenraets LJJJ, Fredriks AM, Hannema SE, et al. Early Medical Treatment of Children and Adolescents With Gender Dysphoria: An Empirical Ethical Study. Journal of Adolescent Health 2015;57(4):367-73. - Vrouenraets LJJJ, Fredriks AM, Hannema SE, et al. Perceptions of Sex, Gender, and Puberty Suppression: A Qualitative Analysis of Transgender Youth. Archives of Sexual Behavior 2016:1-7. - Wallace SA, Blough KL, Kondapalli LA. Fertility preservation in the transgender patient: expanding oncofertility care beyond cancer. Gynecological Endocrinology 2014;30(12):868-71. - Wangjiraniran B, Selvaggi G, Chokrungvaranont P, et al. Male-to-female vaginoplasty: Preecha’s surgical technique. Journal of Plastic Surgery and Hand Surgery 2015;49(3):153-9. - Washburn M. Five Things Social Workers Should Know about the DSM-5. Social Work 2013;58(4):373-6. - White Hughto JM, Reisner SL, Pachankis JE. Transgender stigma and health: A critical review of stigma determinants, mechanisms, and interventions. Social Science & Medicine 2015;147:222-31. - Witcomb GL, Bouman WP, Brewin N, et al. Body Image Dissatisfaction and Eating-Related Psychopathology in Trans Individuals: A Matched Control Study. European Eating Disorders Review 2015;23(4):287-93. - Withers R. The seventh penis: towards effective psychoanalytic work with pre-surgical transsexuals. Journal of Analytical Psychology 2015;60(3):390-412. - Zucker KJ, Seto MC. Gender dysphoria and paraphilic sexual disorders. In: Thapar A, Pine DS, Leckman JF, et al., eds. Rutter's Child and Adolescent Psychiatry. Chichester: John Wiley & Sons, 2015. - Zucker KJ, Lawrence AA, Kreukels BPC. Gender Dysphoria in Adults. Annual Review of Clinical Psychology 2016;12(1):217-47. |
| --- |
