# Supplementary material for: What Is Gender Dysphoria? A Critical Systematic Narrative Review
Source: Transgend Health. 2018 Nov 1;3(1):159–69. doi: 10.1089/trgh.2018.0014 (PMC6225591; doi:10.1089/trgh.2018.0014)
Supplement: Supplemental data [file Supp_Table3.docx]

Supplementary Table S3. References: Depathologization of gender non-conformity

| - Antoni C. Service Delivery and the Challenges of Providing Service to People Who Are Transgender. SIG 3 Perspectives on Voice and Voice Disorders 2015;25(2):59-65. - Atienza-Macías E. Some Legal Thoughts on Transsexuality in the Healthcare System After the New Edition of the Diagnostic and Statistical Manual of Mental Disorders (DSM). Sexuality & Culture 2015;19(3):574-6. - Atkinson SR, Russell D. Gender dysphoria. Australian Family Physician 2015;44(11):792-6. - Azul D. On the Varied and Complex Factors Affecting Gender Diverse People's Vocal Situations: Implications for Clinical Practice. SIG 3 Perspectives on Voice and Voice Disorders 2015;25(2):75-86. - Azul D. Transmasculine people's vocal situations: a critical review of gender-related discourses and empirical data. International Journal of Language & Communication Disorders 2015;50(1):31-47. - Bachmann GA, Mussman B. The aging population: Imperative to uncouple sex and gender to establish “gender equal” health care. Maturitas 2015;80(4):421-5. - Bailey M. Transgender Workplace Discrimination in the Age of Gender Dysphoria and EDNA. Law & Psychology Review 2014;38:193-210. - Baral SD, Poteat T, Strömdahl S, et al. Worldwide burden of HIV in transgender women: a systematic review and meta-analysis. The Lancet Infectious Diseases 2013;13(3):214-22. - Barry KM, Farrell B, Levi JL, Vanguri N. A Bare Desire to Harm: Transgender People and the Equal Protection Clause. Boston College Law Review 2016;57(507):507-82. - Beek TF, Cohen-Kettenis PT, Kreukels BPC. Gender incongruence/gender dysphoria and its classification history. International Review of Psychiatry 2016;28(1):5-12. - Boroughs MS, Bedoya CA, O'Cleirigh C, Safren SA. Toward Defining, Measuring, and Evaluating LGBT Cultural Competence for Psychologists. Clinical Psychology: Science and Practice 2015;22(2):151-71. - Bouman WP, Richards C. Diagnostic and Treatment Issues for People with Gender Dysphoria in the United Kingdom. Sexual and Relationship Therapy 2013;28(3):165-71. - Campbell MM, Artz L, Stein DJ. Sexual disorders in DSM-5 and ICD-11: a conceptual framework. Current Opinion in Psychiatry 2015;28(6):435-9. - Collazo A, Austin A, Craig SL. Facilitating Transition Among Transgender Clients: Components of Effective Clinical Practice. Clinical Social Work Journal 2013;41(3):228-37. - Drescher J. Queer diagnoses revisited: The past and future of homosexuality and gender diagnoses in DSM and ICD. International Review of Psychiatry 2015:1-10. - Eapen V, Črnčec R. DSM 5 and child psychiatric disorders: What is new? What has changed? Asian Journal of Psychiatry 2014;11:114-8. - Fabris B, Bernardi S, Trombetta C. Cross-sex hormone therapy for gender dysphoria. Journal Of Endocrinological Investigation 2015;38(3):269-82. - Gray SAO, Sweeney KK, Randazzo R, Levitt HM. “Am I Doing the Right Thing?”: Pathways to Parenting a Gender Variant Child. Family Process 2016;55(1):123-38. - Güldenring A. A critical view of transgender health care in Germany: Psychopathologizing gender identity – Symptom of ‘disordered’ psychiatric/psychological diagnostics? International Review of Psychiatry 2015;27(5):427-34. - Johnson L, Shipherd J, Walton HM. The psychologist’s role in transgender-specific care with U.S. veterans. Psychological Services 2016;13(1):69-77. - Kelso T. Still Trapped in the U.S. Media’s Closet: Representations of Gender-Variant, Pre-Adolescent Children. Journal of Homosexuality 2015;62(8):1058-97. - Kon AA. Transgender Children and Adolescents. The American Journal of Bioethics 2014;14(1):48-50. - Kraus C. Classifying Intersex in DSM-5: Critical Reflections on Gender Dysphoria. Archives of Sexual Behavior 2015;44(5):1147-63. - Leibowitz S, de Vries ALC. Gender dysphoria in adolescence. International Review of Psychiatry 2016;28(1):21-35. - Lev AI. Gender Dysphoria: Two Steps Forward, One Step Back. Clinical Social Work Journal 2013;41(3):288-96. - Levine DA, Braverman PK, Adelman WP, et al. Office-Based Care for Lesbian, Gay, Bisexual, Transgender, and Questioning Youth. Pediatrics 2013;132(1):198-203. - Mepham N, Bouman WP, Arcelus J, et al. People with Gender Dysphoria Who Self-Prescribe Cross-Sex Hormones: Prevalence, Sources, and Side Effects Knowledge. The Journal of Sexual Medicine 2014;11(12):2995-3001. - Moleiro C, Pinto N. Sexual Orientation and Gender Identity: Review of concepts, controversies and their relation to psychopathology classification systems. Frontiers in Psychology, 2015; v. 6. - Nelson JL. Medicine and Making Sense of Queer Lives. Hastings Center Report 2014;44(s4):S12-S6. - Olson KR, Durwood L, DeMeules M, McLaughlin KA. Mental Health of Transgender Children Who Are Supported in Their Identities. Pediatrics 2016;137(3):e 20153223. - Olson J, Schrager SM, Belzer M, et al. Baseline Physiologic and Psychosocial Characteristics of Transgender Youth Seeking Care for Gender Dysphoria. Journal of Adolescent Health 2015;57(4):374-80. - Parco JE, Levy DA, Spears SR. Transgender Military Personnel in the Post-DADT Repeal Era: A Phenomenological Study. Armed Forces & Society 2014. - Quam K. Unfinished Business of Repealing Don't Ask, Don't Tell: The Military's Unconstitutional Ban on Transgender Individuals. Utah Law Review 2015(3):721-41. - Reisner SL, Vetters R, Leclerc M, et al. Mental Health of Transgender Youth in Care at an Adolescent Urban Community Health Center: A Matched Retrospective Cohort Study. Journal of Adolescent Health 2015;56(3):274-9. - Roberts TK, Fantz CR. Barriers to quality health care for the transgender population. Clinical Biochemistry 2014;47(10–11):983-7. - Rosenthal SM. Approach to the Patient: Transgender Youth: Endocrine Considerations. The Journal of Clinical Endocrinology & Metabolism 2014;99(12):4379-89. - Ross A. The Invisible Army: Why the Military Needs to Rescind Its Ban on Transgender Service Members. Southern California Interdisciplinary Law Journal 2014;23:185-216. - Sanyal D, Majumder A. Presentation of gender dysphoria: A perspective from Eastern India. Indian Journal of Endocrinology & Metabolism 2016;20(1):129-33. - Schneider C, Cerwenka S, Nieder TO, et al. Measuring Gender Dysphoria: A Multicenter Examination and Comparison of the Utrecht Gender Dysphoria Scale and the Gender Identity/Gender Dysphoria Questionnaire for Adolescents and Adults. Archives of Sexual Behavior 2016;45(3):551-8. - Shumer DE, Nokoff NJ, Spack NP. Advances in the Care of Transgender Children and Adolescents. Advances in Pediatrics 2016;63(1):79-102. - Simons L, Leibowitz S, Hidalgo M. Understanding Gender Variance in Children and Adolescents. Pediatric Annals 2014;43(6):e126-e31. - Smith ES, Junger J, Derntl B, Habel U. The transsexual brain – A review of findings on the neural basis of transsexualism. Neuroscience & Biobehavioral Reviews 2015;59:251-66. - Smith FD. Perioperative Care of the Transgender Patient. AORN Journal 2016;103(2):151-63. - Stroumsa D. The State of Transgender Health Care: Policy, Law, and Medical Frameworks. American Journal of Public Health 2014;104(3):e31-e8. - Thompson D. Commentary on “Gender disorders in learning disabilities – a systematic review”. Tizard Learning Disability Review 2014;19(4):166-9. - Trevor M, Boddy J. Transgenderism and Australian Social Work: A Literature Review. Australian Social Work 2013;66(4):555-70. - van de Grift TC, Cohen-Kettenis PT, Steensma TD, et al. Body Satisfaction and Physical Appearance in Gender Dysphoria. Archives of Sexual Behavior 2016;45(3):575-85. - Vaughn M, Silver K, Murphy S, et al. Women with Disabilities Discuss Sexuality in San Francisco Focus Groups. Sexuality and Disability 2015;33(1):19-46. - Wallace SA, Blough KL, Kondapalli LA. Fertility preservation in the transgender patient: expanding oncofertility care beyond cancer. Gynecological Endocrinology 2014;30(12):868-71. - Washburn M. Five Things Social Workers Should Know about the DSM-5. Social Work 2013;58(4):373-6. - White Hughto JM, Reisner SL, Pachankis JE. Transgender stigma and health: A critical review of stigma determinants, mechanisms, and interventions. Social Science & Medicine 2015;147:222-31. - Withers R. The seventh penis: towards effective psychoanalytic work with pre-surgical transsexuals. Journal of Analytical Psychology 2015;60(3):390-412. - Zucker KJ, Seto MC. Gender dysphoria and paraphilic sexual disorders. In: Thapar A, Pine DS, Leckman JF, et al., eds. Rutter's Child and Adolescent Psychiatry. Chichester: John Wiley & Sons, 2015. - Zucker KJ, Lawrence AA, Kreukels BPC. Gender Dysphoria in Adults. Annual Review of Clinical Psychology 2016;12(1):217-47. |
| --- |
