# Supplementary material for: What Is Gender Dysphoria? A Critical Systematic Narrative Review
Source: Transgend Health. 2018 Nov 1;3(1):159–69. doi: 10.1089/trgh.2018.0014 (PMC6225591; doi:10.1089/trgh.2018.0014)
Supplement: Supplemental data [file Supp_Table4.docx]

Supplementary Table S4. References Human Diversity

| - Ahmad S, Barrett J, Beaini AY, et al. Gender dysphoria services: a guide for general practitioners and other healthcare staff. Sexual and Relationship Therapy 2013;28(3):172-85. - Atkinson SR, Russell D. Gender dysphoria. Australian Family Physician 2015;44(11):792-6. - Bachmann GA, Mussman B. The aging population: Imperative to uncouple sex and gender to establish “gender equal” health care. Maturitas 2015;80(4):421-5. - Barry KM, Farrell B, Levi JL, Vanguri N. A Bare Desire to Harm: Transgender People and the Equal Protection Clause. Boston College Law Review 2016;57(507):507-82. - Bell F. Children with gender dysphoria and the jurisdiction of the Family Court. University of New South Wales Law Journal 2015;38(2):426-54. - Bockting WO. Vulnerability and Resilience Among Gender-Nonconforming Children and Adolescents: Mental Health Professionals Have a Key Role to Play. Journal of the American Academy of Child & Adolescent Psychiatry 2016;55(6):441-3. - Bouman WP, Richards C, Addinall RM, et al. Yes and yes again: are standards of care which require two referrals for genital reconstructive surgery ethical? Sexual and Relationship Therapy 2014;29(4):377-89. - Castellini G. Language of self-definition in the disorders of identity. Official Journal of the Italian Society of Psychopathology Organo Ufficiale della Società Italiana di Psicopatologia 2016;22(1):39-47. - Kaltiala-Heino R, Sumia M, Työläjärvi M, Lindberg N. Two years of gender identity service for minors: overrepresentation of natal girls with severe problems in adolescent development. Child and Adolescent Psychiatry and Mental Health 2015;1(9):1-9. - Lev AI. Gender Dysphoria: Two Steps Forward, One Step Back. Clinical Social Work Journal 2013;41(3):288-96. - Parco JE, Levy DA, Spears SR. Transgender Military Personnel in the Post-DADT Repeal Era: A Phenomenological Study. Armed Forces & Society 2014. - Quam K. Unfinished Business of Repealing Don't Ask, Don't Tell: The Military's Unconstitutional Ban on Transgender Individuals. Utah Law Review 2015(3):721-41. - Reisner SL, Vetters R, Leclerc M, et al. Mental Health of Transgender Youth in Care at an Adolescent Urban Community Health Center: A Matched Retrospective Cohort Study. Journal of Adolescent Health 2015;56(3):274-9. - Richards C, Bouman WP, Seal L, et al. Non-binary or genderqueer genders. International Review of Psychiatry 2016;28(1):95-102. - Ross A. The Invisible Army: Why the Military Needs to Rescind Its Ban on Transgender Service Members. Southern California Interdisciplinary Law Journal 2014;23:185-216. - Smith FD. Perioperative Care of the Transgender Patient. AORN Journal 2016;103(2):151-63. - Stroumsa D. The State of Transgender Health Care: Policy, Law, and Medical Frameworks. American Journal of Public Health 2014;104(3):e31-e8. - van de Grift TC, Cohen-Kettenis PT, Steensma TD, et al. Body Satisfaction and Physical Appearance in Gender Dysphoria. Archives of Sexual Behavior 2016;45(3):575-85. - van Schalkwyk GI, Klingensmith K, Volkmar FR. Gender identity and autism spectrum disorders. The Yale journal of biology and medicine 2015;88(1):81-3. |
| --- |
