# Supplementary material for: What Is Gender Dysphoria? A Critical Systematic Narrative Review
Source: Transgend Health. 2018 Nov 1;3(1):159–69. doi: 10.1089/trgh.2018.0014 (PMC6225591; doi:10.1089/trgh.2018.0014)
Supplement: Supplemental data [file Supp_Table5.docx]

Supplementary Table S5. References: Change in nomenclature from GID to GD would reduce this stigma

| - Acton LA. Overturning in re gardiner: Ending transgender discrimination in kansas. Family Law Quarterly, 2014; v. 48. - Aiken J. Promoting an Integrated Approach to Ensuring Access to Gender Incongruent Health Care. Berkeley Journal of Gender, Law & Justice 2016;Winter. - Arcelus J, Claes L, Witcomb GL, et al. Risk Factors for Non-Suicidal Self-Injury Among Trans Youth. The Journal of Sexual Medicine 2016;13(3):402-12. - Armand H MA. Pubertal Suppression and Professional Obligations: May a Pediatric Endocrinologist Refuse to Treat an Adolescent With Gender Dysphoria? The American Journal of Bioethics 2014;14(1):43-6. - Atkinson SR, Russell D. Gender dysphoria. Australian Family Physician 2015;44(11):792-6. - Bailey L, Ellis SJ, McNeil J. Suicide risk in the UK trans population and the role of gender transition in decreasing suicidal ideation and suicide attempt. Mental Health Review Journal 2014;19(4):209-20. - Bailey M. Transgender Workplace Discrimination in the Age of Gender Dysphoria and EDNA. Law & Psychology Review 2014;38:193-210. - Barry KM, Farrell B, Levi JL, Vanguri N. A Bare Desire to Harm: Transgender People and the Equal Protection Clause. Boston College Law Review 2016;57(507):507-82. - Bell F. Children with gender dysphoria and the jurisdiction of the Family Court. University of New South Wales Law Journal 2015;38(2):426-54. - Bockting WO, Miner MH, Swinburne Romine RE, et al. Stigma, Mental Health, and Resilience in an Online Sample of the US Transgender Population. American Journal of Public Health 2013;103(5):943-51. - Bodoin EM, Byrd CT, Adler RK. The Clinical Profile of the Male-to-Female Transgender Person of the 21st Century. Contemporary Issues in Communication Science & Disorders 2014;41:39-54. - Boroughs MS, Bedoya CA, O'Cleirigh C, Safren SA. Toward Defining, Measuring, and Evaluating LGBT Cultural Competence for Psychologists. Clinical Psychology: Science and Practice 2015;22(2):151-71. - Bouman WP, de Vries ALC, T’Sjoen G. Gender Dysphoria and Gender Incongruence: An evolving inter-disciplinary field. International Review of Psychiatry 2016;28(1):1-4. - Brown T. Dangers of Overboard Transgender Legislation, Case Law, and Policy in Education: California's AB 1266 Dismisses Concerns about Student Safety and Privacy, The. Brigham Young University Education & Law Journal 2014;2014(2):287-319. - Castellini G. Language of self-definition in the disorders of identity. Official Journal of the Italian Society of Psychopathology Organo Ufficiale della Società Italiana di Psicopatologia 2016;22(1):39-47. - Costantino A, Cerpolini S, Alvisi S, et al. A Prospective Study on Sexual Function and Mood in Female-to-Male Transsexuals During Testosterone Administration and After Sex Reassignment Surgery. Journal of Sex & Marital Therapy 2013;39(4):321-36. - Daley A, Mulé NJ. LGBTQs and the DSM-5: A Critical Queer Response. Journal of Homosexuality 2014;61(9):1288-312. - Davey A, Meyer C, Arcelus J, Bouman WP. Social Support and Psychological Well-Being in Gender Dysphoria: A Comparison of Patients With Matched Controls. Journal of Sexual Medicine 2014;11(12):2976-85. - Davy Z. The DSM-5 and the Politics of Diagnosing Transpeople. Archives of Sexual Behavior 2015;44(5):1165-76. - Dhejne C, Van Vlerken R, Heylens G, Arcelus J. Mental health and gender dysphoria: A review of the literature. International Review of Psychiatry 2016;28(1):44-57. - Fuss J, Auer MK, Briken P. Gender dysphoria in children and adolescents: a review of recent research. Current Opinion in Psychiatry 2015;28(6):430-4. - Guzman-Parra J, Paulino-Matos P, de Diego-Otero Y, et al. Substance Use and Social Anxiety in Transsexual Individuals. Journal of Dual Diagnosis 2014;10(3):162-7. - Hoekzema E, Schagen SEE, Kreukels BPC, et al. Regional volumes and spatial volumetric distribution of gray matter in the gender dysphoric brain. Psychoneuroendocrinology 2015;55:59-71. - Hoffman B. An Overview of Depression among Transgender Women. Depression Research and Treatment 2014;2014:1-9. - Holt V, Skagerberg E, Dunsford M. Young people with features of gender dysphoria: Demographics and associated difficulties. Clinical Child Psychology and Psychiatry 2016;21(1):108-18. - Jacobs LA, Rachlin K, Erickson-Schroth L, Janssen A. Gender Dysphoria and Co-Occurring Autism Spectrum Disorders: Review, Case Examples, and Treatment Considerations. LGBT Health 2014;1(4):277-82. - Johnson L, Shipherd J, Walton HM. The psychologist’s role in transgender-specific care with U.S. veterans. Psychological Services 2016;13(1):69-77. - Kauth MR, Shipherd JC, Lindsay J, et al. Access to Care for Transgender Veterans in the Veterans Health Administration: 2006–2013. American Journal of Public Health 2014;104(S4):S532-S4. - Maddera JC. Batson in Transition: Prohibiting Peremptory Challenges on the Basis of Gender Identity or Expression. Columbia Law Review 2016;116(1):195-235. - Matza AR, Sloan CA, Kauth MR. Quality LGBT Health Education: A Review of Key Reports and Webinars. Clinical Psychology: Science & Practice 2015;22(2):127-44. - Obedin-Maliver J, Makadon HJ. Transgender men and pregnancy. Obstetric Medicine: The Medicine of Pregnancy 2016;9(1):4-8. - Olson J, Schrager SM, Belzer M, et al. Baseline Physiologic and Psychosocial Characteristics of Transgender Youth Seeking Care for Gender Dysphoria. Journal of Adolescent Health 2015;57(4):374-80. - Parco JE, Levy DA, Spears SR. Transgender Military Personnel in the Post-DADT Repeal Era: A Phenomenological Study. Armed Forces & Society 2014. - Rosky CJ. No Promo Hetero: Children's Right to be Queer. Cardozo Law Review 2013;35(2):425-510. - Shires DA, Jaffee K. Factors Associated with Health Care Discrimination Experiences among a National Sample of Female-to-Male Transgender Individuals. Health & Social Work 2015;40(2):134-41. - Skagerberg E, Davidson S, Carmichael P. Internalizing and Externalizing Behaviors in a Group of Young People with Gender Dysphoria. International Journal of Transgenderism 2013;14(3):105-12. - Skagerberg E, Parkinson R, Carmichael P. Self-Harming Thoughts and Behaviors in a Group of Children and Adolescents with Gender Dysphoria. International Journal of Transgenderism 2013;14(2):86-92. - Smith A. Stories of 0s: Transgender Women, Monstrous Bodies, and the Canadian Prison System. Dalhousie Journal of Legal Studies 2014;23:149-71. - Thompson D. Commentary on “Gender disorders in learning disabilities – a systematic review”. Tizard Learning Disability Review 2014;19(4):166-9. - Travis M. Accommodating Intersexuality in European Union Anti-Discrimination Law. European Law Journal 2015;21(2):180-99. - Veltman A, Chaimowitz G. Mental Health Care for People Who Identify as Lesbian, Gay, Bisexual, Transgender, and (or) Queer. Canadian Journal of Psychiatry. Revue Canadienne de Psychiatrie 2014;59(11):1-7. - Winograd W. The Wish to Be a Boy: Gender Dysphoria and Identity Confusion in a Self-Identified Transgender Adolescent. Psychoanalytic Social Work 2014;21(1-2):55-74. - Witcomb GL, Bouman WP, Brewin N, et al. Body Image Dissatisfaction and Eating-Related Psychopathology in Trans Individuals: A Matched Control Study. European Eating Disorders Review 2015;23(4):287-93. - Wylie K, Barrett J, Besser M, et al. Good Practice Guidelines for the Assessment and Treatment of Adults with Gender Dysphoria. Sexual and Relationship Therapy 2014;29(2):154-214. - Zucker KJ, Lawrence AA, Kreukels BPC. Gender Dysphoria in Adults. Annual Review of Clinical Psychology 2016;12(1):217-47. |
| --- |
