# Supplementary material for: What Is Gender Dysphoria? A Critical Systematic Narrative Review
Source: Transgend Health. 2018 Nov 1;3(1):159–69. doi: 10.1089/trgh.2018.0014 (PMC6225591; doi:10.1089/trgh.2018.0014)
Supplement: Supplemental data [file Supp_Table6.docx]

Supplementary Table S6. Diagnosis of GD will reduce stigma

| - Barry KM, Farrell B, Levi JL, Vanguri N. A Bare Desire to Harm: Transgender People and the Equal Protection Clause. Boston College Law Review 2016;57(507):507-82. - Beek TF, Cohen-Kettenis PT, Kreukels BPC. Gender incongruence/gender dysphoria and its classification history. International Review of Psychiatry 2016;28(1):5-12. - Collazo A, Austin A, Craig SL. Facilitating Transition Among Transgender Clients: Components of Effective Clinical Practice. Clinical Social Work Journal 2013;41(3):228-37. - Kalra G, Tandon A, Sathyanarayana Rao TS. Sexual disorders in Asians: A review. Asian Journal of Psychiatry 2014;7:80-2. - Kelly F. Australian children living with gender dysphoria: does the Family Court have a role to play? Journal of law and medicine 2014;22(1):105-20. - Roberts TK, Fantz CR. Barriers to quality health care for the transgender population. Clinical Biochemistry 2014;47(10–11):983-7. - Rosky CJ. No Promo Hetero: Children's Right to be Queer. Cardozo Law Review 2013;35(2):425-510. - Selekman J, Diefenbeck C. The New DSM-5 and Its Impact on the Mental Health Care of Children. Journal of Pediatric Nursing 2014;29(5):442-50. - Shumer DE, Nokoff NJ, Spack NP. Advances in the Care of Transgender Children and Adolescents. Advances in Pediatrics 2016;63(1):79-102. - Toscano ME, Maynard E. Understanding the Link: “Homosexuality,” Gender Identity, and the DSM. Journal of LGBT Issues in Counseling 2014;8(3):248-63. - Washburn M. Five Things Social Workers Should Know about the DSM-5. Social Work 2013;58(4):373-6. |
| --- |
