# Supplementary material for: What Is Gender Dysphoria? A Critical Systematic Narrative Review
Source: Transgend Health. 2018 Nov 1;3(1):159–69. doi: 10.1089/trgh.2018.0014 (PMC6225591; doi:10.1089/trgh.2018.0014)
Supplement: Supplemental data [file Supp_Table7.docx]

Supplementary Table S7. Ensure Clinical Care

| - Aiken J. Promoting an Integrated Approach to Ensuring Access to Gender Incongruent Health Care. Berkeley Journal of Gender, Law & Justice 2016;Winter. - Armand H MA. Pubertal Suppression and Professional Obligations: May a Pediatric Endocrinologist Refuse to Treat an Adolescent With Gender Dysphoria? The American Journal of Bioethics 2014;14(1):43-6. - Bailey M. Transgender Workplace Discrimination in the Age of Gender Dysphoria and EDNA. Law & Psychology Review 2014;38:193-210. - Beek TF, Cohen-Kettenis PT, Kreukels BPC. Gender incongruence/gender dysphoria and its classification history. International Review of Psychiatry 2016;28(1):5-12. - Bendlin S. Gender Dysphoria in the Jailhouse: A Constitutional Right to Hormone Therapy? Cleveland State Law Review 2013;61(4):957-82. - Bockting WO. Vulnerability and Resilience Among Gender-Nonconforming Children and Adolescents: Mental Health Professionals Have a Key Role to Play. Journal of the American Academy of Child & Adolescent Psychiatry 2016;55(6):441-3. - Bouman WP, Richards C. Diagnostic and Treatment Issues for People with Gender Dysphoria in the United Kingdom. Sexual and Relationship Therapy 2013;28(3):165-71. - Campbell MM, Artz L, Stein DJ. Sexual disorders in DSM-5 and ICD-11: a conceptual framework. Current Opinion in Psychiatry 2015;28(6):435-9. - Castañeda C. Developing gender: The medical treatment of transgender young people. Social Science & Medicine 2015;143:262-70. - Corbett K, Dimen M, Goldner V, Harris A. Talking Sex, Talking Gender—A Roundtable. Studies in Gender and Sexuality 2014;15(4):295-317. - Daly TTW. Gender Dysphoria and the Ethics of Transsexual(i.e. Gender Reassignment) Surgery. Ethics & Medicine: An International Journal of Bioethics 2016;32(1):39-53. - Davy Z. The DSM-5 and the Politics of Diagnosing Transpeople. Archives of Sexual Behavior 2015;44(5):1165-76. - Drescher J. Queer diagnoses revisited: The past and future of homosexuality and gender diagnoses in DSM and ICD. International Review of Psychiatry 2015:1-10. - Gonzalez-Salzberg DA. The Accepted Transsexual and the Absent Transgender: A Queer Reading of the Regulation of Sex/Gender by the European Court of Human Rights, The. American University International Law Review 2013;29(4):797-829. - Güldenring A. A critical view of transgender health care in Germany: Psychopathologizing gender identity – Symptom of ‘disordered’ psychiatric/psychological diagnostics? International Review of Psychiatry 2015;27(5):427-34. - Johnson L, Shipherd J, Walton HM. The psychologist’s role in transgender-specific care with U.S. veterans. Psychological Services 2016;13(1):69-77. - Kelly F. Australian children living with gender dysphoria: does the Family Court have a role to play? Journal of law and medicine 2014;22(1):105-20. - Kon AA. Transgender Children and Adolescents. The American Journal of Bioethics 2014;14(1):48-50. - Kraus C. Classifying Intersex in DSM-5: Critical Reflections on Gender Dysphoria. Archives of Sexual Behavior 2015;44(5):1147-63. - Lev AI. Gender Dysphoria: Two Steps Forward, One Step Back. Clinical Social Work Journal 2013;41(3):288-96. - Levine DA, Braverman PK, Adelman WP, et al. Office-Based Care for Lesbian, Gay, Bisexual, Transgender, and Questioning Youth. Pediatrics 2013;132(1):198-203. - Meriggiola MC, Gava G. Endocrine care of transpeople part I. A review of cross-sex hormonal treatments, outcomes and adverse effects in transmen. Clinical Endocrinology 2015;83(5):597-606. - Riggs DW, Coleman K, Due C. Healthcare experiences of gender diverse Australians: a mixed-methods, self-report survey. BMC Public Health 2014;14(1):1-5. - Roberts TK, Fantz CR. Barriers to quality health care for the transgender population. Clinical Biochemistry 2014;47(10–11):983-7. - Shires DA, Jaffee K. Factors Associated with Health Care Discrimination Experiences among a National Sample of Female-to-Male Transgender Individuals. Health & Social Work 2015;40(2):134-41. - Smith A. Stories of 0s: Transgender Women, Monstrous Bodies, and the Canadian Prison System. Dalhousie Journal of Legal Studies 2014;23:149-71. - Toscano ME, Maynard E. Understanding the Link: “Homosexuality,” Gender Identity, and the DSM. Journal of LGBT Issues in Counseling 2014;8(3):248-63. - Veltman A, Chaimowitz G. Mental Health Care for People Who Identify as Lesbian, Gay, Bisexual, Transgender, and (or) Queer. Canadian Journal of Psychiatry. Revue Canadienne de Psychiatrie 2014;59(11):1-7. - White Hughto JM, Reisner SL, Pachankis JE. Transgender stigma and health: A critical review of stigma determinants, mechanisms, and interventions. Social Science & Medicine 2015;147:222-31. |
| --- |
