# Supplementary material for: What Is Gender Dysphoria? A Critical Systematic Narrative Review
Source: Transgend Health. 2018 Nov 1;3(1):159–69. doi: 10.1089/trgh.2018.0014 (PMC6225591; doi:10.1089/trgh.2018.0014)
Supplement: Supplemental data [file Supp_Table8.docx]

Supplementary Table S8. Post-transition specifier

| - Barry KM, Farrell B, Levi JL, Vanguri N. A Bare Desire to Harm: Transgender People and the Equal Protection Clause. Boston College Law Review 2016;57(507):507-82. - Corbett K, Dimen M, Goldner V, Harris A. Talking Sex, Talking Gender—A Roundtable. Studies in Gender and Sexuality 2014;15(4):295-317. - Drescher J. Controversies in Gender Diagnoses. LGBT Health 2013;1(1):10-4. - Drescher J. Queer diagnoses revisited: The past and future of homosexuality and gender diagnoses in DSM and ICD. International Review of Psychiatry 2015:1-10. - Firth MT. Childhood abuse, depressive vulnerability and gender dysphoria: Part 2. Counselling & Psychotherapy Research 2015;15(2):98-108. - Johnson L, Shipherd J, Walton HM. The psychologist’s role in transgender-specific care with U.S. veterans. Psychological Services 2016;13(1):69-77. - Toscano ME, Maynard E. Understanding the Link: “Homosexuality,” Gender Identity, and the DSM. Journal of LGBT Issues in Counseling 2014;8(3):248-63. - Zucker KJ, Lawrence AA, Kreukels BPC. Gender Dysphoria in Adults. Annual Review of Clinical Psychology 2016;12(1):217-47. |
| --- |
