# Supplementary material for: What Is Gender Dysphoria? A Critical Systematic Narrative Review
Source: Transgend Health. 2018 Nov 1;3(1):159–69. doi: 10.1089/trgh.2018.0014 (PMC6225591; doi:10.1089/trgh.2018.0014)
Supplement: Supplemental data [file Supp_Table9.docx]

Supplementary Table S9. Prevalence of GD

| - *Aitken M, Steensma TD, Blanchard R, et al. Evidence for an Altered Sex Ratio in Clinic-Referred Adolescents with Gender Dysphoria. The Journal of Sexual Medicine 2015;12(3):756-63. - *Arcelus J, Bouman WP, Van Den Noortgate W, et al. Systematic review and meta-analysis of prevalence studies in transsexualism. European Psychiatry 2015;30(6):807-15. - Bell F. Children with gender dysphoria and the jurisdiction of the Family Court. University of New South Wales Law Journal 2015;38(2):426-54. - Bouman WP, de Vries ALC, T’Sjoen G. Gender Dysphoria and Gender Incongruence: An evolving inter-disciplinary field. International Review of Psychiatry 2016;28(1):1-4. - *Busari AO. Bolstering Self-Esteem as Intervention Technique in the Management of Symptoms of Gender Identity Disorder among Adolescents. Gender & Behaviour 2013;11(2):5535-45. - *Capetillo-Ventura NC, Jalil-Pérez SI, Motilla-Negrete K. Gender dysphoria: An overview. Medicina Universitaria 2015;17(66):53-8. - *Chen M, Fuqua J, Eugster EA. Characteristics of Referrals for Gender Dysphoria Over a 13-Year Period. Journal of Adolescent Health 2016;58(3):369-71. - *Collin L, Reisner SL, Tangpricha V, Goodman M. Prevalence of Transgender Depends on the “Case” Definition: A Systematic Review. The Journal of Sexual Medicine 2016;13(4):613-26. - Ehsanzadeh P, Raza S, Haq Z. A New Perspective on Gender Dysphoria and Repetitive Sex Reassignment Surgeries: A Case Report. The Primary Care Companion for CNS Disorders 2014;16(2):PCC.13l01608. - Fitzgibbons RP. Transsexual attractions and sexual reassignment surgery: Risks and potential risks. The Linacre Quarterly 2015;82(4):337-50. - *Hardy TLD, Boliek CA, Wells K, Rieger JM. The ICF and Male-to-Female Transsexual Communication. International Journal of Transgenderism 2013;14(4):196-208. - *Heylens G, Verroken C, De Cock S, et al. Effects of Different Steps in Gender Reassignment Therapy on Psychopathology: A Prospective Study of Persons with a Gender Identity Disorder. The Journal of Sexual Medicine 2014;11(1):119-26. - Jones BA, Haycraft E, Murjan S, Arcelus J. Body dissatisfaction and disordered eating in trans people: A systematic review of the literature. International Review of Psychiatry 2016;28(1):81-94. - Judge C, O’Donovan C, Callaghan G, et al. Gender Dysphoria – Prevalence and Co-Morbidities in an Irish Adult Population. Frontiers in Endocrinology 2014;5(87). - *Kaltiala-Heino R, Sumia M, Työläjärvi M, Lindberg N. Two years of gender identity service for minors: overrepresentation of natal girls with severe problems in adolescent development. Child and Adolescent Psychiatry and Mental Health 2015;1(9):1-9. - Kauth MR, Shipherd JC, Lindsay J, et al. Access to Care for Transgender Veterans in the Veterans Health Administration: 2006–2013. American Journal of Public Health 2014;104(S4):S532-S4. - *Kristensen ZE, Broome MR. Autistic Traits in an Internet Sample of Gender Variant UK Adults. International Journal of Transgenderism 2015;16(4):234-45. - Levin D, Does changing gender make children happier? Archives of Disease in Childhood 2016;101(5):460. - *Majumder A, Sanyal D. Outcome and preferences in female-to-male subjects with gender dysphoria: Experience from Eastern India. Indian Journal of Endocrinology & Metabolism 2016;20(3):308-11. - *Mazaheri Meybodi A, Hajebi A, Ghanbari Jolfaei A. Psychiatric Axis I Comorbidities among Patients with Gender Dysphoria. Psychiatry Journal 2014;2014:5. - *Merryfeather L, Bruce A. The Invisibility of Gender Diversity: Understanding Transgender and Transsexuality in Nursing Literature. Nursing Forum 2014;49(2):110-23. - *Motmans J, Ponnet K, De Cuypere G. Sociodemographic Characteristics of Trans Persons in Belgium: A Secondary Data Analysis of Medical, State, and Social Data. Archives of Sexual Behavior 2015;44(5):1289-99. - Nakachi Y, Iseki M, Yokoo T, et al. Gene Expression Profile of the Neonatal Female Mouse Brain After Administration of Testosterone Propionate. The Journal of Sexual Medicine 2015;12(4):887-96. - *Nieder TO, Elaut E, Richards C, Dekker A. Sexual orientation of trans adults is not linked to outcome of transition-related health care, but worth asking. International Review of Psychiatry 2016;28(1):103-11. - Olson J, Schrager SM, Belzer M, et al. Baseline Physiologic and Psychosocial Characteristics of Transgender Youth Seeking Care for Gender Dysphoria. Journal of Adolescent Health 2015;57(4):374-80. - *Pasterski V, Gilligan L, Curtis R. Traits of Autism Spectrum Disorders in Adults with Gender Dysphoria. Archives of Sexual Behavior 2014;43(2):387-93. - *Pasterski V, Zucker KJ, Hindmarsh PC, et al. Increased Cross-Gender Identification Independent of Gender Role Behavior in Girls with Congenital Adrenal Hyperplasia: Results from a Standardized Assessment of 4- to 11-Year-Old Children. Archives of Sexual Behavior 2015;44(5):1363-75. - Raigosa M, Avvedimento S, Yoon TS, et al. Male-to-Female Genital Reassignment Surgery: A Retrospective Review of Surgical Technique and Complications in 60 Patients. The Journal of Sexual Medicine 2015;12(8):1837-45. - Rezwan N, Basit AA, Andrews H. Bilateral ureteric obstruction: an unusual complication of male-to-female gender reassignment surgery. BMJ Case Reports 2014;2014:bcr2014204894. - Richards C, Bouman WP, Seal L, et al. Non-binary or genderqueer genders. International Review of Psychiatry 2016;28(1):95-102. - Ristori J, Steensma TD. Gender dysphoria in childhood. International Review of Psychiatry 2016;28(1):13-20. - Roberts TK, Fantz CR. Barriers to quality health care for the transgender population. Clinical Biochemistry 2014;47(10–11):983-7. - *Sanyal D, Majumder A. Presentation of gender dysphoria: A perspective from Eastern India. Indian Journal of Endocrinology & Metabolism 2016;20(1):129-33. - Schmidt L, Levine R. Psychological Outcomes and Reproductive Issues Among Gender Dysphoric Individuals. Endocrinology and Metabolism Clinics of North America 2015;44(4):773-85. - *Schneider F, Neuhaus N, Wistuba J, et al. Testicular Functions and Clinical Characterization of Patients with Gender Dysphoria (GD) Undergoing Sex Reassignment Surgery (SRS). The Journal of Sexual Medicine 2015;12(11):2190-200. - *Schwarz K, Fontanari AMV, Mueller A, et al. Transsexual Voice Questionnaire for Male-to-female Brazilian Transsexual People. Journal of Voice 2017;31(1):e15-e20. - Seal LJ. A review of the physical and metabolic effects of cross-sex hormonal therapy in the treatment of gender dysphoria. Annals of Clinical Biochemistry: An international journal of biochemistry and laboratory medicine 2016;53(1):10-20. - Shumer DE, Nokoff NJ, Spack NP. Advances in the Care of Transgender Children and Adolescents. Advances in Pediatrics 2016;63(1):79-102. - Simons L, Leibowitz S, Hidalgo M. Understanding Gender Variance in Children and Adolescents. Pediatric Annals 2014;43(6):e126-e31. - *Smith MK, Mathews B. Treatment for gender dysphoria in children: the new legal, ethical and clinical landscape. The Medical Journal of Australia 2015;202(2):102-4. - *Södersten M, Nygren U, Hertegård S, Dhejne C. Interdisciplinary Program in Sweden Related to Transgender Voice. SIG 3 Perspectives on Voice and Voice Disorders 2015;25(2):87-97. - Van Caenegem E, Wierckx K, Elaut E, et al. Prevalence of Gender Nonconformity in Flanders, Belgium. Archives of Sexual Behavior 2015;44(5):1281-7. - *Van Der Miesen AIR, Hurley H, De Vries ALC. Gender dysphoria and autism spectrum disorder: A narrative review. International Review of Psychiatry 2016;28(1):70-80. - VanderLaan DP, Leef JH, Wood H, et al. Autism Spectrum Disorder Risk Factors and Autistic Traits in Gender Dysphoric Children. Journal of Autism and Developmental Disorders 2015;45(6):1742-50. - Wang LC, Poppas DP. Surgical outcomes and complications of reconstructive surgery in the female congenital adrenal hyperplasia patient: What every endocrinologist should know. The Journal of Steroid Biochemistry and Molecular Biology 2017;165(Pt A):137-44. - *Wangjiraniran B, Selvaggi G, Chokrungvaranont P, et al. Male-to-female vaginoplasty: Preecha’s surgical technique. Journal of Plastic Surgery and Hand Surgery 2015;49(3):153-9. - Zucker KJ, Seto MC. Gender dysphoria and paraphilic sexual disorders. In: Thapar A, Pine DS, Leckman JF, et al., eds. Rutter's Child and Adolescent Psychiatry. Chichester: John Wiley & Sons, 2015. - *Zucker KJ, Lawrence AA, Kreukels BPC. Gender Dysphoria in Adults. Annual Review of Clinical Psychology 2016;12(1):217-47. |
| --- |
