# Supplementary material for: What Is Gender Dysphoria? A Critical Systematic Narrative Review
Source: Transgend Health. 2018 Nov 1;3(1):159–69. doi: 10.1089/trgh.2018.0014 (PMC6225591; doi:10.1089/trgh.2018.0014)
Supplement: Supplemental data [file Supp_Table10.docx]

Supplementary Table S10. Biomedical causes of GD

| - Ahlam A, Al Sinawi H, Al Alawi M. Gender dysphoria in an Omani female. International Journal of Nutrition, Pharmacology, Neurological Diseases 2016;6(2):97-9. - Barry KM, Farrell B, Levi JL, Vanguri N. A Bare Desire to Harm: Transgender People and the Equal Protection Clause. Boston College Law Review 2016;57(507):507-82. - Bockting WO. Vulnerability and Resilience Among Gender-Nonconforming Children and Adolescents: Mental Health Professionals Have a Key Role to Play. Journal of the American Academy of Child & Adolescent Psychiatry 2016;55(6):441-3. - Bockting W, Coleman E, Deutsch MB, et al. Adult development and quality of life of transgender and gender nonconforming people. Current Opinion in Endocrinology, Diabetes and Obesity 2016;23(2):188-97. - Bouman WP, Richards C. Diagnostic and Treatment Issues for People with Gender Dysphoria in the United Kingdom. Sexual and Relationship Therapy 2013;28(3):165-71. - Capetillo-Ventura NC, Jalil-Pérez SI, Motilla-Negrete K. Gender dysphoria: An overview. Medicina Universitaria 2015;17(66):53-8. - Majumder A, Sanyal D. Outcome and preferences in female-to-male subjects with gender dysphoria: Experience from Eastern India. Indian Journal of Endocrinology & Metabolism 2016;20(3):308-11. - Quam K. Unfinished Business of Repealing Don't Ask, Don't Tell: The Military's Unconstitutional Ban on Transgender Individuals. Utah Law Review 2015(3):721-41. - Shumer DE, Roberts AL, Reisner SL, et al. Brief Report: Autistic Traits in Mothers and Children Associated with Child’s Gender Nonconformity. Journal of Autism and Developmental Disorders 2015;45(5):1489-94. - Steensma TD, Kreukels BPC, de Vries ALC, Cohen-Kettenis PT. Gender identity development in adolescence. Hormones and Behavior 2013;64(2):288-97. - Turan Ş, Poyraz CA, Duran A. Prolonged anorexia nervosa associated with female-to-male gender dysphoria: A case report. Eating Behaviors 2015;18:54-6. - Zucker KJ, Seto MC. Gender dysphoria and paraphilic sexual disorders. In: Thapar A, Pine DS, Leckman JF, et al., eds. Rutter's Child and Adolescent Psychiatry. Chichester: John Wiley & Sons, 2015. - Zucker KJ, Lawrence AA, Kreukels BPC. Gender Dysphoria in Adults. Annual Review of Clinical Psychology 2016;12(1):217-47. |
| --- |
