# Supplementary material for: What Is Gender Dysphoria? A Critical Systematic Narrative Review
Source: Transgend Health. 2018 Nov 1;3(1):159–69. doi: 10.1089/trgh.2018.0014 (PMC6225591; doi:10.1089/trgh.2018.0014)
Supplement: Supplemental data [file Supp_Table11.docx]

Supplementary Table S11. No biological etiology of GD

| - Auer MK, Hellweg R, Briken P, et al. Serum brain-derived neurotrophic factor (BDNF) is not regulated by testosterone in transmen. Biology of Sex Differences 2016;7(1):1-6. - Aydin D, Buk LJ, Partoft S, et al. Transgender Surgery in Denmark From 1994 to 2015: 20-Year Follow-Up Study. The Journal of Sexual Medicine 2016;13(4):720-5. - Beek TF, Cohen-Kettenis PT, Kreukels BPC. Gender incongruence/gender dysphoria and its classification history. International Review of Psychiatry 2016;28(1):5-12. - Bendlin S. Gender Dysphoria in the Jailhouse: A Constitutional Right to Hormone Therapy? Cleveland State Law Review 2013;61(4):957-82. - Bouman WP, Richards C, Addinall RM, et al. Yes and yes again: are standards of care which require two referrals for genital reconstructive surgery ethical? Sexual and Relationship Therapy 2014;29(4):377-89. - Cohen-Kettenis PT, Klink D. Adolescents with gender dysphoria. Best Practice & Research Clinical Endocrinology & Metabolism 2015;29(3):485-95. - Colizzi M. Attachment Styles in Transsexual Patients and Clinical and Nonclinical Control Groups: A Response. The Journal of Sexual Medicine 2014;11(4):1094-5. - Fernández R, Esteva I, Gómez-Gil E, et al. Association Study of ERβ, AR, and CYP19A1 Genes and MtF Transsexualism. The Journal of Sexual Medicine 2014;11(12):2986-94. - Fuss J, Biedermann SV, Stalla GK, Auer MK. On the quest for a biomechanism of transsexualism: Is there a role for BDNF? Journal of Psychiatric Research 2013;47(12):2015-7. - Gooren LJ, Giltay EJ. Men and women, so different, so similar: observations from cross-sex hormone treatment of transsexual subjects. Andrologia 2014;46(5):570-5. - Hardy TLD, Boliek CA, Wells K, Rieger JM. The ICF and Male-to-Female Transsexual Communication. International Journal of Transgenderism 2013;14(4):196-208. - Junger J, Habel U, Bröhr S, et al. More than Just Two Sexes: The Neural Correlates of Voice Gender Perception in Gender Dysphoria. PLoS ONE 2014;9(11):1-12. - Steensma TD, Kreukels BPC, de Vries ALC, Cohen-Kettenis PT. Gender identity development in adolescence. Hormones and Behavior 2013;64(2):288-97. - Zucker KJ, Lawrence AA, Kreukels BPC. Gender Dysphoria in Adults. Annual Review of Clinical Psychology 2016;12(1):217-47. |
| --- |
