# Supplementary material for: What Is Gender Dysphoria? A Critical Systematic Narrative Review
Source: Transgend Health. 2018 Nov 1;3(1):159–69. doi: 10.1089/trgh.2018.0014 (PMC6225591; doi:10.1089/trgh.2018.0014)
Supplement: Supplemental data [file Supp_Table12.docx]

Supplementary Table S12. Research populations

| - Arcelus J, Bouman WP, Van Den Noortgate W, et al. Systematic review and meta-analysis of prevalence studies in transsexualism. European Psychiatry 2015;30(6):807-15. - Auer MK, Hellweg R, Briken P, et al. Serum brain-derived neurotrophic factor (BDNF) is not regulated by testosterone in transmen. Biology of Sex Differences 2016;7(1):1-6. - Azul D. Transmasculine people's vocal situations: a critical review of gender-related discourses and empirical data. International Journal of Language & Communication Disorders 2015;50(1):31-47. - Bartolucci C, Gómez-Gil E, Salamero M, et al. Sexual Quality of Life in Gender-Dysphoric Adults before Genital Sex Reassignment Surgery. The Journal of Sexual Medicine 2015;12(1):180-8. - Becerra-Fernández A, Pérez-López G, Menacho Román M, et al. Prevalence of hyperandrogenism and polycystic ovary syndrome in female to male transsexuals. Endocrinología y Nutrición (English Edition) 2014;61(7):351-8. - Becker I, Nieder TO, Cerwenka S, et al. Body Image in Young Gender Dysphoric Adults: A European Multi-Center Study. Archives of Sexual Behavior 2016;45(3):559-74. - Bendlin S. Gender Dysphoria in the Jailhouse: A Constitutional Right to Hormone Therapy? Cleveland State Law Review 2013;61(4):957-82. - Bockting WO, Miner MH, Swinburne Romine RE, et al. Stigma, Mental Health, and Resilience in an Online Sample of the US Transgender Population. American Journal of Public Health 2013;103(5):943-51. - Bodoin EM, Byrd CT, Adler RK. The Clinical Profile of the Male-to-Female Transgender Person of the 21st Century. Contemporary Issues in Communication Science & Disorders 2014;41:39-54. - Bouman WP, Davey A, Meyer C, et al. Predictors of psychological well-being among treatment seeking transgender individuals. Sexual and Relationship Therapy 2016:1-17. - Brown GR. Breast Cancer in Transgender Veterans: A Ten-Case Series. LGBT Health 2015;2(1):77-80. - Burke SM, Kreukels BPC, Cohen-Kettenis PT, et al. Male-typical visuospatial functioning in gynephilic girls with gender dysphoria - organizational and activational effects of testosterone. Journal of psychiatry & neuroscience : JPN 2016;41(6):395-404. - Cerwenka S, Nieder TO, Cohen-Kettenis P, et al. Sexual Behavior of Gender-Dysphoric Individuals Before Gender-Confirming Interventions: A European Multicenter Study. Journal of Sex & Marital Therapy 2014;40(5):457-71. - Chen M, Fuqua J, Eugster EA. Characteristics of Referrals for Gender Dysphoria Over a 13-Year Period. Journal of Adolescent Health 2016;58(3):369-71. - Ciocca G, Limoncin E, Cellerino A, et al. Gender Identity Rather Than Sexual Orientation Impacts on Facial Preferences. The Journal of Sexual Medicine 2014;11(10):2500-7. - Claes L, Bouman WP, Witcomb G, et al. Non-Suicidal Self-Injury in Trans People: Associations with Psychological Symptoms, Victimization, Interpersonal Functioning, and Perceived Social Support. The Journal of Sexual Medicine 2015;12(1):168-79. - Colizzi M, Costa R, Scaramuzzi F, et al. Concomitant psychiatric problems and hormonal treatment induced metabolic syndrome in gender dysphoria individuals: A 2 year follow-up study. Journal of Psychosomatic Research 2015;78(4):399-406. - Davey A, Bouman WP, Meyer C, Arcelus J. Interpersonal Functioning Among Treatment-Seeking Trans Individuals. Journal of Clinical Psychology 2015;71(12):1173-85. - Davey A, Arcelus J, Meyer C, Bouman WP. Self-injury among trans individuals and matched controls: prevalence and associated factors. Health and Social Care in the Community 2016;24(4):485-94. - de Vries ALC, McGuire JK, Steensma TD, et al. Young Adult Psychological Outcome After Puberty Suppression and Gender Reassignment. Pediatrics 2014;134(4):696-704. - de Vries ALC, Steensma TD, Cohen-Kettenis PT, et al. Poor peer relations predict parent- and self-reported behavioral and emotional problems of adolescents with gender dysphoria: a cross-national, cross-clinic comparative analysis. European Child & Adolescent Psychiatry 2016;25(6):579-88. - Di Ceglie D, Skagerberg E, Baron-Cohen S, Auyeung B, 16 (6). Empathising and systemising in adolescents with gender dysphoria. Opticon 1826 2014;16(6):1-8. - Fisher AD, Castellini G, Bandini E, et al. Cross-Sex Hormonal Treatment and Body Uneasiness in Individuals with Gender Dysphoria. The Journal of Sexual Medicine 2014;11(3):709-19. - Fisher AD, Castellini G, Casale H, et al. Hypersexuality, Paraphilic Behaviors, and Gender Dysphoria in Individuals with Klinefelter's Syndrome. The Journal of Sexual Medicine 2015;12(12):2413-24. - Gava G, Cerpolini S, Martelli V, et al. Cyproterone acetate vs leuprolide acetate in combination with transdermal oestradiol in transwomen: a comparison of safety and effectiveness. Clinical Endocrinology 2016;85(2):239-46. - Heylens G, Verroken C, De Cock S, et al. Effects of Different Steps in Gender Reassignment Therapy on Psychopathology: A Prospective Study of Persons with a Gender Identity Disorder. The Journal of Sexual Medicine 2014;11(1):119-26. - Higuchi T, Holmdahl G, Kaefer M, et al. International Consultation on Urological Diseases: Congenital Anomalies of the Genitalia in Adolescence. Urology 2016;94:288-310. - Hoekzema E, Schagen SEE, Kreukels BPC, et al. Regional volumes and spatial volumetric distribution of gray matter in the gender dysphoric brain. Psychoneuroendocrinology 2015;55:59-71. - Holt V, Skagerberg E, Dunsford M. Young people with features of gender dysphoria: Demographics and associated difficulties. Clinical Child Psychology and Psychiatry 2016;21(1):108-18. - Jones BA, Haycraft E, Murjan S, Arcelus J. Body dissatisfaction and disordered eating in trans people: A systematic review of the literature. International Review of Psychiatry 2016;28(1):81-94. - Junger J, Habel U, Bröhr S, et al. More than Just Two Sexes: The Neural Correlates of Voice Gender Perception in Gender Dysphoria. PLoS ONE 2014;9(11):1-12. - Kaltiala-Heino R, Sumia M, Työläjärvi M, Lindberg N. Two years of gender identity service for minors: overrepresentation of natal girls with severe problems in adolescent development. Child and Adolescent Psychiatry and Mental Health 2015;1(9):1-9. - Leibowitz S, de Vries ALC. Gender dysphoria in adolescence. International Review of Psychiatry 2016;28(1):21-35. - Levin D. Does changing gender make children happier? Archives of Disease in Childhood 2016;101(5):460. - Li F, Rendall D, Vasey PL, et al. The development of sex/gender-specific /s/ and its relationship to gender identity in children and adolescents. Journal of Phonetics 2016;57:59-70. - McCann E, Sharek D. Mental Health Needs of People Who Identify as Transgender: A Review of the Literature. Archives of Psychiatric Nursing 2016;30(2):280-5. - Ni H-C, Gau SS-F. Co-occurrence of attention-deficit hyperactivity disorder symptoms with other psychopathology in young adults: parenting style as a moderator. Comprehensive Psychiatry 2015;57:85-96. - Olson J, Schrager SM, Belzer M, et al. Baseline Physiologic and Psychosocial Characteristics of Transgender Youth Seeking Care for Gender Dysphoria. Journal of Adolescent Health 2015;57(4):374-80. - Petricevic L, Kaufmann U, Domig KJ, et al. Rectal Lactobacillus Species and Their Influence on the Vaginal Microflora: A Model of Male-to-Female Transsexual Women. The Journal of Sexual Medicine 2014;11(11):2738-43. - Rabito-Alcón MF, Rodríguez-Molina JM. Satisfaction with life and psychological well-being in people with gender dysphoria. Actas espanolas de psiquiatria 2016;44(2):47-54. - Salgado CJ, Nugent AG, Moody AM, et al. Immediate pedicled gracilis flap in radial forearm flap phalloplasty for transgender male patients to reduce urinary fistula. Journal of Plastic, Reconstructive & Aesthetic Surgery 2016;69(11):1551-7. - Sanyal D, Majumder A. Presentation of gender dysphoria: A perspective from Eastern India. Indian Journal of Endocrinology & Metabolism 2016;20(1):129-33. - Smith ES, Junger J, Derntl B, Habel U. The transsexual brain – A review of findings on the neural basis of transsexualism. Neuroscience & Biobehavioral Reviews 2015;59:251-66. - Swann WB, Gómez Á, Vázquez A, et al. Fusion with the Cross-Gender Group Predicts Genital Sex Reassignment Surgery. Archives of Sexual Behavior 2015;44(5):1313-8. - Tack LJW, Craen M, Dhondt K, et al. Consecutive lynestrenol and cross-sex hormone treatment in biological female adolescents with gender dysphoria: a retrospective analysis. Biology of Sex Differences 2016;7(1):1-11. - Toffoletto S, Lanzenberger R, Gingnell M, et al. Emotional and cognitive functional imaging of estrogen and progesterone effects in the female human brain: A systematic review. Psychoneuroendocrinology 2014;50:28-52. - Turan Ş, Poyraz CA, Duran A. Prolonged anorexia nervosa associated with female-to-male gender dysphoria: A case report. Eating Behaviors 2015;18:54-6. - Turan Ş, Poyraz CA, Öcek Baş T, et al. Affective temperaments in subjects with female-to-male gender dysphoria. Journal of Affective Disorders 2015;176:61-4. - van de Grift TC, Cohen-Kettenis PT, Elaut E, et al. A network analysis of body satisfaction of people with gender dysphoria. Body Image 2016;17:184-90. - van de Grift TC, Cohen-Kettenis PT, Steensma TD, et al. Body Satisfaction and Physical Appearance in Gender Dysphoria. Archives of Sexual Behavior 2016;45(3):575-85. - Van Der Miesen AIR, Hurley H, De Vries ALC. Gender dysphoria and autism spectrum disorder: A narrative review. International Review of Psychiatry 2016;28(1):70-80. - VanderLaan DP, Blanchard R, Wood H, Zucker KJ. Birth Order and Sibling Sex Ratio of Children and Adolescents Referred to a Gender Identity Service. PloS One 2014;9(3):e90257. - Wood E, Halder N. Gender disorders in learning disability – a systematic review. Tizard Learning Disability Review 2014;19(4):158-65. |
| --- |
