# Supplementary material for: What Is Gender Dysphoria? A Critical Systematic Narrative Review
Source: Transgend Health. 2018 Nov 1;3(1):159–69. doi: 10.1089/trgh.2018.0014 (PMC6225591; doi:10.1089/trgh.2018.0014)
Supplement: Supplemental data [file Supp_Table13.docx]

Supplementary Table S13. Extreme GD

| - Ahmad S, Barrett J, Beaini AY, et al. Gender dysphoria services: a guide for general practitioners and other healthcare staff. Sexual and Relationship Therapy 2013;28(3):172-85. - Bui HN, Schagen SEE, Klink DT, et al. Salivary testosterone in female-to-male transgender adolescents during treatment with intra-muscular injectable testosterone esters. Steroids 2013;78(1):91-5. - Capetillo-Ventura NC, Jalil-Pérez SI, Motilla-Negrete K. Gender dysphoria: An overview. Medicina Universitaria 2015;17(66):53-8. - Cohen-Kettenis PT, Klink D. Adolescents with gender dysphoria. Best Practice & Research Clinical Endocrinology & Metabolism 2015;29(3):485-95. - Esteva de Antonio I, Gómez-Gil E, Group aG. Coordination of healthcare for transsexual persons: a multidisciplinary approach. Current Opinion in Endocrinology, Diabetes and Obesity 2013;20(6):585-91. - Gómez-Gil E, Zubiaurre-Elorza L, Esteva de Antonio I, et al. Determinants of quality of life in Spanish transsexuals attending a gender unit before genital sex reassignment surgery. Quality of Life Research 2014;23(2):669-76. - Gooren LJ, Giltay EJ. Men and women, so different, so similar: observations from cross-sex hormone treatment of transsexual subjects. Andrologia 2014;46(5):570-5. - Gooren LJ, Kreukels B, Lapauw B, Giltay EJ. (Patho)physiology of cross-sex hormone administration to transsexual people: the potential impact of male-female genetic differences. Andrologia 2015;47(1):5-19. - Kelso T. Still Trapped in the U.S. Media’s Closet: Representations of Gender-Variant, Pre-Adolescent Children. Journal of Homosexuality 2015;62(8):1058-97. - Levine DA, Braverman PK, Adelman WP, et al. Office-Based Care for Lesbian, Gay, Bisexual, Transgender, and Questioning Youth. Pediatrics 2013;132(1):198-203. - Loverro G, Di Naro E, Caringella AM, et al. Prevalence of human papillomavirus infection in a clinic sample of transsexuals in Italy. Sexually Transmitted Infections 2016;92(1):67-9. - Masroor AS. Gender Identity Disorder is Not Simply Two in One. The International Medical Journal of Malaysia 2013;12(2). - Osborne CS, Lawrence AA. Male Prison Inmates With Gender Dysphoria: When Is Sex Reassignment Surgery Appropriate? Archives of Sexual Behavior 2016:1-15. - Schneider C, Cerwenka S, Nieder TO, et al. Measuring Gender Dysphoria: A Multicenter Examination and Comparison of the Utrecht Gender Dysphoria Scale and the Gender Identity/Gender Dysphoria Questionnaire for Adolescents and Adults. Archives of Sexual Behavior 2016;45(3):551-8. - Soleman RS, Schagen SEE, Veltman DJ, et al. Sex Differences in Verbal Fluency during Adolescence: A Functional Magnetic Resonance Imaging Study in Gender Dysphoric and Control Boys and Girls. The Journal of Sexual Medicine 2013;10(8):1969-77. - Vitelli R. Adult Male-to-Female Transsexualism A Clinical Existential-Phenomenological Inquiry. Journal of Phenomenological Psychology 2015;46(1):33-68. - Vrouenraets LJJJ, Fredriks AM, Hannema SE, et al. Early Medical Treatment of Children and Adolescents With Gender Dysphoria: An Empirical Ethical Study. Journal of Adolescent Health 2015;57(4):367-73. - Withers R. The seventh penis: towards effective psychoanalytic work with pre-surgical transsexuals. Journal of Analytical Psychology 2015;60(3):390-412. |
| --- |
