# Supplementary material for: What Is Gender Dysphoria? A Critical Systematic Narrative Review
Source: Transgend Health. 2018 Nov 1;3(1):159–69. doi: 10.1089/trgh.2018.0014 (PMC6225591; doi:10.1089/trgh.2018.0014)
Supplement: Supplemental data [file Supp_Table14.docx]

Supplementary Table S14. Differences in Sex Development and GD

| - Amaral RC, Inacio M, Brito VN, et al. Quality of life of patients with 46,XX and 46,XY disorders of sex development. Clinical Endocrinology 2015;82(2):159-64. - Anderson S. Disorders of Sexual Differentiation: Ethical Considerations Surrounding Early Cosmetic Genital Surgery. Pediatric Nursing 2015;41(4):176-86. - Auer MK, Fuss J, Stalla GK, Athanasoulia AP. Twenty years of endocrinologic treatment in transsexualism: analyzing the role of chromosomal analysis and hormonal profiling in the diagnostic work-up. Fertility and Sterility 2013;100(4):1103-10. - Beek TF, Cohen-Kettenis PT, Kreukels BPC. Gender incongruence/gender dysphoria and its classification history. International Review of Psychiatry 2016;28(1):5-12. - Berenbaum SA, Meyer-Bahlburg HFL. Gender development and sexuality in disorders of sex development. Hormone and metabolic research 2015;47(5):361-6. - Bermúdez de la Vega JA, Fernández-Cancio M, Bernal S, Audí L. Complete Androgen Insensitivity Syndrome Associated with Male Gender Identity or Female Precocious Puberty in the Same Family. Sexual Development 2015;9(2):75-9. - Costa MM, Lau E, Rodrigues P, et al. Gender Dysphoria in a Genetic Female with Non-Classical Congenital Adrenal Hyperplasia. Archives of Sexual Behavior 2016:1-4. - Diamond M, Garland J. Evidence regarding cosmetic and medically unnecessary surgery on infants. Journal of Pediatric Urology 2014;10(1):2-6. - Drescher J. Queer diagnoses revisited: The past and future of homosexuality and gender diagnoses in DSM and ICD. International Review of Psychiatry 2015:1-10. - Ekenze SO, Adiri CO, Igwilo IO, Onumaegbu OO. Virilized External Genitalia in Young Girls: Clinical Characteristics and Management Challenges in a Low-Resource Setting. Journal of Pediatric and Adolescent Gynecology 2014;27(1):6-9. - Fisher AD, Castellini G, Casale H, et al. Hypersexuality, Paraphilic Behaviors, and Gender Dysphoria in Individuals with Klinefelter's Syndrome. The Journal of Sexual Medicine 2015;12(12):2413-24. - Güldenring A. A critical view of transgender health care in Germany: Psychopathologizing gender identity – Symptom of ‘disordered’ psychiatric/psychological diagnostics? International Review of Psychiatry 2015;27(5):427-34. - Jürgensen M, Kleinemeier E, Lux A, et al. Psychosexual Development in Adolescents and Adults with Disorders of Sex Development—Results from the German Clinical Evaluation Study. The Journal of Sexual Medicine 2013;10(11):2703-14. - Jürgensen M, Lux A, Wien SB, et al. Health-related quality of life in children with disorders of sex development (DSD). European Journal of Pediatrics 2014;173(7):893-903. - Kanhere M, Fuqua J, Rink R, et al. Psychosexual development and quality of life outcomes in females with congenital adrenal hyperplasia. International Journal of Pediatric Endocrinology 2015;2015(1):1-9. - Khattab A, Yau M, Qamar A, et al. Long term outcomes in 46, XX adult patients with congenital adrenal hyperplasia reared as males. The Journal of Steroid Biochemistry and Molecular Biology 2016. - Kukreti P, Kandpal M, Jiloha RC. Mistaken gender identity in non-classical congenital adrenal hyperplasia. Indian Journal of Psychiatry 2014;56(2):182-4. - Lekarev O, Lin-Su K, Vogiatzi MG. Infertility and Reproductive Function in Patients with Congenital Adrenal Hyperplasia: Pathophysiology, Advances in Management, and Recent Outcomes. Endocrinology and Metabolism Clinics of North America 2015;44(4):705-22. - McCracken KA, Fallat ME. Transition from pediatric to adult surgery care for patients with disorders of sexual development. Seminars in Pediatric Surgery 2015;24(2):88-92. - McDonald E. Intersex people in Aotearoa New Zealand: The challenges for law and social policy: Part I: Critiquing gender normalising surgery. Victoria University. Wellington Law Review 2015;46:705-24. - Meyer-Bahlburg HFL. Psychoendocrinology of Congenital Adrenal Hyperplasia. In: Hammer MI, New O, Alan L, et al., eds. Genetic Steroid Disorders. San Diego: Academic Press, 2014. - Mouriquand PDE, Gorduza DB, Gay C-L, et al. Surgery in disorders of sex development (DSD) with a gender issue: If (why), when, and how? Journal of Pediatric Urology 2016;12(3):139-49. - Öcal G, Berberoğlu M, Sıklar Z, et al. Clinical Review of 95 Patients with 46,XX Disorders of Sex Development Based on the New Chicago Classification. Journal of Pediatric and Adolescent Gynecology 2015;28(1):6-11. - Parkinson J. Gender dysphoria in Asperger’s syndrome: a caution. Australasian Psychiatry 2014;22(1):84-5. - Pasterski V, Zucker KJ, Hindmarsh PC, et al. Increased Cross-Gender Identification Independent of Gender Role Behavior in Girls with Congenital Adrenal Hyperplasia: Results from a Standardized Assessment of 4- to 11-Year-Old Children. Archives of Sexual Behavior 2015;44(5):1363-75. - Renukanthan A, Quinton R, Turner B, et al. Kallmann syndrome patient with gender dysphoria, multiple sclerosis, and thrombophilia. Endocrine 2015;50(2):496-503. - Rosenthal SM. Approach to the Patient: Transgender Youth: Endocrine Considerations. The Journal of Clinical Endocrinology & Metabolism 2014;99(12):4379-89. - Rothkopf AC, John RM. Understanding Disorders of Sexual Development. Journal of Pediatric Nursing 2014;29(5):e23-e34. - Schmidt L, Levine R. Psychological Outcomes and Reproductive Issues Among Gender Dysphoric Individuals. Endocrinology and Metabolism Clinics of North America 2015;44(4):773-85. - Schueftan Gilban DL, Garcia Alves Junior PA, Ricarte Beserra IC. Health related quality of life of children and adolescents with congenital adrenal hyperplasia in Brazil. Health & Quality of Life Outcomes, 2014; v. 12. - Shabir I, Khurana ML, Joseph AA, et al. Phenotype, genotype and gender identity in a large cohort of patients from India with 5α-reductase 2 deficiency. Andrology 2015;3(6):1132-9. - Steensma TD, Kreukels BPC, de Vries ALC, Cohen-Kettenis PT. Gender identity development in adolescence. Hormones and Behavior 2013;64(2):288-97. - Tamar-Mattis A, Baratz A, Baratz Dalke K, Karkazis K. Emotionally and cognitively informed consent for clinical care for differences of sex development. Psychology & Sexuality 2014;5(1):44-55. - Wang LC, Poppas DP. Surgical outcomes and complications of reconstructive surgery in the female congenital adrenal hyperplasia patient: What every endocrinologist should know. The Journal of Steroid Biochemistry and Molecular Biology 2017;165(Pt A):137-44. - Wylie K, Barrett J, Besser M, et al. Good Practice Guidelines for the Assessment and Treatment of Adults with Gender Dysphoria. Sexual and Relationship Therapy 2014;29(2):154-214. - Zainuddin AA, Mahdy ZA. The Islamic Perspectives of Gender-Related Issues in the Management of Patients With Disorders of Sex Development. Archives of Sexual Behavior 2016:1-8. - Zucker KJ, Lawrence AA, Kreukels BPC. Gender Dysphoria in Adults. Annual Review of Clinical Psychology 2016;12(1):217-47. |
| --- |
